# Hydrogen Atom Abstraction via Hydride-Coupled Electron Transfer and Its Origin

**DOI:** 10.1021/acs.inorgchem.5c03613

**Published:** 2025-11-13

**Authors:** Zuzanna Wojdyla, Jishnu Sai Gopinath, Martin Srnec

**Affiliations:** J. Heyrovský Institute of Physical Chemistry, Czech Academy of Sciences, Dolejškova 3, Prague 18223, Czech Republic

## Abstract

This study explores hydride-coupled electron transfer (HCET) as a fundamentally distinct mechanism alternative to proton-coupled electron transfer (PCET). HCET was identified in the reaction between a Cu^III^–OH complex and organic substrates, involving hydride transfer coupled with a reversed electron transfer from Cu^III^–OH to the substrate in a single-barrier step. First, we identified the connection between the thermodynamic cycles and reactivity and showed that the mechanism is dictated by the cycle with more favorable off-diagonal thermodynamics. As evidenced by electronic-structure-based descriptors, the transferred hydrogen atom in HCET gains electron density and volume at the transition state, indicating hydride character, while in PCET, it loses electron density and volume, signaling proton character. Second, intrinsic bond orbital analysis confirmed that HCET is a two-electron process: it involves the complete transfer of the proton and the C–H α-electron from the substrate to the Cu ion, while the β-electron undergoes a transient exchange, initially migrating alongside the α-electron to the Cu center before returning to substrate. An analogous HCET mechanism was identified in the reaction between a Ni^II^–OH complex and TEMPOH, where two β-electrons are engaged in the process: one transiently and one completely transferred to a Ni^II^-coordinating ligand.

## Graphical abstract

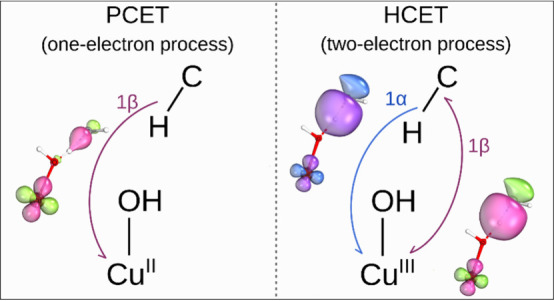

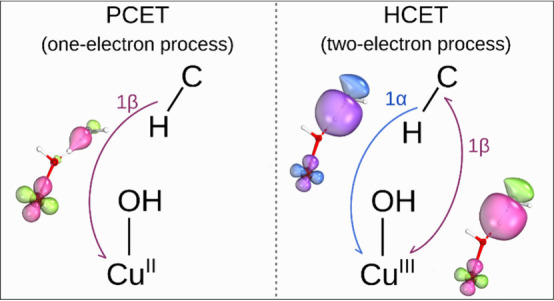

## Introduction

Proton-coupled electron transfers (PCETs) are vital to life, driving essential processes such as energy conversion in the mitochondrial respiratory chain and photosynthesis.^1–4^ In mitochondria, PCET facilitates proton transfer across the inner membrane, creating a proton gradient crucial for ATP synthesis.^5,6^ In photosynthesis, it enables the light-driven splitting of water into oxygen, protons, and electrons, providing the reducing power needed for carbon fixation.^7^

PCET also underpins enzymatic catalysis, enabling key biochemical transformations. For example, nitrogenases use PCET to reduce atmospheric nitrogen to ammonia in nitrogen fixation,^8^ while hydrogenases catalyze the reversible conversion between protons and molecular hydrogen, playing a crucial role in microbial metabolism and hydrogen-based energy storage.^9–11^ PCET is the initial step in a vast range of oxidative transformations (hydroxylation, epoxidation, desaturation, (oxa)­cyclization, epimerization, and halogenation) catalyzed by heme and nonheme iron enzymes.^12–16^ These examples highlight the central role of PCET in energy conversion, redox balance, and life-sustaining biochemical transformations.

In synthetic chemistry, PCET (generally H atom abstraction, HAA) has become essential for advancing the synthesis of natural and pharmaceutical compounds.^17–22^ It enables selective C–H bond activation, which is a transformative approach for constructing complex molecular architectures. Direct C–H functionalization via PCET mechanisms offers efficient strategies for assembling sophisticated molecules with exceptional step- and atom-economy.^23–25^ For instance, the selective C–H oxidation of the terpene sclareolide provides a streamlined, scalable route to derivatives of this potent anticancer adjuvant.^26^

The observed HAA selectivity, namely, which of the multiple C–H bonds in the target molecule is preferentially activated, is determined by the lowest-energy reaction barrier, which in turn is shaped by a variety of physicochemical factors. Among the various influencing factors, one stands out for its versatility and widespread relevance: the linear free energy relationship (LFER). This concept captures the connection between the thermodynamic driving force (i.e., the free energy of reaction (Δ*G*_0_)) and the reaction barrier Δ*G* ^‡^, where a stronger driving force typically corresponds to a lower barrier. While this thermodynamic contribution to reactivity is well-established, our recent work revealed that it is not the sole thermodynamic factor in PCET chemistry at play.

To grasp an additional thermodynamic factor, it is important to recognize that PCET involves two weakly coupled transfer processes—of an electron and a proton—that occur simultaneously in a single step, overcoming a single energy barrier.^27,28^ In addition to LFER between Δ*G* ^‡^ and Δ*G*_0_, i.e., relative energies of the reactant and product (referred to as the diagonal states), the height of the PCET barrier is also shaped by the energetics of two off-diagonal states*,* which are electron-transfer (ET) and proton-transfer (PT) intermediates associated with the stepwise ET-then-PT and PT-then-ET pathways. In a single-step PCET reaction, these off-diagonal states are not directly involved in the reaction pathway because their energies lie above that of the PCET transition state, making them inaccessible along the reaction coordinate. However, as illustrated in Figure 1 (from the middle to the left plot), the higher the energy of the ET and PT states, the greater the free energy barrier for PCET. Conversely, when the energy difference between the two states increases, the barrier decreases (as shown from the middle to the right plot).

**Figure 1:**
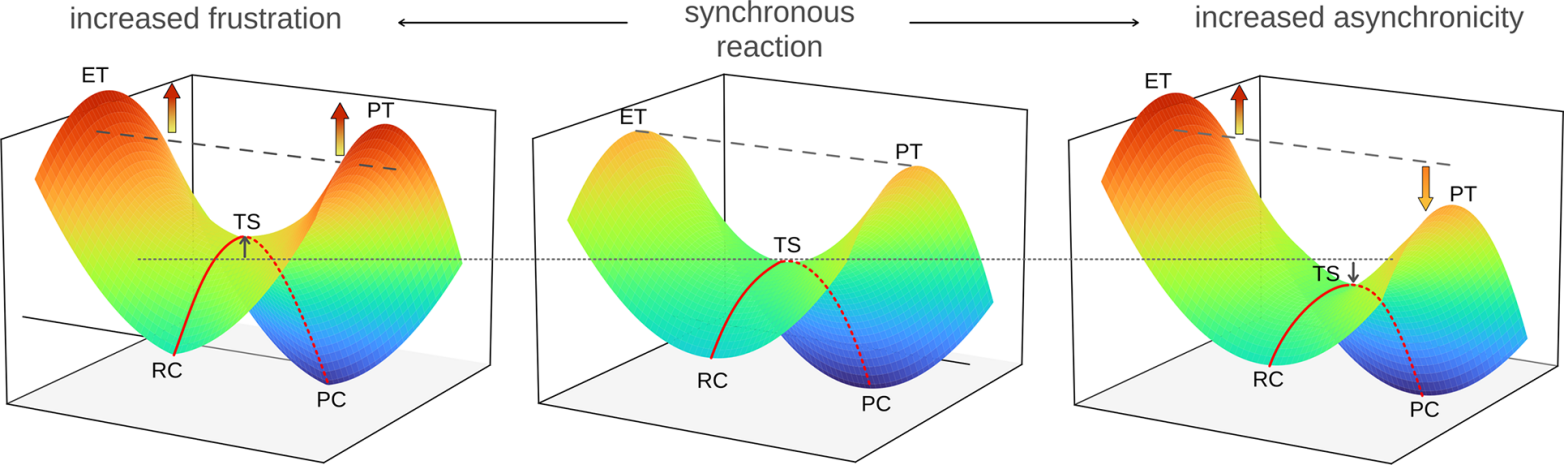
Energetics of the ET and PT states and their effect on the free energy barrier of the PCET reaction in passing from the reactant complex (RC) to the product complex (PC) through the transition state (TS). Energy elevation of the ET and PT states, reflected by larger frustration, increases the PCET barrier (cf. left vs middle plot). Energy imbalance between the ET and PT states, reflected by larger asynchronicity, lowers the PCET barrier (cf. right vs middle plot).

In our recent work, we quantified these effects by introducing two quantities, frustration (σ) and asynchronicity (η), which are described in greater detail in the Theoretical Background section and in refs 29,30 . These off-diagonal parameters were incorporated into a linearized Marcus-type model for the reaction barrier, expressed as

$$\Delta G^{‡}=\frac{\lambda }{4}+\frac{\Delta G_{0}}{2}$$ 1

with λ denoting the Marcus reorganization energy, which is modulated by the off-diagonal contributions according to

$$\lambda =\lambda_{00}+\left|\sigma \right|-\left|\eta \right|$$ 2

leaving λ_00_ to represent the reorganization energy in the idealized limit of a fully synchronous and unfrustrated HAA reaction. This framework ultimately led to the extended expression:

$$\Delta G^{‡}=\Delta G_{00}^{‡}+\Delta G_{\mathrm{diag}}^{‡}+\Delta G_{\mathrm{offdiag}}^{‡}=\frac{\lambda_{00}}{4}+\frac{\Delta G_{0}}{2}+\frac{\left|\sigma |-|\eta \right|}{4}$$ 3

as detailed in refs 29,30 . In this implementation, the nonthermodynamic contribution to the barrier $(\Delta G_{00}^{‡}≡\frac{\lambda_{00}}{4})$ is supposed to contain all factors depending on the reaction coordinate in going from the separated reactants to the transition state (e.g., formation energy of the reactant complex), whereas σ and η constitute together a so-called off-diagonal thermodynamic contribution to the barrier $(\Delta G_{\mathrm{offdiag}}^{‡}≡\frac{\left|\sigma |-|\eta \right|}{4})$ and complement the diagonal one $(\Delta G_{\mathrm{diag}}^{‡}≡\frac{\Delta G_{0}}{2})$ corresponding to LFER. Due to the presence of three distinct thermodynamic contributions in eq 3, we refer to it as the three-component thermodynamic model of reactivity*.*

Importantly, eq 3 reflects a key feature: increasing frustration raises the reaction barrier, thereby slowing the reaction, while greater asynchronicity lowers the barrier and thus makes the reaction faster. Another key aspect of the three-component thermodynamic model is its direct connection to the reaction mechanism. This connection was explored on the related example of the radical ligand transfer (RLT) reactions, more precisely the hydroxyl group transfers.^31^ In that study, we initially demonstrated that the transfer of a hydroxyl radical can be described as a coupled ion–electron transfer process, proceeding through two distinct mechanistic scenarios. In the first scenario, coupled cation–electron transfer analogous to PCET, the electron and the cation species OH^+^ are transferred together in the same direction, from the hydroxyl donor to the acceptor (denoted as unidirectional coupled OH_→_^+^/e_→_^−^ transfer). In contrast, the second scenario, coupled anion–electron transfer*,* involves the electron flowing from the hydroxyl acceptor to the donor, while the anionic species OH^–^ moves concertedly in the opposite direction (denoted as bidirectional coupled OH_→_^−^/e_←_^−^ transfer). The two coupled ion-electron transfer scenarios were shown to be associated with two distinct thermodynamic cycles (Figure 2). Although these cycles share the same diagonal pathway, they differ in their off-diagonal (stepwise) trajectories, connecting reactants and products. As a result, the cycles exhibit distinct degrees of frustration and asynchronicity, leading to different off-diagonal contributions to the reaction barrier ( $\Delta G_{\mathrm{offdiag}}^{‡}$ from eq 3). The key consequence was that the operative mechanism of the transfer of the hydroxyl radical group is then dictated by the cycle yielding a more favorable off-diagonal contribution, $\Delta G_{\mathrm{offdiag}}^{‡}$ . Our previous findings on radical ligand transfer chemistry prompt an intriguing question: could an analogous or similar bidirectional mechanism exist in H atom abstraction reactions? Specifically, is a bidirectional hydride-coupled electron transfer (HCET) (H_→_^−^/e_←_^−^) possible alongside PCET (H_→_^+^/e_→_^−^)? If so, could such a mechanism be linked to favorable off-diagonal thermodynamics? What would be a clear electronic-structure signature for HCET vs PCET? These questions are addressed in the presented work.

**Figure 2:**
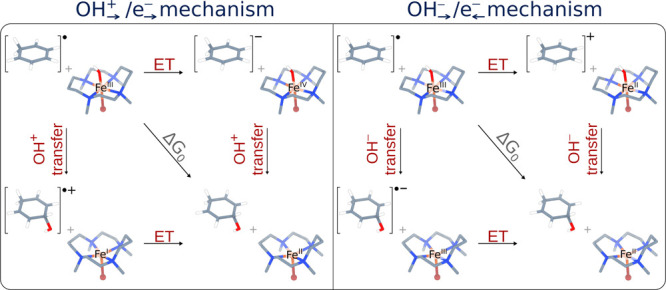
Full-reaction thermodynamic cycles linked to the two studied hydroxyl ligand transfer mechanisms, i.e., for coupled OH_→_^+^/e_→_^−^ transfer (left) and for coupled OH_→_^−^/e_←_^−^ transfer (right), as studied in ref 31.

## Results and Discussion

### Experimental Findings That Raise Questions about the PCET Mechanism

In search of a system that would support the HCET mechanism (i.e., coupled H_→_^−^/e_←_^−^ transfer), we focused on the [L′Cu^III^OH] complex, supported by a tridentate bis­(arylamido)­pyridine ligand L′ as reported by Tolman et al.^32–34^ This experimentally characterized complex features a strongly electron-donating ligand, which stabilizes Cu^III^–OH and, at the same time, lowers its redox potential. The complex exhibits high efficiency in hydrogen atom abstraction when reacting with hydrocarbon substrates, but the low potential suggests that the reaction is not driven by ET to the high-valent Cu^III^ site. These considerations hint at either proton transfer-driven PCET or an as-yet unexplored mechanism involving coupled H_→_^−^/e_←_^−^ transfer as the operative reaction channel. To provide further insights into the mechanism of the reaction (from the PCET perspective), previous computational studies were performed in our group on the [L′Cu^III^OH] complex, yet did not validate PT-driven PCET as an operative mechanism,^30^ thus opening the possibility of HCET as the relevant mechanism.

In a similar vein, a subsequent experimental study of the reaction between [L′Cu^III^–OH] and *para*-substituted phenols demonstrated that the reaction rate is only weakly influenced by the redox potential of the phenols, as evidenced by the shallow slope of the (*RT*/*F*) ln­(*k*) vs *E*_1/2_ plots. Additionally, the analysis eliminated the possibility that the reaction is driven by substrate acidity (or the basicity of Cu^III^–OH), as increasing the acidity within the phenol series surprisingly led to a decrease in the reaction rate.^35^

Another striking example of unexpected reactivity was reported by Garcia-Bosch et al.,^36^ for a mononuclear [LNi^II^OH] complex supported by a tridentate, redox-active bis­(carbamoylphenyl)­amine ligand L. This system was shown to access three oxidation states, [LNiOH]^2–^, [LNiOH]^−^, and [LNiOH]^0^, all described as Ni^II^–OH species, featuring varied oxidation states of the ligand. Notably, [LNi^II^OH]^−^ dehydrogenases TEMPOH at a rate approximately 250 times faster than [LNi^II^OH]^0^, despite the latter being a thermodynamically stronger H^+^/e^–^ acceptor by ∼5 kcal mol^–1^ in bond dissociation free energy. Such a puzzling finding was hypothesized to arise from nonthermodynamic factors (namely, a unique electronic configuration of [LNi^II^OH]^−^) as both complexes are comparable in terms of p*K*_a_ as well as reduction potential. However, such a strong increase in reactivity may indicate that the [LNi^II^OH]^−^-catalyzed reaction may follow a mechanism distinct from canonical PCET.

### Theoretical Background for the Analysis of the PCET vs HCET Mechanism Employed in This Study

Figure 3 depicts two competing full-reaction thermodynamic cycles that share a common diagonal pathway, i.e., the same free energy of reaction (Δ*G*_0_ = −*F* × $E_{H}^{{}^{\circ}}$ ), but they differ in their off-diagonal states, which, as discussed above, determine whether the HAA proceeds via PCET (on the left of Figure 3) or HCET (on the right). To quantify the effect of the off-diagonal thermodynamics on the single-step HAA barrier, the starting point is to deconstruct full-reaction thermodynamic cycles into two half-reaction building blocks (shown in blue and red in Figure 3). These blocks define the experimentally accessible thermodynamic properties of the reaction partners such as reduction potentials (*E*°), acidity constants (*pK*_a_), or hydricities ( $\Delta G^{H^{-}}$ ). These properties, in turn, give rise to two off-diagonal thermodynamic factors: asynchronicity η and frustration σ.

**Figure 3:**
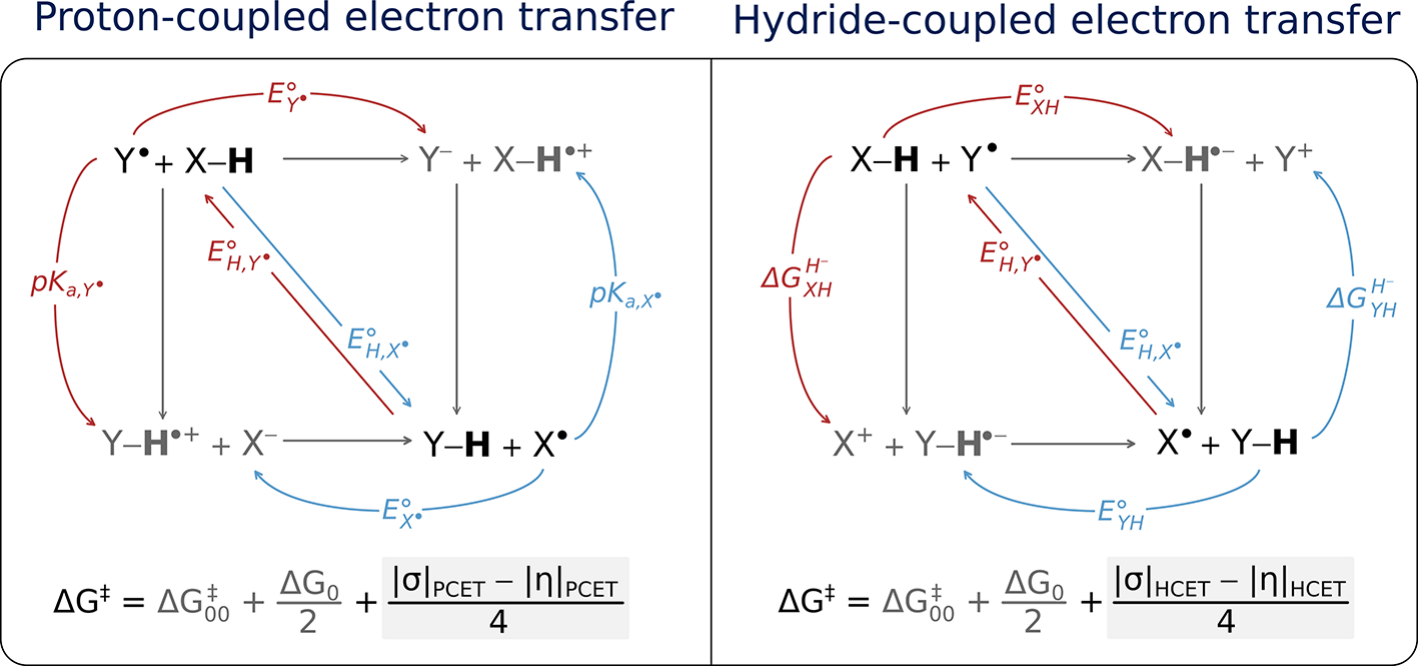
Full-reaction thermodynamic cycle for PCET (left) and HCET (right) between H atom donor X–H and H atom abstractor Y^•^. Each of the full-reaction cycles is deconstructed into respective two half-reaction thermodynamic blocks: one for electron-accepting species Y^•^ and X–H (red arrows in the left and right panel, respectively) and one for electron-donating species X^•^ and Y–H (blue arrows in the left and right panel, respectively). The key energy parameters associated with each half-reaction cycle are as follows: the free energy of one-electron reduction $(\Delta G^{e^{-}}=-F\times E^{{}^{\circ}}$ ) and the free energy of protonation ( $\Delta G^{H^{+}}$ = −*RT* ln(10) × *pK*_a_) for PCET; the analogous free energy of one-electron reduction ( $\Delta G^{e^{-}}$ ) and the free energy of hydride release ( $\Delta G^{H^{-}}$ ) for HCET. The quantities are used to form the composite ones asynchronicity η and frustration σ, which are specific to each of the reaction mechanisms as described in the text by eqs 4–7. For the explicit derivation of σ_PCET_ and η_PCET_ in terms of reduction potentials and acidity constants of X^•^ and Y^•^ and an analogous derivation of σ_HCET_ and η_HCET_ in terms of the reduction potentials and hydricities of X–H and Y–H, see the respective sections in the Supporting Information (and Figure S1). The combined influence of σ_PCET_ and η_PCET_ (σ_HCET_ and η_HCET_) on the reaction barrier (Δ*G* ^‡^) is described by equations shown in the figure and explained in detail in the text accompanying eq 3.

In the case of PCET, both off-diagonal components, η and σ, are dependent on reduction potentials and acidity constants in the form of free energies of reduction and protonation of X^•^ and Y^•^ (Δ*G*_X^•^/Y^•^_^e^−^^ = −*F* × *E*_X^•^/Y^•^_^o^ and Δ*G*_X^•^/Y^•^_^H^+^^ = −*RT* ln(10)­p*K*_a,X^•^/Y^•^_, respectively), while η and σ for HCET result from the combination of reduction potentials and hydricities of X–H and Y–H $(\Delta G_{\mathrm{XH}/\mathrm{YH}}^{e^{-}}=-F\times E_{\mathrm{XH}/\mathrm{YH}}^{{}^{\circ}}\;\mathrm{and}\;\Delta G_{\mathrm{XH}/\mathrm{YH}}^{H^{-}}$ , respectively):

$$\eta_{\mathrm{PCET}}=\frac{1}{\sqrt{2}}[(\Delta G_{Y^{•}}^{e^{-}}-\Delta G_{Y^{•}}^{H^{+}})-(\Delta G_{X^{•}}^{e^{-}}-\Delta G_{X^{•}}^{H^{+}})]$$ 4

$$\sigma_{\mathrm{PCET}}=\frac{1}{\sqrt{2}}[(\Delta G_{Y^{•}}^{e^{-}}+\Delta G_{Y^{•}}^{H^{+}})-(\Delta G_{X^{•}}^{e^{-}}+\Delta G_{X^{•}}^{H^{+}})]$$ 5

$$\eta_{\mathrm{HCET}}=\frac{1}{\sqrt{2}}[(\Delta G_{\mathrm{XH}}^{e^{-}}-\Delta G_{\mathrm{XH}}^{H^{-}})-(\Delta G_{\mathrm{YH}}^{e^{-}}-\Delta G_{\mathrm{YH}}^{H^{-}})]$$ 6

$$\sigma_{\mathrm{HCET}}=\frac{1}{\sqrt{2}}[(\Delta G_{\mathrm{XH}}^{e^{-}}+\Delta G_{\mathrm{XH}}^{H^{-}})-(\Delta G_{\mathrm{YH}}^{e^{-}}+\Delta G_{\mathrm{YH}}^{H^{-}})]$$ 7

We note in passing that this formulation of asynchronicities and frustrations reflects the tug-of-war-like nature of the competition between the two reaction partners, X^•^ and Y^•^, over the components of the hydrogen atom, specifically the H^+^/e^–^ pair in PCET and the H^–^/e^–^ pair in HCET. While we have previously demonstrated and successfully applied this approach,^29–31^ an alternative formulation of the two off-diagonal components also exists. Unlike the tug-of-war depiction of competition between X^•^ and Y^•^, this alternative reflects discrete particle transfers between X–H and Y^•^, specifically ET and PT in the case of PCET and ET and hydride transfer (HT) in the case of HCET, as detailed in the SI. Here, we simply note that the conclusions drawn later in this work are consistent with both formulations of off-diagonal thermodynamics. However, the key difference lies in the fact that $(\Delta G_{00}^{‡}≡\frac{\lambda_{00}}{4})$ from eq 3 derived from the alternative approach can attain negative values, contradicting the conventional assumption from Marcus theory that reorganization energy is always positive.

From the perspective of eq 3, PCET and HCET feature the same barrier contribution $\Delta G_{\mathrm{diag}}^{‡}$ , but they necessarily differ in $\Delta G_{\mathrm{offdiag}}^{‡}\;\mathrm{and}\;\Delta G_{00}^{‡}$ , the latter of which is usually comparably high/low for the set of related reactions. Thus, the operative reaction mechanism should be dictated by the (more) favorable $\Delta G_{\mathrm{offdiag}}^{‡}$ arising from the corresponding thermodynamic cycle from Figure 3. This idea is explored and validated in the following sections.

For the inspection of which mechanism is operative, we applied two independent electronic-structure analyses of transition states of all of the studied HAA reactions. As one approach, we employed the atoms-in-molecules (AIM)^37^ method to quantify charges and volumes of the transferred hydrogen atoms at the respective TSs. Explicitly, the corresponding descriptors, Δ*q* and Δ*V*, read

$$\Delta q=q_{\mathrm{sub}/\mathrm{Cu}}^{\mathrm{TS}}-\frac{1}{2}(q_{\mathrm{sub}/\mathrm{sub}}^{\mathrm{TS}}+q_{\mathrm{Cu}/\mathrm{Cu}}^{\mathrm{TS}})$$ 8

$$\Delta V=V_{\mathrm{sub}/\mathrm{Cu}}^{\mathrm{TS}}-\frac{1}{2}(V_{\mathrm{sub}/\mathrm{sub}}^{\mathrm{TS}}+V_{\mathrm{Cu}/\mathrm{Cu}}^{\mathrm{TS}})$$ 9

These descriptors account for the deviation of charge and volume of the H atom at the TS for the HAA reaction between the substrate and the [L′Cu^II^OH]^−^ or [L′Cu^III^OH] complex ( $q_{\mathrm{sub}/\mathrm{Cu}}^{\mathrm{TS}}/V_{\mathrm{sub}/\mathrm{Cu}}^{\mathrm{TS}}$ ) from the arithmetic mean of H atom charges/volumes at TSs of two corresponding self-exchange HAA reactions:

i. between the C–H substrate and its radical conjugate (denoted as the sub/sub reaction, giving rise to H atom charge/volume parameters $q_{\mathrm{sub}/\mathrm{sub}}^{\mathrm{TS}}$ and $V_{\mathrm{sub}/\mathrm{sub}}^{\mathrm{TS}}$ );
ii. between the [L′Cu^II^OH]^−^ or [L′Cu^III^OH] complex and its hydrogenated form [L′Cu^I^OH_2_]^−^ or [L′Cu^II^OH_2_] (denoted as the the Cu/Cu reaction, giving rise to parameters $q_{\mathrm{Cu}/\mathrm{Cu}}^{\mathrm{TS}}$ and $V_{\mathrm{Cu}/\mathrm{Cu}}^{\mathrm{TS}}$ ).

The referential *q* and *V* quantities calculated for the self-exchange reactions ( $q_{\mathrm{sub}/\mathrm{sub}}^{\mathrm{TS}},V_{\mathrm{sub}/\mathrm{sub}}^{\mathrm{TS}}$ and $q_{\mathrm{Cu}/\mathrm{Cu}}^{\mathrm{TS}},V_{\mathrm{Cu}/\mathrm{Cu}}^{\mathrm{TS}}$ ) are included in equations to separate the effects specific to the reaction mechanism (i.e., partial H^+^ or H^–^ formation) from the ones related to the change of the reaction system geometry common to any X–H bond activation (X = C, O, ...), as discussed more in the SI. Thus, the positive/negative values of Δ*q* and Δ*V* are then indicative of the PCET/HCET mechanism.

Second, we employed intrinsic bond orbital (IBO)^38,39^ calculations to trace the electron flow along the HAA reaction coordinates. As demonstrated by Klein and Knizia, use of IBO analysis allows to differentiate between PCET and H atom transfer (HAT) mechanisms.^40^ In this study, we applied IBO analysis to HAA reactions involving the [L′Cu^III^OH] and [L′Cu^II^OH]^−^ complexes (as well as the two [LNi^II^OH]^0^ and [LNi^II^OH]^−^ complexes) with selected substrates with the attempt to distinguish HCET from PCET, by associating each of the mechanisms with a characteristic evolution of the electrons of the cleaved X–H bond and the OH ligand. To illustrate, one of the key reactions investigated in this study involves the cleavage of the C–H bond by the oxidant. By transforming the α- and β-spin manifold canonical molecular orbitals, obtained from an unrestricted calculation, into localized α- and β-spin manifold IBOs, we can (*i*) easily identify the α/β IBOs for electron pairs of the C–H bond in the reactant complex (RC) and (*ii*) track the corresponding IBOs of these two electrons along the intrinsic reaction coordinate (IRC).

### HAA Reactions of the Tolman’s Cu^III^–OH Complex and Its One-Electron Reduced Form with Various C–H Bond Substrates: Model Systems for Exploring HCET vs PCET Mechanisms

The reactivity of the [L′Cu^III^–OH] complex, experimentally characterized by Tolman and Dhar, has been rationalized by the linear relationship between log­(*k*) and bond dissociation energy (BDE) for the substrate.^33^ However, as discussed in the previous section, the mechanistic basis for the reactivity remains an open topic. From a computational perspective, the Cu^III^–OH system was previously analyzed by us using a three-component thermodynamic model assuming the thermodynamic cycle for the PCET mechanism, which produced anomalous results.^30^ Particularly, once the free energy barrier for abstraction of hydrogen atoms by the Cu^III^–OH was decomposed into contributions according to eq 3, the corresponding nonthermodynamic part $\Delta G_{00}^{‡}$ was found to be negative. Markedly, $\Delta G_{00}^{‡}$ , as the equivalent of the intrinsic barrier from the original Marcus barrier model, quantifies the energetic cost of reaching the transition state for the process in the absence of both the thermodynamic driving force (Δ*G*_0_) and the off-diagonal thermodynamics. As such, it cannot take on a negative value.

#### Off-Diagonal Thermodynamics Associated with the HCET vs PCET Thermodynamic Cycle and Their Barrier Contributions

For HAA reactions between the [L′Cu^III^OH] complex and seven C–H bond substrates, we compared the PCET-derived off-diagonal barrier contribution ( $\Delta G_{\mathrm{offdiag}}^{‡}$ from eq 3) to that from the alternative thermodynamic cycle associated with HCET. We revealed a remarkable contrast: $\Delta G_{\mathrm{offdiag}}^{‡}$ for HCET is significantly more favorable than that for PCET, as demonstrated by positive values of the calculated difference between the two off-diagonal terms in Figure 4 (top).

**Figure 4:**
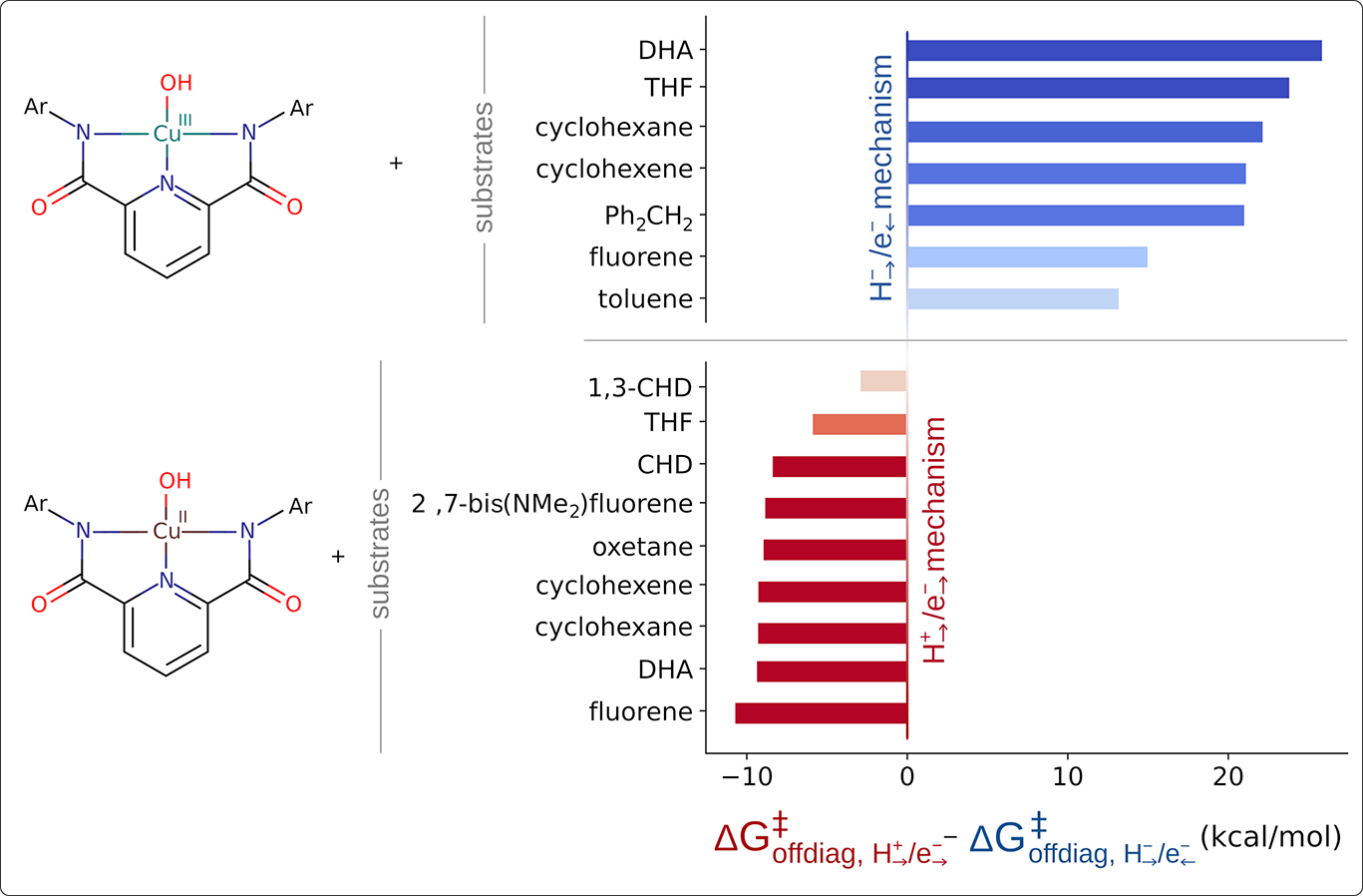
[L′Cu^III^OH] and [L′Cu^II^OH]^−^ complexes and the substrates used in the study, together with the difference between the off-diagonal contributions ( $\Delta G_{\mathrm{offdiag}}^{‡}$ from eq 3) associated with PCET and HCET thermodynamic cycles: negative values indicate preference for the PCET cycle (red) and positive for HCET (blue). For the substrate structures, see Figure S2 in the Supporting Information. The barriers and thermodynamic factors are given in Tables S1–S6.

To support findings derived from the three-component thermodynamic model, we strategically modified the system to alter the reaction mechanism. Specifically, we explored the possibility that a one-electron reduced form of Cu^III^–OH would disfavor hydride transfer, shifting the mechanism to the unidirectional coupled H_→_^+^/e_→_^−^ pathway due to a more favorable $\Delta G_{\mathrm{offdiag}}^{‡}$ . Indeed, the comparison of the $\Delta G_{\mathrm{offdiag}}^{‡}$ values calculated for the two (PCET and HCET) cycles indicates that the unidirectional (PCET) pathway is the operative one in the case of Cu^II^–OH (Figure 4, bottom). For the sake of completeness, we note that [L′Cu^II^OH]^−^ has not been experimentally observed to cleave C–H bonds, which aligns with our computational findings that predict prohibitively high reaction barriers (ranging from 24 to 36 kcal mol^–1^); however, it served as a useful reference system, favoring a PCET mechanism.

Of note, Figures S3 and S4 display the correlation between the reaction barrier Δ*G* ^‡^ and the three-component thermodynamic effect on the barrier ( $\Delta G_{\mathrm{diag}}^{‡}$ + $\Delta G_{\mathrm{offdiag}}^{‡}$ from eq 3), derived from the correct PCET or HCET thermodynamic cycle, alongside a comparison with the correlation when the thermodynamic effect is taken from the incorrect cycle. For HCET reactions involving Cu^III^–OH, the correlation (*R* ^2^) between the reaction barrier and the thermodynamic factor from the HCET thermodynamic cycle is 0.91, whereas the correlation with the thermodynamic factor from the PCET cycle is only 0.66. Similarly, for PCET reactions involving Cu^II^–OH, *R* ^2^ between the reaction barrier and the thermodynamic factor from the PCET thermodynamic cycle is 0.94, whereas *R* ^2^ with the thermodynamic factor from the HCET cycle drops to the value of 0.82.

#### Linking HCET and PCET with Electronic-Structure Descriptors

It is reasonable to expect that systems categorized as HCET or PCET based on off-diagonal thermodynamics can also be differentiated using electronic structure-based descriptors at the TS. Thus, to distinguish the mechanistic signatures specific to a studied reaction from those that are common to all C–H bond cleavage processes (discussed in the SI, Figure S5), we calculated *q* and *V* of the hydrogen moiety at TS with respect to the average of the corresponding *q* and *V* values of the two self-exchange reactions (cf., eqs 8 and 9, respectively). Indeed, as shown in Figure 5, HAA reactions identified as PCET vs HCET exhibit distinctly different values for the descriptors related to the charge and volume of the transferred H moiety at TS.

**Figure 5:**
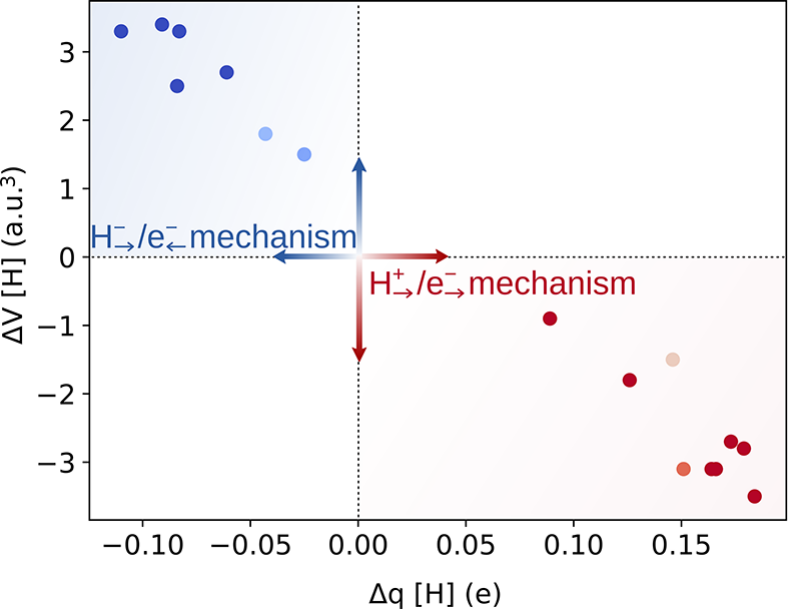
Charge and volume of the transferred H moiety at the TS relative to the referential self-exchange values, as given by eqs 8 and 9. The points are colored and shaded from dark blue (favored H_→_^−^/e_←_^−^) to dark red (favored H_→_^+^/e_→_^−^) to reflect the difference in off-diagonal thermodynamic contributions to the barrier, which originates from the two different H_→_^−^/e_←_^−^ and H_→_^+^/e_→_^−^ cycles presented in Figure 3. The AIM charges and volumes of the studied systems are listed in Tables S7–S13.

Remarkably, HAA reactions of the [L′Cu^III^OH] complex characterized by the off-diagonal contribution favoring the H_→_^−^/e_←_^−^ mechanism form a cluster of points in a single quadrant of the coordinate plane, characterized by a negative value of Δ*q* and a positive value of Δ*V*. The observed decrease in charge and increase in volume relative to self-exchange reactions indicate that the hydrogen atom gains some electron density. Thus, the hydrogen atom can be distinctly understood as acquiring partial hydride character at the TS of C–H bond cleavage. In contrast, the points corresponding to HAA reactions of the [L′Cu^II^OH]^−^ complex occupy the opposite quadrant, characterized by a positive and negative value of Δ*q* and Δ*V*, respectively, which strongly suggests that the H atom develops an electron deficiency at the TS, in line with the protonic character of the transferred H moiety in the coupled H_→_^+^/e_→_^−^ mechanism.

Moreover, the Δ*q* descriptor together with asynchronicity η offers important quantitative insights into the mechanism of the reaction. In both HCET and PCET, the sign of η reflects the character of the imbalance, i.e., whether electron transfer or proton/hydride transfer leads as the component of the driving force, while its magnitude reflects the extent of the imbalance. As the asynchronicity becomes more positive in HCET, hydride transfer is increasingly favored over electron transfer. Conversely, more negative values of η indicate a growing preference for electron transfer over hydride transfer. In the case of PCET, a positive shift in η favors proton transfer, whereas a negative shift enhances the preference for electron transfer. Particularly, for the Cu^III^–OH-promoted HCETs, η_HCET_ indicates that the hydride transfer is favored over ET, whereas Cu^II^–OH-promoted PCETs are driven by proton transfer, in line with the high basicity of the [L′Cu^II^OH]^−^ complex reported by Tolman and Dhar.^33^ These thermodynamic insights are further supported by the Δ*q* descriptor from eq 8: HCET systems with greater asynchronicity favoring hydride transfer display more negative Δ*q* values at TS, while PCET systems with asynchronicity favoring proton transfer exhibit more positive Δ*q* values (as presented in Figure S6 in the Supporting Information).

#### Intrinsic Bond Orbital Analysis of PCET vs HCET Mechanisms

IBO analysis provides insight into the electron flow along the reaction coordinate,^38,39^ making it a powerful tool for differentiating between reaction mechanisms. It has previously been applied to the Tolman’s [L′Cu^III^–OH] complex to demonstrate that hydrogen atom abstraction can proceed via either HAT or PCET, dependent on the substrate (DHA vs 2,6-di-*tert*-butylphenol).^41^

In this study, the evolution of IBOs along the reaction coordinate allows us to distinguish between HCET and PCET, further supporting the observations based on off-diagonal thermodynamics and charge/volume-derived descriptors. As discussed in the previous section, the HCET mechanism can occur in two distinct scenarios, differentiated by asynchronicity. In the first scenario, the primary component of the driving force arises from electron transfer from the Cu^III^–OH complex to the substrate, while in the second, hydride transfer is the dominant component. In the former case, IBOs would likely show electron transfer in the opposite direction compared to that in the PCET mechanism, while in the latter, one would expect a two-electron/proton transfer (equivalent to H^–^) from the substrate to Cu^III^–OH. Notably, the second scenario, characterized by a partial hydride transfer, is observed in the studied HCET reactions (see below), aligning with the prediction based on the asynchronicity factor derived from the appropriate thermodynamic cycle.

The IBOs involved in the HAA reactions of both [L′Cu^II^OH]^−^ and [L′Cu^III^OH] complexes are associated with the flow of two electrons initially occupying α and β IBOs of the substrate C–H σ bond, as shown in Figure 6. The Cu^II^–OH-promoted HAA features a pattern well-recognized for PCET (in the left panel in Figure 6): the α-electron of the C–H σ bond in cyclohexane remains on the carbon atom, while the β-electron of the C–H σ bond transfers to the Cu center, reducing Cu^II^ to Cu^I^. Concurrently, the oxygen lone pair in the Cu complex accepts the proton, forming an O–H bond. The proton and electron are accepted by distinct sites, accordingly with the PCET mechanism.

**Figure 6:**
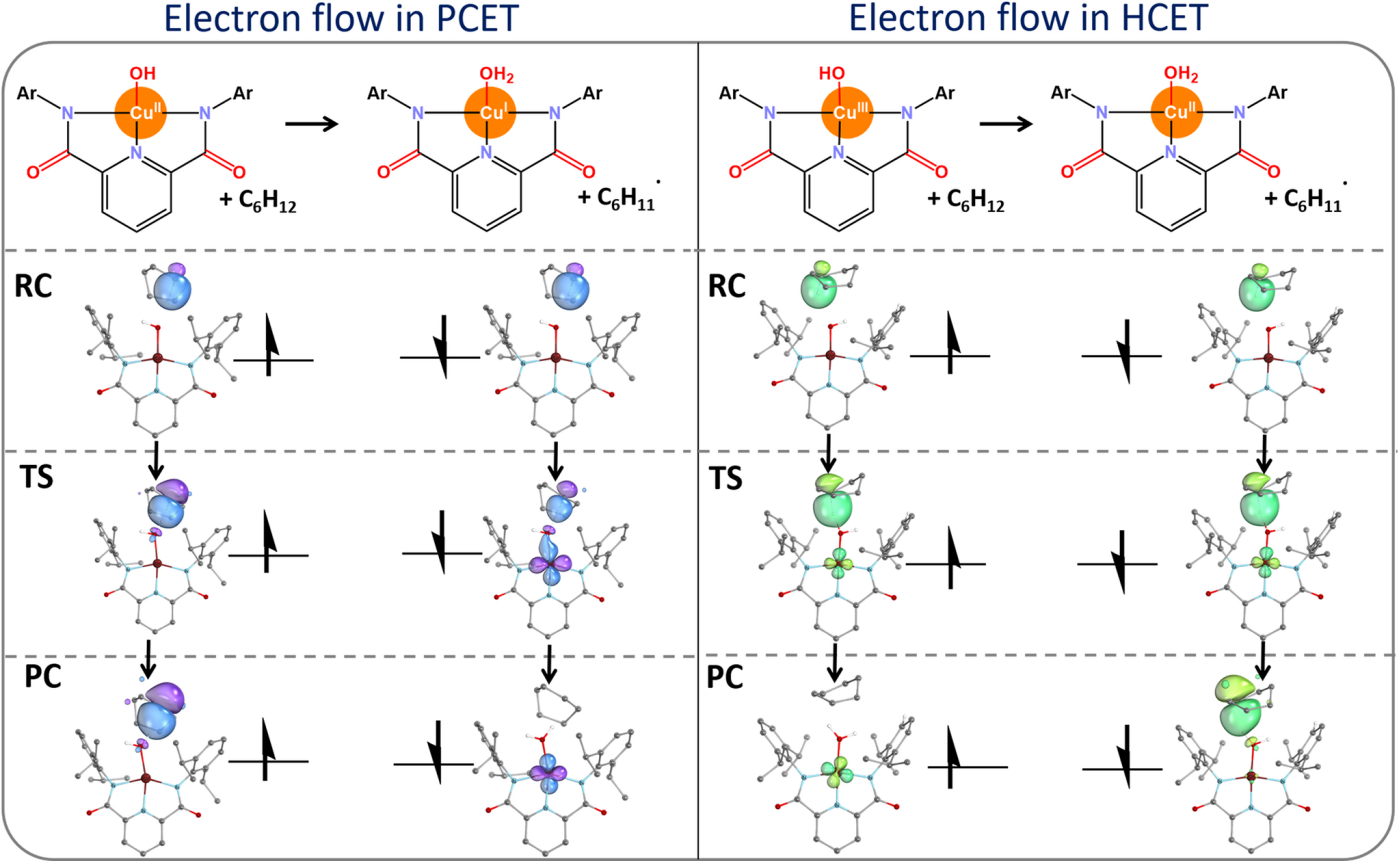
Ion-coupled electron transfer reaction between cyclohexane with the Cu^II^–OH oxidant (left) and with the Cu^III^–OH (right): the corresponding reactions and the key IBOs involved in the [Cu^II^–OH:cyclohexane] and [Cu^III^–OH:cyclohexane] reaction systems are shown. The IBOs undergoing significant changes can be identified from a plot of the evolution of orbitals in going from the reactant to the product complex (Figure S7). A similar scenario exists for the reactions of both the Cu^II^–OH and Cu^III^–OH complexes with the remaining substrates (Figures S8 and S9, respectively). For clarity, hydrogen atoms are omitted from the molecular representations.

Unlike the Cu^II^–OH-promoted PCETs, the HAA reactions performed by Cu^III^–OH exhibit a unique, more complex pattern, where both of the C–H bond electrons participate in the reaction (in the right panel in Figure 6). More specifically, this panel depicts the evolution of the α- and β-electrons that initially constitute the C–H σ bond in the RC, as the system advances along the reaction coordinate toward the TS and subsequently to the PC. At the RC, the pair of α- and β-electrons is localized within the C–H bond of cyclohexane. At the TS, the α- and β-electrons are still paired and are partially delocalized to the Cu center. This indicates a concerted hydride transfer mechanism from the substrate to the Cu complex. However, in going from TS to PC, the fates of the two α- and β-electrons diverge: the β-electron in the post-TS phase of the reaction is relocalized back to the carbon atom of the substrate, while the α-electron completes its transfer to the metal.

Thus, the distinction between the PCET and HCET mechanisms lies in the flow of the α and β-electrons. In PCET, the β-electron is completely transferred while the α-electron remains localized on the C atom along the whole reaction trajectory, whereas in HCET, the β-electron undergoes a transient transfer, as a component of the H^–^, moving toward the copper atom up to the TS, before being transferred back to the carbon atom of the donor molecule while the α-electron is completely transferred.

We may speculate that a similar HCET mechanism may be operative for the related Cu^III^–fluoride complex, which was demonstrated, based on the intrinsic atomic orbital localization method, to feature a combination of Cu^II^–F^•^ and Cu^I^–F^+^ resonance structures. The latter structure may facilitate hydride transfer, similarly to the Cu^III^–OH complex.^42^

### HAA Reactions of the Tolman’s Cu^III^–OH Complex with a Series of *para*-Substituted Phenols: Switch from PCET to HCET

A thermodynamic cycle-based analysis of the reaction between Cu^III^–OH and a series of *para*-substituted phenols reveals that the mechanism can shift from PCET to HCET as a result of systematic modifications within a set of related substrates. This finding complements the PCET-to-HCET transition, which was driven by altering the oxidation state of the Cu center, as discussed in detail in previous sections.

The comparison of the off-diagonal thermodynamic energetics associated with PCET and HCET indicates that phenols featuring strongly electron-withdrawing substituents (−NO_2_ and −CF_3_) show preference toward PCET, whereas introduction of electron-donating (or less electron-withdrawing) groups switches preference toward HCET (Figure S10A). This dependence can be rationalized once the asynchronicity of the reaction is considered. In particular, asynchronicity indicates that HCETs involving phenols, in full analogy to HCET with the C–H substrates, is driven by hydride transfer from the phenol to the [L′Cu^III^OH] complex. Consequently, the introduction of electron-withdrawing groups disfavors hydride donation, ultimately promoting a mechanistic shift toward PCET, which, according to asynchronicity, favors PT over ET between the reactants. Thus, the introduction of electron-withdrawing groups plays a dual role: not only suppresses transfer of a hydride but also enhances the acidity of the phenol, making PT more favorable and supporting the PCET pathway.

This picture is supported by an analysis of IBOs, which reveals a pattern characteristic of PCET in the case of the CF_3_-substituted phenol (in the left panel of Figure 7), though with slightly greater complexity as provided in details in Figure S11B. In contrast, the reaction involving the OMe-substituted phenol shows a distinct two-electron HCET pattern, with one β-electron of the substrate π bond migrating to the Cu site and one α-electron undergoing transient transfer to the Cu site before finally returning to the substrate (in the right panel in Figure 7).

**Figure 7:**
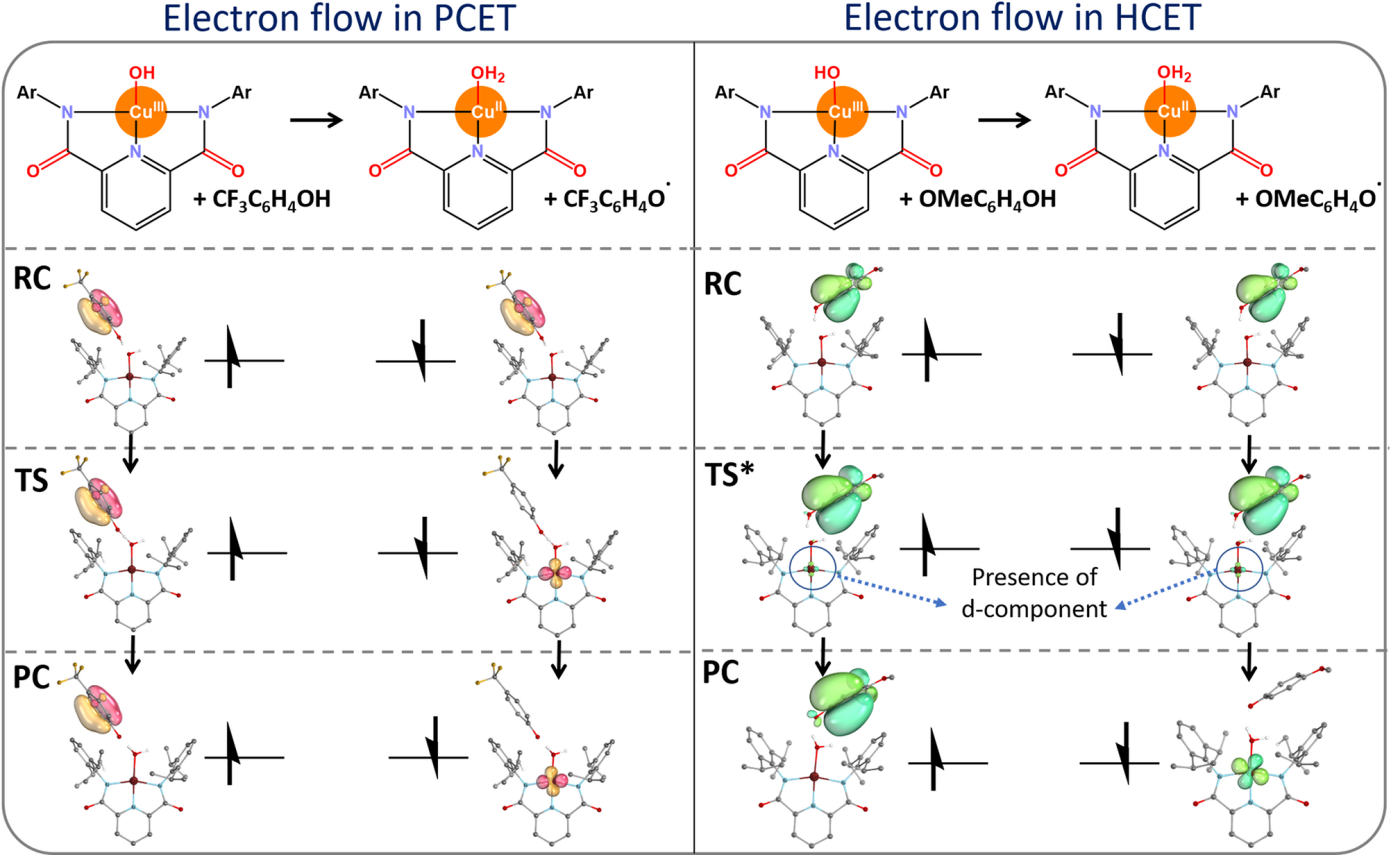
IBO analysis illustrating the PCET mechanism for the reaction between the *p*-trifluoromethylphenol and the Tolman’s Cu^III^–OH oxidant (left), as well as the HCET mechanism between *p*-methoxyphenol and the same oxidant (right). TS* indicates the structure along the reaction coordinate just before the transition state, and the d-orbital component in TS* is highlighted for a better understanding. Note that for clarity, the molecular systems are shown in a simplified form (no hydrogen atoms visualized). For the extended analysis, see Figure S11 in the Supporting Information.

Admittedly, analysis of AIM Δ*q* and Δ*V* does not yield conclusive results, as the charge and volume at the TS are comparable to the reference derived from self-exchange reactions (Figure S10B) and thus do not allow unambiguous distinction between the mechanisms. This behavior can be explained by lower asynchronicity of the Cu^III^–OH reaction with phenols as compared to C–H substrates (Table S3), which presumably results in a less pronounced charge separation during the process. A decreased asynchronicity also aligns with a minor contribution from the d-orbital component to the critical IBO at the transition state, as illustrated in Figure 7.

### HAA Reaction of a Ni^II^–OH Radical Complex with TEMPOH Proceeds via HCET

In light of the unexpectedly high reactivity of the [LNi^II^OH]^−^ toward HAA from TEMPOH in comparison to [LNi^II^OH]^0^, we explored the possibility that the two complexes follow two distinct mechanisms. Indeed, an evolution of the key IBOs along the reaction trajectory for HAA from TEMPOH by a neutral [LNi^II^OH]^0^ is fully consistent with that of PCET (in the left panel in Figure 8). In this case, the β-electron originating from the nitrogen lone pair of TEMPOH is fully transferred to the ligand unit associated with the Ni complex, along with the proton transfer indicating classic PCET. On the other hand, the IBOs for the HAA between a [LNi^II^OH]^−^ and TEMPOH corroborate the HCET mechanism (in the right panel in Figure 8). The observed pattern is similar in principle to that observed in the Cu^III^–OH system; however, some differences are evident. In the Cu^III^–OH system, the α-electron of the C–H bond is fully transferred to the Cu center, while the β-electron experiences a transient forward-then-back transfer. In contrast, for the [LNi^II^OH]^−^ system, the α-electron originating from the O–H bond of TEMPOH remains localized on the oxygen atom throughout the reaction and the β-electron from the same bond follows a “bouncing” transfer pathway: at the TS, it is partially transferred to a Ni^II^-coordinating ligand before ultimately returning to the oxygen atom to form a π bond in the substrate radical. Simultaneously, another β-electron originating from the nitrogen lone pair of TEMPOH is fully transferred to the same ligand unit associated with the Ni complex. Overall, the process involves the transfer of a hydride unit (H^+^ and two β-electrons) from the substrate, coupled with a reverse electron transfer involving one of the β-electrons, indicating an HCET mechanism distinct from the Cu^III^–OH system presented in Figures 6 and 7.

**Figure 8:**
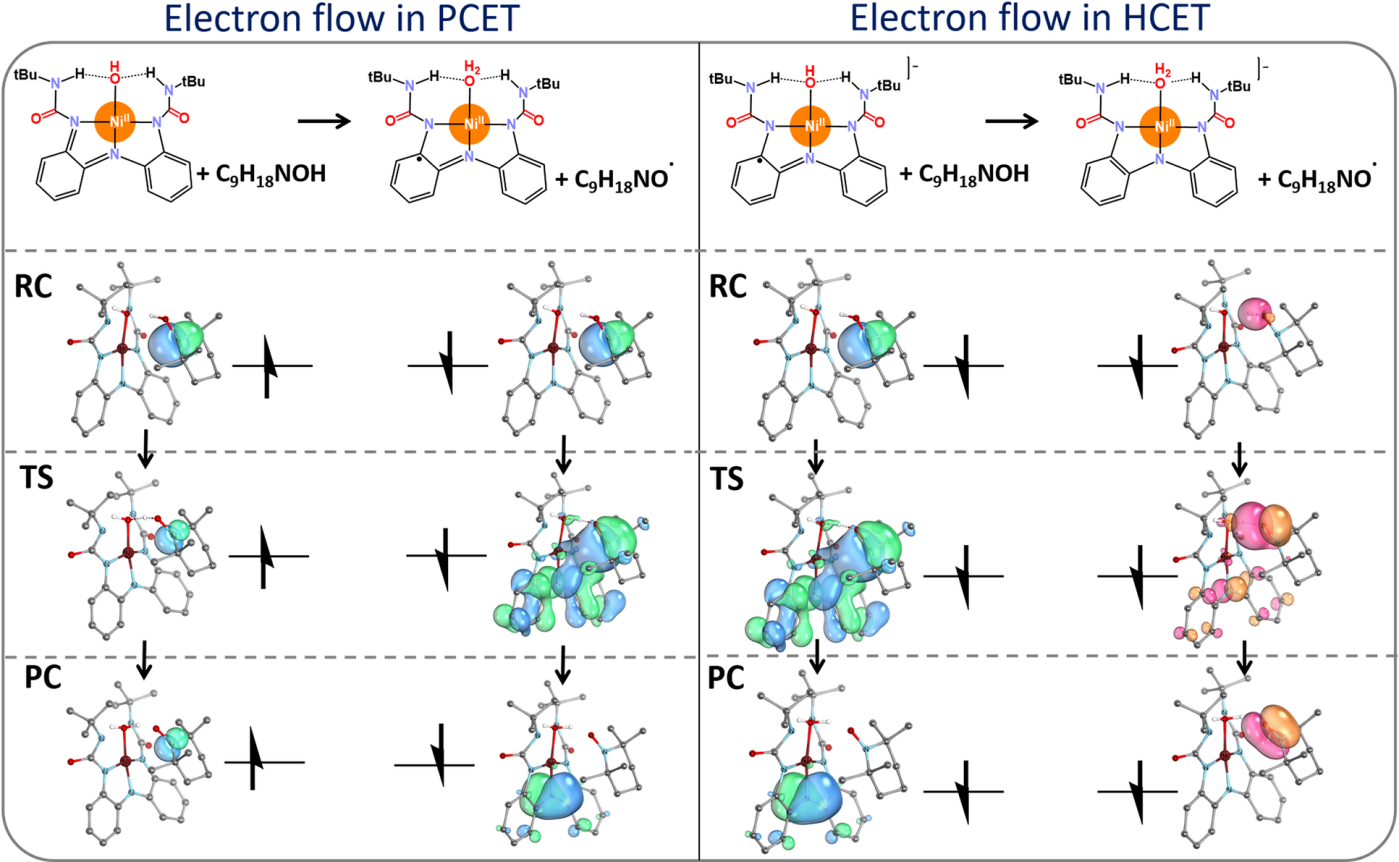
IBO analysis illustrating the PCET mechanism for the reaction between the TEMPOH substrate and the [LNi^II^OH]^0^ oxidant (left), as well as the HCET mechanism between TEMPOH and the [LNi^II^OH]^−^ oxidant (right). For the sake of clarity, the molecular systems are shown in a simplified form (no hydrogen atoms visualized). For the extended analysis, see Figure S12 in the Supporting Information.

The seemingly counterintuitive observation that the anionic form [LNi^II^OH]^−^ undergoes HCET, while the neutral [LNi^II^OH]^0^ favors PCET, can be explained by the fact that 1β transfer is more advanced in PCET (with 0.41e^–^ transferred to the neutral oxidant at the TS, according to AIM analysis) compared to the concerted 2β transfer in HCET with 0.28e^–^ transferred to the anionic oxidant. In the case of both the [LNi^II^OH]^−^ and [LNi^II^OH]^0^ systems, the mechanism identified in the IBO analysis aligns with the prediction based on the off-diagonal thermodynamics, as discussed in detail in the SI.

We note that, while the off-diagonal thermodynamic prediction for the mechanism is internally consistent with the calculated reaction pathway, the calculations do not match the experimental observation that the [LNi^II^OH]^−^ complex reacts faster with TEMPOH than does [LNi^II^OH]^0^. We conclude that this discrepancy may stem from some inaccuracies in the B3LYP-computed energetics of the Ni^II^–OH species, as noted in the original study,^36^ or from other factors, such as the direct involvement of a solvent molecule or differences between the crystallographic structure and the model, including ligand conformational flexibility and altered hydrogen bonding around the Ni–OH group. Partially, tunneling can also be a factor. Additionally, the presence of nearby amine groups of the ligand suggests that the H moiety could be accepted by one of these sites.

Of final note, another interesting example of a Ni^III^ complex, which is more reactive toward hydrogen atom abstraction from the O–H bond than expected based on the linear free energy relationship, was recently reported.^43^ The reactivity of the system was attributed to larger asynchronicity toward proton transfer in comparison to the related Cu^III^ system; however, it may be possible that the reaction with Ni^III^ proceeds via the HCET mechanism similar to the one described in this section.

### Analogies and Differences between Bidirectional H_→_^−^/e_←_^−^ and OH_→_^−^/e_←_^−^ Reactions

A bidirectional OH_→_^−^/e_←_^−^ mechanism is an established route for OH rebound reactions in (bio)­inorganic systems.^44–49^ The off-diagonal thermodynamic-based contributions to the OH rebound activity were studied for a series of OH rebound reactions between the axially substituted and tetramethylcyclam-supported (X)­(TMC)­Fe^III^–OH complexes and the cyclohexadienyl radical.^31^ The study demonstrated the link between the reaction mechanism and a thermodynamic cycle with a more favorable off-diagonal contribution to the barrier. In parallel, it provided electronic-structure descriptors characterizing the mechanism (as in eqs S43 and S44).^31^ To gain a deeper understanding of the coupled OH_→_^−^/e_←_^−^ transfer in relation to the presented HCET, we also explore here the evolution of IBOs during the reaction (Figure S13). Our findings support the bidirectional transfer of the OH^–^ fragment from Fe^III^–OH to the cyclohexadiene radical, alongside the concurrent transfer of an unpaired β π-electron from the radical to the Fe center.

However, this OH rebound mechanism differs noticeably from the HCET mechanism observed for [L′Cu^III^OH] or [LNi^II^OH]^−^. In the OH_→_^−^/e_←_^−^ case, three electrons participate in the hydroxyl group transfer: a pair of electrons from the Fe–OH bond and one unpaired electron from the cyclohexadienyl radical. Unlike in the H_→_^−^/e_←_^−^ mechanism, the electron transferred back from the radical to the Fe center is distinct from the two electrons transferred from the Fe^III^–OH complex.

The OH_→_^−^/e_←_^−^ transfer clearly resembles the reported concerted fluoride electron transfer by Xue and co-workers, where one electron is transferred from the boryl radical to the F donor and two C–F σ-electrons are used to form a new B–F bond.^50^

Since IBO analyses suggest hydroxide-coupled electron transfer to be classified as a three-electron transfer process, while hydride-coupled electron transfer is more accurately described as a two-electron transfer process, it may also explain why the Δ*q* and Δ*V* descriptors from eqs 8 and 9, which are used to distinguish HCET from PCET, differ from the descriptors in eqs S43 and S44, which were employed to differentiate bidirectional OH_→_^−^/e_←_^−^ from PCET-analogous unidirectional OH_→_^+^/e_→_^−^ transfer. Here, we speculate that three-electron HCET is possible to exist, provided that hydride transfer is more spatially separated from electron transfer.

## Conclusions

In this study, we investigated an alternative mechanism to proton-coupled electron transfer (PCET): hydride-coupled electron transfer (HCET), also described as a bidirectional H_→_^−^/e_←_^−^ reaction. This mechanism was found to operate in the reaction between a [L′Cu^III^OH] complex and various organic substrates, involving the transfer of a hydride between the reactants, coupled in a single reaction step with electron transfer back from Cu^III^–OH to the substrate. To distinguish between HCET and PCET mechanisms, we employed the thermodynamic cycles along with a three-component thermodynamic framework that directly links the energetics of off-diagonal states, namely, electron transfer (ET) and proton or hydride transfer (PT or HT) states, to the free energy barrier of the reaction. Recognizing that the accessibility of these off-diagonal states influences the reaction barrier, we proposed that the HCET mechanism operates due to a more favorable combined effect of thermodynamic frustration and asynchronicity on the reaction barrier. Given that HCET and PCET exhibit distinct off-diagonal states, this approach allowed us to identify HCET as the preferred mechanism in the Cu^III^–OH system. We further unambiguously linked the two distinct mechanisms to electronic-structure-based descriptors derived from the AIM H atom charges and volumes. These descriptors revealed that the transferred hydrogen atom in HCET gains electron density and volume at the TS relative to self-exchange reaction cognates (i.e., partially adopts hydride character), whereas the hydrogen atom in PCET loses electron density and volume, exhibiting proton character.

This conclusion was further supported by analyzing electron flow along the reaction coordinate using intrinsic bond orbitals. The reaction between Cu^II^–OH and the donor follows the conventional PCET mechanism, in which the proton and β-electron from the substrate C–H bond are transferred unidirectionally to the Cu complex, while the α-electron from the same bond remains on the donor fragment. In contrast, the reaction involving Cu^III^–OH follows a distinct HCET mechanism, where both the proton and the C–H α-electron are fully transferred from the substrate to the Cu complex. Meanwhile, the β-electron undergoes a temporary exchange, first moving to the Cu center before returning to the substrate as the reaction progresses. In addition, we demonstrated that the mechanistic PCET-to-HCET switch can be achieved via systematic modifications within a series of related *para*-substituted phenols reacting with Cu^III^–OH. Alternatively, HCET may involve two β-electrons: one fully transferred and the other undergoing a transient migration to the metal complex, as demonstrated for the Ni^II^–OH system.

This HCET mechanism was further compared to the OH rebound pathway, which also involves bidirectional anion/electron transfer. However, the bidirectionality in each case differs: in HCET, it results from the complete transfer of one electron and the transient exchange of another, while in the OH rebound mechanism, three separate electrons are involved in the anion and electron transfer steps (Scheme 1).

**Scheme 1:**
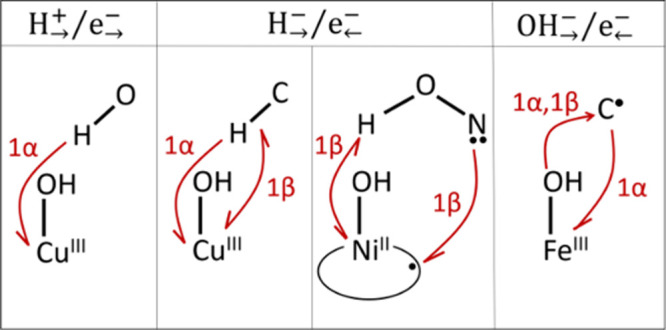
Modus Operandi of the Bidirectional X_→_^−^/e_←_^−^ Mechanism in the Presented Hydride-Coupled Electron Transfer (with X = H, Middle Panel) and Hydroxide-Coupled Electron Transfer (X = OH, Right Panel) Studied in Ref 31 and the Canonical PCET Process Involving the Transfer of a Single Electron (Left Panel)^a^

a HCET is characterized as a two-electron process in contrast to the OH rebound, which involves three electrons. A single fish-hook arrow, a double-sided fish-hook arrow, and the standard arrow represent one-electron transfer, transient one-electron transfer, and two-electron transfer, respectively.

## Computational Details

The calculations were performed using the Gaussian 16 revision C.01 program^51^ for the [L′Cu^III^OH] complex (L′ = *N*,*N*′-bis­(2,6-diisopropylphenyl)-2,6-pyridinedicarboxamide) as reported by Tolman et al.,^33,34^ for the one-electron reduced variant of this complex and for the [LNi^II^OH]^0/−^ complexes (L = *N*,*N′*-bis­[2-(*tert*-butylcarbamoylamino)­phenyl]­amine). The B3LYP functional^52^ with Grimme’s D3 dispersion correction^53^ and the def2-SVP basis set^54^ was used. The solvation effects were described with the conductor-like polarizable continuum model (CPCM)^55^ using ε = 7.4 (tetrahydrofuran) for the Cu^III^–OH/Cu^II^–OH complexes and ε = 37.2 (*N*,*N*-dimethylformamide) for Ni^II^–OH complexes. It is worth noting that the def2-SVP basis set is considered reasonable, as it yields energetics and electronic structures very similar to those obtained with the larger def2-TZVP basis set (Tables S3 and S4).

The Gibbs free energies for the optimized structures were calculated as the sum of potential electronic energies (*E* ^el^) calculated at the B3LYP-D3/def2-SVP level with CPCM and the thermal enthalpic and entropic contributions to the Gibbs free energy (at 298.15 K) obtained from frequency analysis performed at the same level of theory: *G* = *E* ^el^ + [*E* ^ZPE^ + *pV* + *RT* ln *Q*], where *E* ^el^ and *Q* are the zero-point vibrational energy and the molecular partition function, respectively.

To calculate species corresponding to the half-reaction thermodynamic cycles and the barriers for the reaction, the ground states of the reactants were used unless stated otherwise: the singlet for [L′Cu^III^OH] and [LNi^II^OH] and the doublet for [L′Cu^II^OH]^−^ and [LNi^II^OH]^−^. The Gibbs free energy barriers for the reactions were calculated as the difference between the Gibbs free energy of TS and the isolated reactants. A value of 1.9Δ*n* kcal mol^–1^ has been applied to correct the computed values to the 1 mol L^–1^ standard state (a value of 1.9 kcal mol^–1^ corresponds to the conversion of a 1 bar standard state in the gas phase to a 1 mol L^–1^ concentration in solution at 298 K; Δ*n* is the change in the number of moles). To obtain the TS structures for the self-exchange reactions between the [L′Cu^II^OH]^−^ or [L′Cu^III^OH] complex and its hydrogenated form ([L′Cu^I^OH_2_]^−^ or [L′Cu^II^OH_2_]), the bulky 2,6-diisopropylphenyl groups were replaced by methyl groups. The TS structures were approximated by constrained geometries that imposed symmetry between the two Cu complexes: the corresponding Cu–X distances (where X refers to the ligating N atoms and the O atom of the OH/OH_2_ group) were constrained to be equal across the pair, without fixing their absolute values. Similarly, the distances between the transferring hydrogen atom and the donor and acceptor oxygen atoms were constrained to be equal, to obtain a symmetric hydrogen-sharing configuration. For the purpose of locating the TS structure of the self-exchange reaction between [LNi^II^OH] and [LNi^II^OH_2_], as well as between [LNi^II^OH]^−^ and [LNi^II^OH_2_]^−^, only the bulky *t*-butyl substituent was replaced with a methyl group.

The atoms-in-molecules (AIM) approach implemented in the AIMAll program^37^ was employed to assess the redistribution of electron density during the reaction in the investigated systems. The atomic charges were computed based on the electron densities calculated at the B3LYP-D3/def2-SVP level for the optimized structures of the transition states and reactant complexes. Densities were integrated using the Proaim method with a “very fine” interatomic surface mesh and a basin outer angular quadrature of 14,400 grid points (using 15-point Gaussian quadrature GS15). The AIM properties of the atoms with a Lagrangian L­(A) > 0.001 au were recalculated with the Promega algorithm. The AIM charges and volumes (calculated for the isodensity surface of 0.002 au) of the defined group of atoms, that is, the transferred hydrogen atom, the H atom donor, and the acceptor [L′Cu^III^–OH] or [L′Cu^II^–OH]^−^ complex, were used to characterize the reaction.

Intrinsic reaction coordinate (IRC) structures were derived from the corresponding TSs employing the same computational level as that used for geometry optimization. To investigate the electron flow, we utilized IboView (iboexp = 2)^38,39^ to generate and analyze intrinsic bond orbitals based on the wave functions obtained from the IRC points.

## Supplementary Material





## Data Availability

Data is available in 10.5281/zenodo.15296283.

## Notes

Computational details; Gibbs free energies of investigated species; thermodynamic and reactivity data for the substrates and the [L′Cu^III^OH]^−^, [L′Cu^II^OH]^−^, and [LNi^II^OH]^0/–^ complexes; scheme of oxidants and substrates used in the study; additional analysis of performance of the three-component model, electronic-structure descriptors, changes of intrinsic bond orbitals along the reaction coordinate, and structural features of the transition states for HAA reactions (PDF)
Data set collection of computational results (including transition state structures and reactant and product complexes) (TXT)
