# Supplementary material for: Hydrogen Atom Abstraction via Hydride-Coupled Electron Transfer and Its Origin
Source: Inorg Chem. 2025 Nov 13;64(46):22698–710. doi: 10.1021/acs.inorgchem.5c03613 (PMC12648667; doi:10.1021/acs.inorgchem.5c03613)
Supplement: Supplementary file 1 [file ic5c03613_si_001.pdf]

Supplementary Information

Hydrogen atom abstraction via hydride-coupled electron transfer and its origin

Zuzanna Wojdyla,[a] *Jishnu Sai Gopinath*[a] *and Martin Srnec*[a,*]

[a]J. Heyrovský Institute of Physical Chemistry, Czech Academy of Sciences, Dolejškova 3, 18223 Prague, Czech Republic

Corresponding Author

*E-mail: martin.srnec@jh-inst.cas.cz

Table of Contents

Off-diagonal contributions to HCET expressed in terms of the reduction potentials and hydricities of X–H and Y–H S3

Off-diagonal contributions for HCET expressed in terms of full reaction cycles S4

Off-diagonal contributions to PCET expressed in terms of the reduction potentials and pK_a values of $X^\bullet$ and $Y^\bullet$ S9

Off-diagonal contributions for PCET expressed in terms of full reaction cycles S11

Thermodynamic and reactivity data for the substrates and Cu(III)-OH/ Cu(II)-OH complexes S14

Oxidants and substrates used in the study S27

Performance of the three-component model applied to Cu(III)-OH/ Cu(II)-OH reactions S28

Analysis of the reactions based on electronic-structure descriptors S30

Intrinsic bond orbital analysis of the HAA reactions S34

HAA involving para substituted phenols S41

HAA involving the [LNi^{II}OH] complexes S45

Intrinsic bond orbital analysis of the OH rebound reaction S48

Structural features of the transition states and adiabaticity of the HAA reaction S49

Off-diagonal contributions to HCET expressed in terms of the reduction potentials and hydricities of X–H and Y–H

Hydricity ($\Delta G_{XH}^{H^-}$) is the free energy of heterolytic X–H bond dissociation to yield a parent species X^+ and the hydride anion H^-:

$$\Delta G_{XH}^{H^-} = G_{X^+} + G_{H^-} - G_{XH} \quad \text{(S1)}$$

The one-electron reduction potential (E°) is a quantity reflecting Gibbs free energy change upon $1e^-$ reduction of a solute (X–H):

$$F \cdot E_{XH}^\circ = G_{ox} - G_{red} - E_{abs}^\circ(\text{reference}) = G_{XH} - G_{XH^{\bullet -}} - E_{abs}^\circ(\text{reference}) \quad \text{(S2)}$$

Note that E_{abs}°(reference) is the absolute potential of the reference electrode. Since this is constant, it can be omitted in derivation of asynchronicity and frustration.

Asynchronicity η defined in Eq (6) in the main text as:

$$\eta_{HCET} = \frac{1}{\sqrt{2}}[(\Delta G_{XH}^{e^-} - \Delta G_{XH}^{H^-}) - (\Delta G_{YH}^{e^-} - \Delta G_{YH}^{H^-})] \quad \text{(S3)}$$

can be transformed to:

$$\eta_{HCET} = \frac{1}{\sqrt{2}}\left[\left(G_{XH^{\bullet -}} - G_{XH} - (G_{X^+} - G_{XH})\right) - \left(G_{YH^{\bullet -}} - G_{YH} - (G_{Y^+} - G_{YH})\right)\right] \quad \text{(S4)}$$

$$\eta_{HCET} = \frac{1}{\sqrt{2}}\left[\left(-F \cdot E_{XH}^\circ - \Delta G_{XH}^{H^-}\right) - \left(-F \cdot E_{YH}^\circ - \Delta G_{YH}^{H^-}\right)\right] = -\frac{1}{\sqrt{2}}(F \cdot \Delta E^\circ + \Delta\Delta G^{H^-}), \quad \text{(S5)}$$

where ΔE°/ $\Delta\Delta G^{H^-}$ is the difference of reduction potential/hydrictity between electron acceptor (X–H) and electron donor (Y–H).

Similarly for σ:

$$\sigma_{HCET} = \frac{1}{\sqrt{2}}[(\Delta G_{XH}^{e^-} + \Delta G_{XH}^{H^-}) - (\Delta G_{YH}^{e^-} + \Delta G_{YH}^{H^-})] \quad \text{(S6)}$$

$$\sigma_{HCET} = \frac{1}{\sqrt{2}}\left[\left(G_{XH^{\bullet -}} - G_{XH} + (G_{X^+} - G_{XH})\right) - \left(G_{YH^{\bullet -}} - G_{YH} + (G_{Y^+} - G_{YH})\right)\right] \quad \text{(S7)}$$

$$\sigma_{HCET} = \frac{1}{\sqrt{2}}\left[\left(-F \cdot E_{XH}^\circ + \Delta G_{XH}^{H^-}\right) - \left(-F \cdot E_{YH}^\circ + \Delta G_{YH}^{H^-}\right)\right] = -\frac{1}{\sqrt{2}}(F \cdot \Delta E^\circ - \Delta\Delta G^{H^-}) \quad \text{(S8)}$$

Off-diagonal contributions for HCET expressed in terms of full reaction cycles

The three-component thermodynamic model presented in the main text is based on the intrinsic thermodynamic properties of individual reactants - the one-electron reduction free energies of X–H and Y–H ($\Delta G^{e^-}_{XH/YH}$) and the free energies of hydride release of X–H and Y–H (hydricities, $\Delta G^{H^-}_{XH/YH}$). Such an approach depicts tug-of-war type competition over electron and hydride between the two species that can act as electron acceptors (and hydride donors at the same time) and gives rise to eqs (6) and (7) in the main text.

As an alternative to the tug-of-war view of HCET, HCET can be also depicted in terms of electron and hydride transfers (ET and HT) between X–H and Y•. In such case, the full reaction cycle for HCET can be expressed in terms of the Gibbs free energies of ET ($\Delta G_{ET,1}$and $\Delta G_{ET,2}$) and HT between the two reactants ($\Delta G_{HT,1}$and $\Delta G_{HT,2}$, see **Scheme S1**).

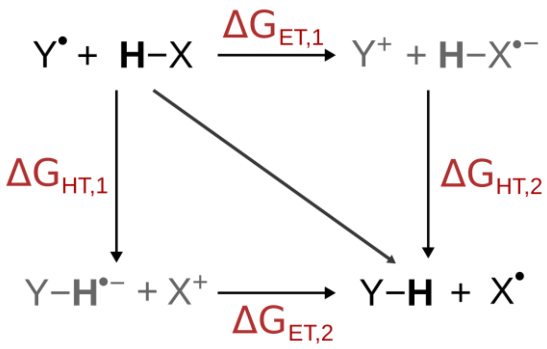

Scheme S1. Full reaction thermodynamic cycle for HCET

Based on the full reaction thermodynamic cycle, asynchronicity can be expressed as $\Delta G_{ET,1} - \Delta G_{HT,1}$:

$$\eta_{HCET} = \frac{1}{\sqrt{2}}(\Delta G_{ET,1} - \Delta G_{HT,1}), \qquad \text{(S9)}$$

where $\Delta G_{ET,1}$ is the difference between the Gibbs free energy of one-electron reduction of X–H ($\Delta G^{e^-}_{XH}$) and the Gibbs free energy of one-electron reduction of Y+ ($\Delta G^{e^-}_{Y+}$ as shown in **Scheme S2**):

$$\Delta G_{ET,1} = \Delta G_{XH}^{e^-} - \Delta G_{Y^+}^{e^-} \quad \text{(S10)}$$

and $\Delta G_{HT,1}$ is the difference between hydricity of X–H ($\Delta G_{XH}^{H^-}$) and hydricity of Y–H$^{\bullet-}$ ($\Delta G_{YH^{\bullet-}}^{H^-}$ as shown in **Scheme S2**):

$$\Delta G_{HT,1} = \Delta G_{XH}^{H^-} - \Delta G_{YH^{\bullet-}}^{H^-} \quad \text{(S11)}$$

Inserting (S10) and (S11) into (S9) yields:

$$\eta_{HCET} = \frac{1}{\sqrt{2}}\left[\left(\Delta G_{XH}^{e^-} - \Delta G_{Y^+}^{e^-}\right) - \left(\Delta G_{XH}^{H^-} - \Delta G_{YH^{\bullet-}}^{H^-}\right)\right], \quad \text{(S12a)}$$

which can be rearranged as:

$$\eta_{HCET} = \frac{1}{\sqrt{2}}\left[\left(\Delta G_{XH}^{e^-} - \Delta G_{XH}^{H^-}\right) - \left(\Delta G_{Y^+}^{e^-} - \Delta G_{YH^{\bullet-}}^{H^-}\right)\right] \quad \text{(S12b)}$$

Eq (S12b) already contains the imbalance between the one-electron reduction and hydride release of/from X–H - $(\Delta G_{XH}^{e-} - \Delta G_{XH}^{H^-})$- as present in eq (6) in main text. The term describing the H-acceptor species, Y$^{\bullet}$: $-\left(\Delta G_{Y^+}^{e-} - \Delta G_{YH^{\bullet-}}^{H^-}\right)$ - can be transformed based on the following relationship derived from the thermodynamic cycle for Y$^{\bullet}$ (**Scheme S2**):

$$\Delta G_{YH}^{e^-} + \Delta G_{YH^{\bullet-}}^{H^-} = \Delta G_{YH}^{H^-} + \Delta G_{Y^+}^{e^-} \quad \text{(S13a)}$$

and consequently:

$$\Delta G_{Y^+}^{e^-} - \Delta G_{YH^{\bullet-}}^{H^-} = \Delta G_{YH}^{e^-} - \Delta G_{YH}^{H^-} \quad \text{(S13b)}$$

Inserting relationship from eq (S13b) into eq (S12b) yields the expression for η_{HCET} introduced in the main text (eq (6)).

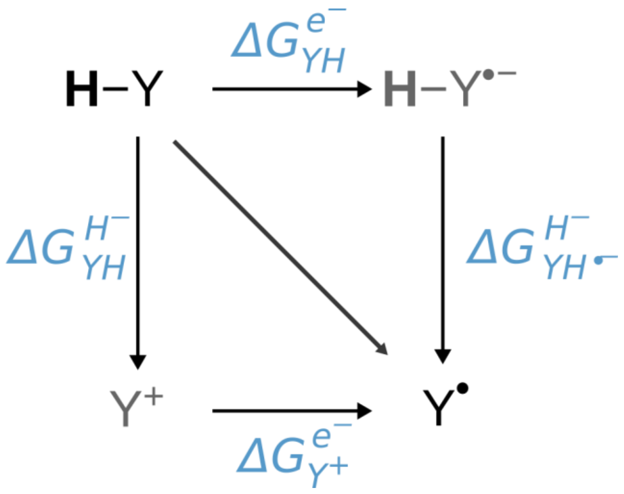

Scheme S2. Half reaction thermodynamic cycle for electron acceptor $Y^{\bullet}$ in HCET.

As a side note, η_{HCET} can be directly expressed through the Gibbs free energies of individual species - eq (S9) can be recast as:

$$\eta_{HCET} = \frac{1}{\sqrt{2}}\left[\left(G_{XH^{\bullet -}} + G_{Y^+} - (G_{XH} + G_{Y^{\bullet}})\right) - \left(G_{X^+} + G_{YH^{\bullet -}} - (G_{XH} + G_{Y^{\bullet}})\right)\right]. \quad \text{(S14)}$$

This expression can be simplified to:

$$\eta_{HCET} = \frac{1}{\sqrt{2}}\left[(G_{XH^{\bullet -}} + G_{Y^+}) - (G_{X^+} + G_{YH^{\bullet -}})\right], \quad \text{(S15)}$$

which corresponds to the difference in Gibbs free energies between the two off-diagonal states, [X–$H^{\bullet -}$ + Y^+] and [Y–$H^{\bullet -}$ + X^+]. Eq (S15) can be similarly obtained from eq (6) in the main text.

Similar to (S9), the remaining off-diagonal term - frustration, based on the full-reaction thermodynamic cycle can be expressed as:

$$\sigma_{1,HCET} = \frac{1}{\sqrt{2}}(\Delta G_{ET,1} + \Delta G_{HT,1}). \quad \text{(S16)}$$

Eq (S16) can be represented in terms of the Gibbs free energies of the individual species as:

$$\sigma_{1,HCET} = \frac{1}{\sqrt{2}}\left[\left(G_{XH^{\bullet -}} + G_{Y^+} - (G_{XH} + G_{Y^\bullet})\right) + \left(G_{X^+} + G_{YH^{\bullet -}} - (G_{XH} + G_{Y^\bullet})\right)\right] \quad \text{(S17)}$$

whereas the original σ_{HCET} from eq (7) in the main text is represented by the Gibbs free energies of the individual species as:

$$\sigma_{HCET} = \frac{1}{\sqrt{2}}\left[\left(G_{XH^{\bullet -}} + G_{X^+} - 2 \cdot G_{XH}\right) - \left(G_{YH^{\bullet -}} + G_{Y^+} - 2 \cdot G_{YH}\right)\right] \quad \text{(S18)}$$

Comparison of eq (S17) with eq (S18) demonstrates that $\sigma_{1,HCET}$ is connected to σ_{HCET} through a term that depends on the thermodynamic properties of Y–H/Y$^\bullet$, and is therefore constant for a series of HAA reactions involving a single H-atom acceptor (Y$^\bullet$) and various H-donors (X–H):

$$\sigma_{1,HCET} - \sigma_{HCET} = \frac{1}{\sqrt{2}}\left(2 \cdot G_{Y^+} - 2 \cdot G_{Y^\bullet} + 2 \cdot G_{YH^{\bullet -}} - 2 \cdot G_{YH}\right), \quad \text{(S19)}$$

which based on **Scheme S2** can be expressed as:

$$\sigma_{1,HCET} = \sigma_{HCET} + \frac{1}{\sqrt{2}}\left(\Delta G_{YH}^{H^-} - \Delta G_{YH^{\bullet -}}^{H^-} + \Delta G_{YH}^{e^-} - \Delta G_{Y^+}^{e^-}\right) \quad \text{(S20)}$$

Alternatively, frustration $\sigma_{2,HCET}$ can be calculated with respect to products of the reaction (X$^\bullet$ and Y–H) as $\frac{1}{\sqrt{2}}(\Delta G_{ET,2} + \Delta G_{HT,2})$:

$$\sigma_{2,HCET} = \frac{1}{\sqrt{2}}\left[\left(G_{XH^{\bullet -}} + G_{Y^+} - (G_{X^\bullet} + G_{YH})\right) + \left(G_{X^+} + G_{YH^{\bullet -}} - (G_{X^\bullet} + G_{YH})\right)\right] \quad \text{(S21)}$$

Comparison of eq (S17) with eq (S21) shows that $\sigma_{2,HCET}$ differs from $\sigma_{1,HCET}$ by the free energy of the reaction:

$$\sigma_{1,HCET} = \sigma_{2,HCET} + \sqrt{2} \times \Delta G_0 \quad \text{(S22)}$$

It must be stressed that η is the same in both formulations (one presented in the main text and one presented here), which is not the case of σ. However, σ_1 from eq (S16) correlates with σ from the main text (eqs (5) and (7)) and thus all conclusions about the joint effect of frustration and asynchronicity on the operative mechanism are independent of the used formulation. Despite this, we favor the formulation presented in the main text as it is associated with the positive range of values for $\Delta G_{00}^{\neq}$, which is expected as it corresponds

to ¼ of the reorganization energy at the synchronous and unfrustrated limit (and the reorganization energy in the Marcus-type model of reactivity is always positive).

Note that conversion between $\eta_{HCET}/\sigma_{HCET}$ expressed in kcal/mol used in this study and $\eta_{HCET}/\sigma_{HCET}$ expressed in Volts involves a change of sign:

$$\eta_{HCET}(\text{kcal mol}^{-1}) = -F\eta_{HCET}(\text{V})$$

and

$$\sigma_{HCET}(\text{kcal mol}^{-1}) = -F\sigma_{HCET}(\text{V})$$

Thus, for instance, both the positive asynchronicity (expressed in Volts) and the negative asynchronicity (expressed in kcal mol^{-1}) equally describe the preference for ET over HT in the HAA reaction.

Off-diagonal contributions to PCET expressed in terms of the reduction potentials and pK$_a$ values of X$^•$ and Y$^•$

The acidity constant ($pK_{a,X^•}$) is the free energy of heterolytic X–H bond dissociation of X–H$^{•+}$ to yield a parent species X$^•$ and the proton H$^+$:

$$RT\ln(10)\cdot pK_a = G_{X^•} - G_{X-H^{•+}} + G_{solv,H^+}, \quad \text{(S23)}$$

where G_{solv,H^+} is the free energy of solvation of a proton (a constant value which cancels out in derivation of η_{PCET} and σ_{PCET}).

The one-electron reduction potential ($E^\circ_{X^•}$) is a quantity reflecting Gibbs free energy change upon 1e$^-$ reduction of a solute (X$^•$):

$$F\cdot E^\circ_{X^•} = G_{X^•} - G_{X^-} - E^\circ_{abs}(\text{reference}) \quad \text{(S24)}$$

Asynchronicity η_{PCET}, defined in Eq (4) in the main text as:

$$\eta_{PCET} = \frac{1}{\sqrt{2}}\left[\left(\Delta G^{e^-}_{Y^•} - \Delta G^{H^+}_{Y^•}\right) - \left(\Delta G^{e^-}_{X^•} - \Delta G^{H^+}_{X^•}\right)\right] \quad \text{(S25)}$$

can be re-expressed using Gibbs free energies of individual species:

$$\eta_{PCET} = \frac{1}{\sqrt{2}}\left[\left(G_{Y^-} - G_{Y^•} - (G_{YH^{•+}} - G_{Y^•})\right) - \left(G_{X^-} - G_{X^•} - (G_{XH^{•+}} - G_{X^•})\right)\right] \quad \text{(S26)}$$

and - based on (S23) and (S24) - transformed to:

$$\eta_{PCET} = \frac{1}{\sqrt{2}}\left[\left(-F\cdot E^\circ_{Y^•} + RT\ln(10)\cdot pK_{a,Y^•}\right) - \left(-F\cdot E^\circ_{X^•} + RT\ln(10)\cdot pK_{a,X^•}\right)\right] = -\frac{1}{\sqrt{2}}\left(F\cdot\Delta E^\circ - RT\ln(10)\cdot\Delta pK_a\right) \quad \text{(S27)}$$

where $\Delta E^\circ/\Delta pK_a$ is the difference of reduction potential/acidity constant between electron (and H-atom) acceptor ($\Delta E^\circ_{Y^•}/pK_{a,Y^•}$) and electron (H-atom) donor ($\Delta E^\circ_{X^•}/pK_{a,X^•}$).

Relatedly, for σ_{PCET}, defined in eq (5) in main text:

$$\sigma_{PCET} = \frac{1}{\sqrt{2}}\left[\left(\Delta G_{Y^\bullet}^{e^-} + \Delta G_{Y^\bullet}^{H^+}\right) - \left(\Delta G_{X^\bullet}^{e^-} + \Delta G_{X^\bullet}^{H^+}\right)\right] \quad (S28)$$

$$\sigma_{PCET} = \frac{1}{\sqrt{2}}\left[\left(G_{Y^-} - G_{Y^\bullet} + (G_{YH^{\bullet+}} - G_{Y^\bullet})\right) - \left(G_{X^-} - G_{X^\bullet} + (G_{XH^{\bullet+}} - G_{X^\bullet})\right)\right] \quad (S29)$$

$$\sigma_{PCET} = \frac{1}{\sqrt{2}}\left[\left(-F \cdot E^\circ_{Y^\bullet} - RT\ln(10) \cdot pK_{a,Y^\bullet}\right) - \left(-F \cdot E^\circ_{X^\bullet} - RT\ln(10) \cdot pK_{a,X^\bullet}\right)\right] = -\frac{1}{\sqrt{2}}\left(F \cdot \Delta E^\circ + RT\ln(10) \cdot \Delta pK_a\right) \quad (S30)$$

Note that conversion between $\eta_{PCET}/\sigma_{PCET}$ expressed in Volts, which is used in refs 29 and 30 in the main text and $\eta_{PCET}/\sigma_{PCET}$ expressed in kcal/mol used in this study involves a change of sign:

$$\eta_{PCET}(\text{kcal mol}^{-1}) = -F\eta_{PCET}(\text{V})$$

and

$$\sigma_{PCET}(\text{kcal mol}^{-1}) = -F\sigma_{PCET}(\text{V})$$

Thus, for instance, both the positive asynchronicity (expressed in Volts) and the negative asynchronicity (expressed in kcal mol^{-1}) equally describe the preference for ET over PT in the HAA reaction.

Off-diagonal contributions for PCET expressed in terms of full reaction cycles

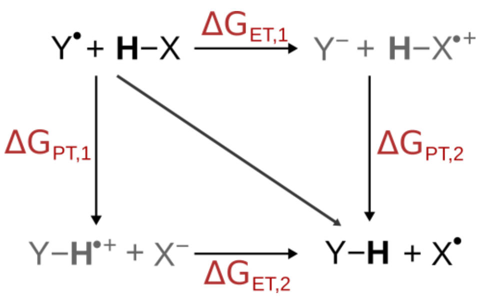

Scheme S3. Full reaction thermodynamic cycle for PCET

Based on the full reaction thermodynamic cycle, asynchronicity η_{PCET} can be expressed as $\Delta G_{ET,1} - \Delta G_{PT,1}$:

$$\eta_{PCET} = \frac{1}{\sqrt{2}}\left(\Delta G_{ET,1} - \Delta G_{PT,1}\right) \quad \text{(S31)}$$

where $\Delta G_{ET,1}$ is the difference between the Gibbs free energy of one-electron reduction of Y$^{\bullet}$ ($\Delta G_{Y^{\bullet}}^{e^-}$) and the Gibbs free energy of one-electron reduction of X–H$^{\bullet+}$ ($\Delta G_{XH^{\bullet+}}^{e^-}$, as shown in **Scheme S4**):

$$\Delta G_{ET,1} = \Delta G_{Y^{\bullet}}^{e^-} - \Delta G_{XH^{\bullet+}}^{e^-} \quad \text{(S32)}$$

and $\Delta G_{PT,1}$is the difference between the Gibbs free energy of protonation of Y$^{\bullet}$ ($\Delta G_{Y^{\bullet}}^{H^+}$) and the Gibbs free energy of protonation of X$^-$ ($\Delta G_{X^-}^{H^+}$, as shown in **Scheme S4**):

$$\Delta G_{PT,1} = \Delta G_{Y^{\bullet}}^{H^+} - \Delta G_{X^-}^{H^+} \quad \text{(S33)}$$

Eqs (S31), (S32) and (S33) yield together:

$$\eta_{PCET} = \frac{1}{\sqrt{2}}\left(\Delta G_{Y^{\bullet}}^{e^-} - \Delta G_{XH^{\bullet+}}^{e^-} - \left(\Delta G_{Y^{\bullet}}^{H^+} - \Delta G_{X^-}^{H^+}\right)\right) \quad \text{(S34)}$$

Eq (S34) can be further converted by using the relationships depicted in **Scheme S4**:

$$\Delta G_{X^\bullet}^{e^-} + \Delta G_{X^-}^{H^+} = \Delta G_{X^\bullet}^{H^+} + \Delta G_{XH^{\bullet+}}^{e^-} \tag{S35a}$$

$$\Delta G_{XH^{\bullet+}}^{e^-} - \Delta G_{X^-}^{H^+} = \Delta G_{X^\bullet}^{e^-} - \Delta G_{X^\bullet}^{H^+} \tag{S35b}$$

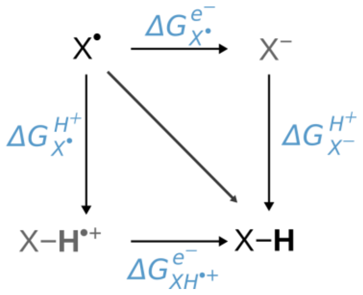

Scheme S4. Half reaction thermodynamic cycle for electron acceptor X$^\bullet$ in PCET.

Inserting the relationship from (S35b) into (S34) yields η_{PCET} as in eq (4) in the main text.

Analogously to η_{HCET}, η_{PCET} can be expressed as the difference in Gibbs free energies between the two off-diagonal states, [X–H$^{\bullet+}$ + Y$^-$] and [Y–H$^{\bullet+}$ + X$^-$] by simplifying (S26) to:

$$\eta_{PCET} = \frac{1}{\sqrt{2}}[(G_{Y^-} + G_{XH^{\bullet+}}) - (G_{X^-} + G_{YH^{\bullet+}})] \tag{S36}$$

Frustration $\sigma_{1,PCET}$, based on the full-reaction thermodynamic cycle, can be expressed as:

$$\sigma_{1,PCET} = \frac{1}{\sqrt{2}}(\Delta G_{ET,1} + \Delta G_{PT,1}) \tag{S37}$$

and hence using the free energies of the individual species:

$$\sigma_{1,PCET} = \frac{1}{\sqrt{2}}\left[\left(G_{XH^{\bullet+}} + G_{Y^-} - (G_{XH} + G_{Y^\bullet})\right) + \left(G_{YH^{\bullet+}} + G_{X^-} - (G_{XH} + G_{Y^\bullet})\right)\right] \tag{S38}$$

whereas σ_{PCET} from eq (5) in the main text is represented by the Gibbs free energies of the individual species as:

$$\sigma_{PCET} = \frac{1}{\sqrt{2}}[(G_{XH^{\bullet+}} + G_{X^-} - 2 \cdot G_{X^\bullet}) - (G_{YH^{\bullet+}} + G_{Y^-} - 2 \cdot G_{Y^\bullet})] \tag{S39}$$

Comparison of (S38) and (S39) leads to:

$$\sigma_{1,PCET} - \sigma_{PCET} = \frac{1}{\sqrt{2}}(2 \cdot G_{Y^-} - 4 \cdot G_{Y^\bullet} + 2 \cdot G_{YH^{\bullet+}} - 2G_{XH} + 2G_{X^\bullet})$$

$$= \frac{1}{\sqrt{2}}\left(2 \cdot G_{Y^-} - 2 \cdot G_{Y^\bullet} + 2 \cdot G_{YH^{\bullet+}} - 2 \cdot G_{YH} + (2 \cdot G_{X^\bullet} - 2 \cdot G_{XH} + 2 \cdot G_{YH} - 2 \cdot G_{Y^\bullet})\right)$$

$$= \frac{1}{\sqrt{2}}\left(\Delta G_{Y^\bullet}^{e^-} - \Delta G_{YH^{\bullet+}}^{e^-} + \Delta G_{Y^\bullet}^{H+} - \Delta G_{Y^-}^{H+} + 2 \cdot \Delta G_0\right), \quad \text{(S40)}$$

which shows that $\sigma_{1,PCET}$ is related to σ_{PCET} via a term related to the thermodynamic properties of Y–H/Y$^\bullet$ (presented in **Scheme S4**, a constant value for a series of HAA reactions involving a single H-atom acceptor (Y$^\bullet$) and various H-donors (X–H)) and the free energy of the reaction ΔG_0.

Alternatively, frustration $\sigma_{2,HCET}$ can be calculated with respect to products of the reaction (X$^\bullet$ and Y–H) as $\frac{1}{\sqrt{2}}(\Delta G_{ET,2} + \Delta G_{PT,2})$:

$$\sigma_{2,PCET} = \frac{1}{\sqrt{2}}\left[\left(G_{XH^{\bullet+}} + G_{Y^-} - (G_{YH} + G_{X^\bullet})\right) + \left(G_{YH^{\bullet+}} + G_{X^-} - (G_{YH} + G_{X^\bullet})\right)\right] \quad \text{(S41)}$$

Comparison of eq (S38) with eq (S41) shows that $\sigma_{2,HCET}$ differs from $\sigma_{1,PCET}$ by the free energy of the reaction:

$$\sigma_{1,PCET} = \sigma_{2,PCET} + \sqrt{2} \times \Delta G_0 \quad \text{(S42)}$$

It must be stressed that η is the same in both formulations (one presented in the main text and one presented here), which is not the case of σ. However, the σ/σ_1/σ_2 values correlate with each other and thus all conclusions about the joint effect of frustration and asynchronicity on the operative mechanism are independent of the used formulation. Despite this, we favor the formulation presented in the main text as it is associated with the positive range of values for $\Delta G_{00}^{\neq}$, which is expected as it corresponds to ¼ of the reorganization energy at the synchronous and unfrustrated limit (and the reorganization energy in the Marcus-type model of reactivity is always positive).

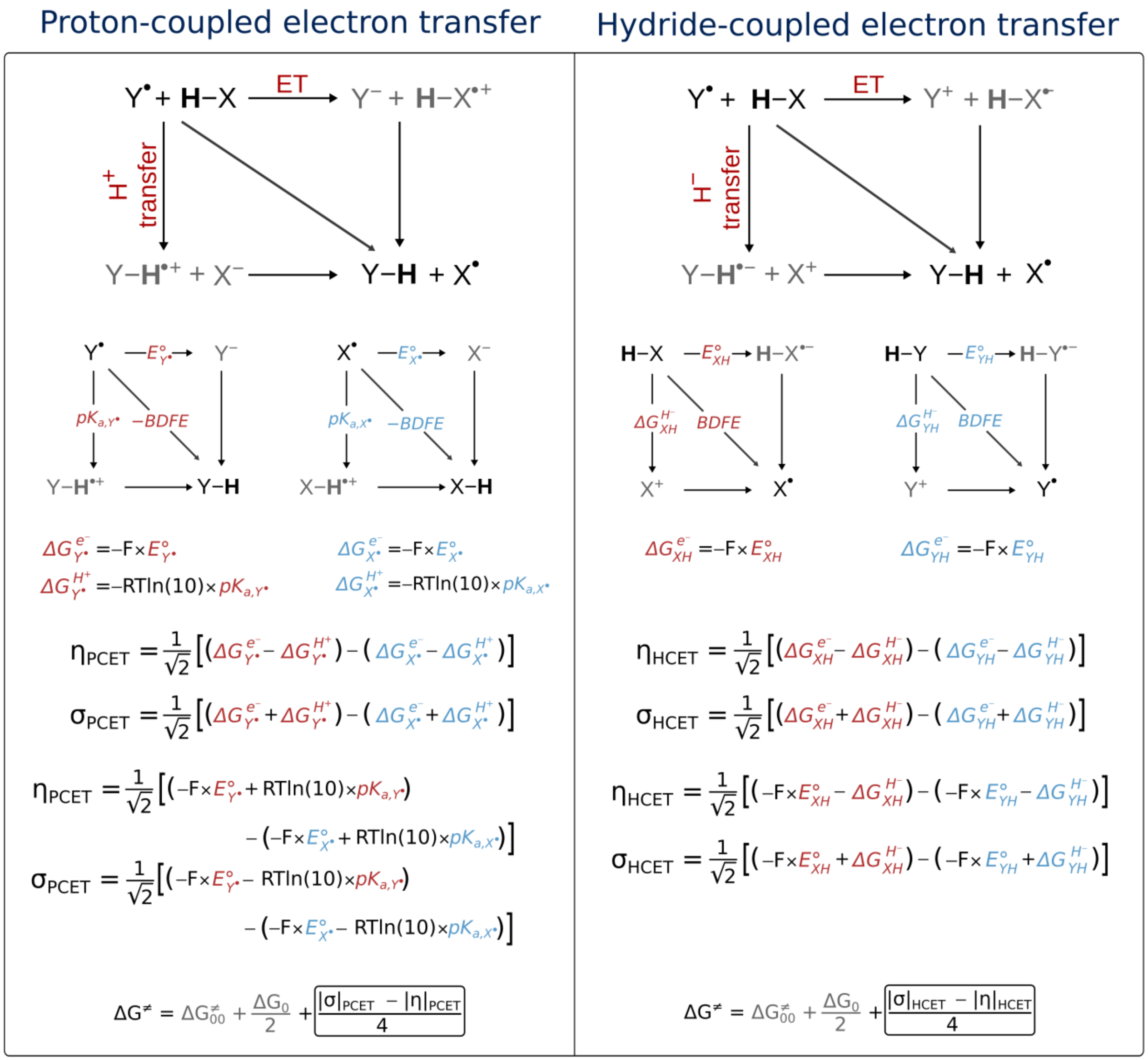
Proton-coupled electron transfer
Hydride-coupled electron transfer
ET
H+ transfer
H− transfer
pKa,Y•
−BDFE
pKa,X•
BDFE
ΔG e− Y• =−F×E°Y•
ΔG H+ Y• =−RTln(10)×pKa,Y•
ΔG e− X• =−F×E°X•
ΔG H+ X• =−RTln(10)×pKa,X•
ΔG e− XH =−F×E°XH
ΔG e− YH =−F×E°YH
ηPCET = 1/√2 [(ΔG e− Y• − ΔG H+ Y•) − (ΔG e− X• − ΔG H+ X•)]
σPCET = 1/√2 [(ΔG e− Y• + ΔG H+ Y•) − (ΔG e− X• + ΔG H+ X•)]
ηPCET = 1/√2 [(−F×E°Y• + RTln(10)×pKa,Y•) − (−F×E°X• + RTln(10)×pKa,X•)]
σPCET = 1/√2 [(−F×E°Y• − RTln(10)×pKa,Y•) − (−F×E°X• − RTln(10)×pKa,X•)]
ηHCET = 1/√2 [(ΔG e− XH − ΔG H− XH) − (ΔG e− YH − ΔG H− YH)]
σHCET = 1/√2 [(ΔG e− XH + ΔG H− XH) − (ΔG e− YH + ΔG H− YH)]
ηHCET = 1/√2 [(−F×E°XH − ΔG H− XH) − (−F×E°YH − ΔG H− YH)]
σHCET = 1/√2 [(−F×E°XH + ΔG H− XH) − (−F×E°YH + ΔG H− YH)]
ΔG≠ = ΔG≠00 + ΔG0/2 + (|σ|PCET − |η|PCET)/4
ΔG≠ = ΔG≠00 + ΔG0/2 + (|σ|HCET − |η|HCET)/4

Figure S1. The full-reaction thermodynamic cycle for PCET (*left*) and HCET (*right*) between the H-atom donor X–H and the H-atom abstractor Y•. Each of the full-reaction cycles are deconstructed into respective two half-reaction thermodynamic blocks: one for electron accepting species Y•/X–H (red arrows) and one for electron donating one X•/Y–H (blue arrows) involving key energy parameters: the free energy of one-electron reduction ($\Delta G^{e^-} = -F \times E^{\circ}$) and the free energy of protonation ($\Delta G^{H^+} = -RT\ln(10) \times pK_a$) for PCET, and the analogous free energy of one-electron reduction and the free energy of hydride release (hydricity, ΔG^{H^-}) for HCET. The equations demonstrate how E°, pK_a and ΔG^{H^-} are explicitly incorporated into the calculation of σ_{PCET}, η_{PCET} and σ_{HCET}, η_{HCET}.

Table S1. Gibbs free energies of investigated species obtained at the B3LYP-D3/def2-SVP/CPCM level of theory. The values are given in Hartree.

	G(subH)	G(catrad)	G(anion)	G(anionrad)	G(cation)	G(rad)
cyclohexane	-235.5870800	-235.3097579	-234.9897959	-235.5590716	-234.760616	-234.946117
cyclohexene	-234.3882162	-234.1454694	-233.8301481	-234.3908542	-233.5763406	-233.7625748
DHA	-540.2306420	-539.9973934	-539.712297	-540.2628994	-539.4399158	-539.614711
fluorene	-500.9662308	-500.7425842	-500.4633711	-501.0156151	-500.1438169	-500.3425738
Ph_2CH_2	-502.1414053	-501.9012635	-501.6186285	-502.1728803	-501.3320865	-501.5153143
THF	-232.2104178	-231.9502539	-231.62241	-232.177506	-231.4114686	-231.5665919
toluene	-271.3042935	-271.0554534	-270.7553262	-271.3232365	-270.4655593	-270.6637219
2,7-di(NMe_2) -fluorene	-768.6103731	-768.4387445	-768.0929585	-768.6392553	-767.7973427	-767.9841398
oxetane	-192.9099395	-192.6565777	-192.3316157	-192.8834777	-192.1085083	-192.264387
1,3-CHD	-233.1824648	-232.9655494	-232.6477766	-233.2228734	-232.3938172	-232.571178
CHD	-233.1691663	-232.9438527	-232.643756	-233.1813552	-232.3889642	-232.5578969
CF_3-Phe	-643.9984842	-643.7502208	-643.516374	-644.0362144	-643.1019103	-643.3617475
Cl-Phe	-766.6812883	-766.4502034	-766.1931763	-766.7155627	-765.8127809	-766.0510598

H-Phe	-307.2000097	-306.9664036	-306.7068993	-307.2229195	-306.3239015	-306.5683813
Me-Phe	-346.4676865	-346.2460462	-345.974818	-346.4908429	-345.6111161	-345.8398691
NMe_2-Phe	-441.0173438	-440.8344451	-440.5164912	-441.0367981	-440.2166358	-440.4049291
NO_2-Phe	-511.5610241	-511.3047177	-511.0919367	-511.6530736	-510.6559101	-510.9214043
OMe-Phe	-421.6142306	-421.4044263	-421.1173547	-421.639295	-420.7803448	-420.9921429
TEMPOH	-483.7983698	-483.6085652	-483.2765043	-483.8632395	-482.9561975	-483.1950739
	G(Cu(II)-OH_2)	G(Cu(III)-OH_2)	G(Cu(II)-OH)	G(Cu(I)-OH_2)	G(Cu(IV)-OH)	G(Cu(III)-OH)
CuIII-OH	-3233.659033	-3233.446508	-3233.186894	-3233.774325	-3232.795173	-3233.020498
	G(Cu(I)-OH_2)	G(Cu(II)-OH_2)	G(Cu(I)-OH)	G(Cu(0)-OH_2)	G(Cu(III)-OH)	G(Cu(II)-OH)
CuII-OH	-3233.774325	-3233.659033	-3233.261305	-3233.825662	-3233.020498	-3233.186894
	G([L^{3-}Ni(II)OH_2]$^{-}$)	G([$L^{\bullet 2-}$ Ni(II)OH_2])	G([L^{3-}Ni(II)OH]$^{2-}$)	G([L^{3-}Ni(I)OH_2]$^{2-}$)	G([$L^{\bullet 2-}$Ni(III)OH])	G([$L^{\bullet 2-}$Ni(II)OH]$^{-}$)
[LNi(II)OH]$^{-}$	-2863.030975	-2862.892238	-2862.558875	-2863.11634	-2862.277165	-2862.437789
	G([$L^{\bullet 2-}$Ni(II)OH_2])	G([L^{-}Ni(II)OH_2]$^{+}$)	G([$L^{\bullet 2-}$Ni(II)OH]$^{-}$)	G([L^{3-}Ni(II)OH_2]$^{-}$)	G([LNi(II)OH]$^{+}$)	G([L^{-}Ni(II)OH])
[LNi(II)OH]	-2862.892194	-2862.718376	-2862.437787	-2863.029145	-2862.086608	-2862.278816

Table S2. Thermodynamic data for the studied substrates and H-atom acceptors. The values were obtained at the B3LYP(D3)/def2-SVP level, with the implicit CPCM solvation model of THF (DMF for TEMPOH/Ni^{II}–OH systems) at 298 K. All energies are given in kcal mol^{-1}.

**substrates**	ΔG_0 (half-reaction)	ω_{H^+}	μ_{H^+}	ω_{H^-}	μ_{H^-}
cyclohexane	-402.21	141.97	-180.73	-354.29	379.14
cyclohexene	-392.60	139.91	-199.88	-361.41	359.07
DHA	-386.50	126.50	-213.10	-365.17	336.55

fluorene	-391.35	123.89	-231.09	-386.83	343.01
Ph_2CH_2	-392.88	125.41	-217.09	-373.07	345.14
THF	-404.01	145.47	-195.00	-339.90	369.11
toluene	-401.97	133.17	-214.46	-380.57	363.75
2,7-di(NMe_2)fluorene	-392.97	153.43	-250.00	-373.57	347.95
oxetane	-405.09	144.19	-203.85	-343.87	367.35
1,3-CHD	-383.59	141.00	-208.98	-367.87	332.01
CHD	-383.58	133.16	-209.35	-351.60	340.78
CF_3-Phe	-399.56	103.76	-240.98	-414.57	381.08
Cl-Phe	-395.47	114.05	-240.17	-400.58	370.16
H-Phe	-396.35	115.15	-238.07	-398.91	378.58
Me-Phe	-393.96	120.35	-240.11	-390.35	369.80
NMe_2-Phe	-384.30	141.08	-240.09	-363.92	346.66
NO_2-Phe	-401.37	94.41	-245.75	-442.46	360.77
OMe-Phe	-390.37	127.38	-238.50	-381.13	358.89
TEMPOH	-378.57	147.34	-219.60	-402.47	344.90
**H-atom acceptors**					
CuIII-OH	-400.69	115.19	-262.86	-434.47	332.15
CuII-OH	-368.62	176.48	-242.51	-357.26	311.71
$[LNi(II)OH]^-$	-372.23	147.92	-255.37	-372.36	296.60
[LNi(II)OH]	-384.90	124.50	-265.58	-418.22	296.68

Table S3. Reactivity data for the **Cu(III)-OH/**substrate set (singlet). The values were obtained at the B3LYP(D3)/def2-SVP level, with the implicit CPCM solvation model of THF at 298 K. All energies are given in kcal mol^{-1}.

**substrates**	ΔG_0	$\Delta G^{\neq}$	η_{H^+}	σ_{H^+}	$\Delta G_{offdiag}^{H^+}$	$\Delta G_{thermo}^{H^+}$	η_{H^-}	σ_{H^-}	$\Delta G_{offdiag}^{H^-}$	$\Delta G_{thermo}^{H^-}$
cyclohexane	1.52	13.40	-26.78	-82.13	13.84	14.60	80.18	46.99	-8.30	-7.54
cyclohexene	-8.09	7.82	-24.72	-62.98	9.57	5.52	73.05	26.92	-11.53	-15.58
DHA	-14.18	0.74	-11.31	-49.76	9.61	2.52	69.29	4.39	-16.23	-23.32
fluorene	-9.34	5.25	-8.70	-31.77	5.77	1.10	47.63	10.85	-9.20	-13.86
Ph_2CH_2	-7.81	6.18	-10.21	-45.77	8.89	4.98	61.39	12.99	-12.10	-16.01
THF	3.32	10.41	-30.27	-67.86	9.40	11.06	94.56	36.96	-14.40	-12.74
toluene	1.28	14.34	-17.98	-48.40	7.60	8.24	53.90	31.60	-5.57	-4.94
CF_3-Phe	-1.13	-9.24	11.43	-21.88	2.61	2.05	19.90	48.93	7.26	6.69
Cl-Phe	-5.21	-9.5	1.15	-22.69	5.39	2.78	33.89	38.01	1.03	-1.58
H-Phe	-4.33	-8.59	0.05	-24.79	6.18	4.02	35.56	46.43	2.72	0.55
Me-Phe	-6.73	-7.08	-5.15	-22.75	4.40	1.04	44.12	37.65	-1.62	-4.98
NMe_2-Phe	-16.39	---	-25.89	-22.77	-0.78	-8.97	70.55	14.50	-14.01	-22.21
NO_2-Phe	0.68	-10.04	20.78	-17.11	-0.92	-0.58	-7.99	28.62	5.16	5.50
OMe-Phe	-10.32	-5.63	-12.18	-24.36	3.05	-2.12	53.34	26.73	-6.65	-11.81

Table S4. Reactivity data for the **Cu(III)-OH/**substrate set (singlet). The values were obtained at the B3LYP(D3)/def2-TZVP level, with the implicit CPCM solvation model of THF at 298 K. All energies are given in kcal mol^{-1}.

	ΔG_0	η_{H^+}	σ_{H^+}	$\Delta G^{H^+}_{offdiag}$	ΔG^{H+}_{thermo}	η_{H^-}	σ_{H^-}	$\Delta G^{H^-}_{offdiag}$	$\Delta G^{H^-}_{thermo}$
cyclohexane	5.49	-26.11	-73.28	11.79	14.54	72.01	40.46	-7.89	-5.14
cyclohexene	-3.94	-25.00	-56.75	7.94	5.97	68.62	24.79	-10.96	-12.93
DHA	-9.68	-13.37	-45.54	8.04	3.21	66.49	4.47	-15.50	-20.34
fluorene	-5.04	-11.62	-27.48	3.97	1.45	45.75	11.33	-8.61	-11.13
Ph_2CH_2	-3.55	-12.63	-41.53	7.22	5.45	58.81	12.97	-11.46	-13.23
THF	7.97	-27.22	-59.81	8.15	12.13	85.36	31.67	-13.42	-9.44
toluene	5.14	-18.73	-42.80	6.02	8.59	50.49	30.26	-5.06	-2.49

Table S5. Reactivity data for the **Cu(II)-OH**/substrate set (doublet). The values were obtained at the B3LYP(D3)/def2-SVP level, with the implicit CPCM solvation model of THF at 298 K. All energies are given in kcal mol^{-1}.

**substrates**	ΔG_0	$\Delta G^{\neq}$	η_{H+}	σ_{H+}	$\Delta G^{H^+}_{offdiag}$	$\Delta G^{H^+}_{thermo}$	η_{H-}	σ_{H-}	$\Delta G^{H^-}_{offdiag}$	$\Delta G^{H^-}_{thermo}$
cyclohexane	33.59	34.79	34.51	-61.78	6.82	23.61	2.98	67.44	16.12	32.91
cyclohexene	23.98	23.55	36.57	-42.63	1.52	13.51	-4.15	47.37	10.80	22.80
DHA	17.88	8.72*	49.98	-29.41	-5.14	3.80	-7.91	24.84	4.23	13.17
fluorene	22.73	5.52	52.59	-11.42	-10.29	1.07	-29.57	31.30	0.43	11.80
Ph_2CH_2	24.26	---	51.07	-25.42	-6.41	5.72	-15.81	33.44	4.41	16.53
THF	35.39	32.98	31.01	-47.51	4.12	21.82	17.36	57.40	10.01	27.71
toluene	33.35	---	43.31	-28.05	-3.81	12.86	-23.30	52.05	7.19	23.86
2,7-di(NMe_2)fluorene	24.35	8.05	23.05	7.49	-3.89	8.28	-16.31	36.23	4.98	17.16
oxetane	36.47	31.47	32.29	-38.66	1.59	19.83	13.40	55.64	10.56	28.80
1,3-CHD	14.97	16.73	35.48	-33.54	-0.49	7.00	-10.60	20.30	2.42	9.91

CHD	14.96	8.64	43.32	-33.16	-2.54	4.94	5.67	29.07	5.85	13.33

*an estimate based on unfinished TS optimization

Table S6. Reactivity data for the **Ni(II)-OH**/complexes. The values were obtained at the B3LYP(D3)/def2-SVP level, with the implicit CPCM solvation of DMF at 298 K. All energies are given in kcal mol $^{-1}$. The values for the $\mathrm{H}_{\rightarrow}/\mathrm{e}_{\leftarrow}$ cycle are calculated using S=1 $TEMPO^{+}$ as the off-diagonal state in line with the mechanism observed in IBO analysis; values in parentheses are calculated using the ground state S=0 $TEMPO^{+}$.

**system**	ΔG_0	$\Delta G^{\neq}$	η_{H^+}	σ_{H^+}	$\Delta G^{H^+}_{offdiag}$	$\Delta G^{H^+}_{thermo}$	η_{H^-}	σ_{H^-}	$\Delta G^{H^-}_{offdiag}$	$\Delta G^{H^-}_{thermo}$
TEMPOH / $[LNi(II)OH]^-$	6.34	-1.47	0.58	-35.77	**8.80**	11.97	-30.11 (-6.10)	48.30 (24.29)	**4.55 (4.55)**	7.72 (7.72)
TEMPOH / [LNi(II)OH] S=0	-6.33	-6.85	-22.84	-45.97	**5.78**	2.62	14.86 (39.76)	47.32 (24.21)	**8.12 (-3.89)**	4.95 (-7.05)
TEMPOH / [LNi(II)OH] S=1	-11.65	-8.79	-28.03	-48.31	**5.07**	-.075	15.75 (38.86)	48.22 (23.31)	**8.12** (-3.89)	2.29 (-9.71)

Table S7. Charges and volumes (at isodensity surface 0.002) of the H-atom, H-atom donor (substrate) and H-atom acceptor calculated for stationary points (RC and TS) obtained for HAAs from the the C–H substrates by the **Cu(III)-OH** complex.

**TS**						
	q (*e*)			*V* (a.u.^{3})		
**substrates**	H	substrate	H-atom acceptor	H	substrate	H-atom acceptor
cyclohexane	0.295	0.062	-0.360	19.4	761.0	3879.6
cyclohexene	0.288	0.075	-0.366	20.6	713.8	3890.3
DHA	0.303	-0.018	-0.326	20.0	1354.3	3894.8
fluorene	0.363	-0.086	-0.279	18.0	1236.1	3895.6

Ph_2CH_2	0.322	0.010	-0.335	19.0	1307.1	3888.7
THF	0.279	0.110	-0.392	20.5	561.2	3890.1
toluene	0.343	0.024	-0.369	18.9	753.4	3891.5
**RC**						
	H	substrate	H-atom acceptor	H	substrate	H-atom acceptor
cyclohexane	-0.037	0.018	0.018	42.4	759.9	3887.1
cyclohexene	0.008	-0.020	0.010	38.8	717.0	3881.3
DHA	0.047	-0.056	0.008	35.2	1355.6	3889.7
fluorene	0.033	-0.016	-0.018	39.1	1231.4	3898.6
Ph_2CH_2	0.037	-0.047	0.006	36.3	1318.3	3886.7
THF	0.039	-0.052	0.012	36.7	563.5	3867.7
toluene	0.014	-0.006	-0.009	40.4	756.0	3891.8

Table S8. Charges and volumes (at isodensity surface 0.002) of the H-atom, H-atom donor (substrate) and H-atom acceptor calculated for stationary points (RC and TS) obtained for HAAs from the the C–H substrates by the **Cu(II)-OH** complex.

**TS**						
	q (*e*)			*V* (a.u.^{3})		
**substrates**	H	substrate	H-atom acceptor	H	Substrate	H-atom acceptor
cyclohexane	0.538	-0.132	-1.406	14.0	772.8	3922.1
cyclohexene	0.558	-0.336	-1.224	13.8	736.5	3929.7
DHA	0.553	-0.700	-0.855	14.1	1380.6	3912.2
fluorene	0.472	-0.640	-0.835	15.7	1261.2	3916.8

THF	0.536	-0.092	-1.446	14.2	571.9	3925.4
2,7-di(NMe_2)fluorene	0.512	-0.668	-0.846	15.0	1955.4	3916.3
oxetane	0.556	-0.107	-1.449	13.9	451.7	3922.7
1,3-CHD	0.516	-0.543	-0.974	15.1	702.8	3938.0
CHD	0.557	-0.534	-1.025	14.2	703.3	3935.2
**RC**						
	H	substrate	H-atom acceptor	H	Substrate	H-atom acceptor
cyclohexane	-0.008	-0.031	-0.964	36.0	760.3	3928.2
cyclohexene	0.042	-0.064	-0.979	34.7	717.3	3931.4
DHA	0.080	-0.110	-0.970	34.2	1357.4	3937.5
fluorene	0.118	-0.167	-0.973	30.5	1231.9	3928.4
THF	0.029	-0.053	-1.073	35.0	564.4	3936.1
2,7-di(NMe_2)fluorene	0.085	-0.114	-0.975	32.6	1925.5	3931.3
oxetane	0.023	-0.051	-0.975	35.6	444.1	3913.5
1,3-CHD	0.051	-0.062	-0.990	35.4	672.2	3935.3
CHD	-0.032	0.025	-0.994	40.9	668.8	3937.1

Table S9. Charges and volumes (at isodensity surface 0.002) of the H-atom, H-atom donor (substrate) and H-atom acceptor calculated for stationary points (TS) obtained for HAAs from the the para-substituted phenols by the **Cu(III)-OH** complex.

**TS**						
	q (*e*)			*V* (a.u.^{3})		
**substrates**	H	substrate	H-atom acceptor	H	substrate	H-atom acceptor
CF_3-Phe	0.653	-0.527	0.444	8.70	904.51	3736.23

Cl-Phe	0.655	-0.769	0.689	8.77	824.07	3726.82
H-Phe	0.661	-0.457	0.414	8.54	684.91	3738.85
Me-Phe	0.668	-0.431	0.379	8.31	820.75	3745.24
NMe_2-Phe	---	---	---	---	---	---
NO_2-Phe	0.654	-0.648	0.620	8.73	861.46	3732.89
OMe-Phe	0.660	-0.312	0.271	9.15	871.45	3749.74

Table S10. Charges and volumes (at isodensity surface 0.002) of the H-atom, H-atom donor (substrate) and H-atom acceptor calculated for stationary points (TS) obtained for HAA reactions between **Ni(II)-OH** and TEMPOH.

**TS**						
	q (*e*)			*V* (a.u.^{3})		
	H	substrate	H-atom acceptor	H	substrate	H-atom acceptor
TEMPOH/ $[LNi(II)OH]^-$	0.630	-0.436	-1.197	9.01	1292.840	3074.640
TEMPOH/ [LNi(II)OH]	0.638	-0.270	-0.370	9.01	1287.029	3058.395

Table S11. Charges and volumes of the H-atom and the donor/acceptor in self-exchange reactions

	q (*e*)		*V* (a.u.^{3})	
**substrates**	H	donor/acceptor	H	donor/acceptor
cyclohexane	0.111	-0.055	25.0	752.8
cyclohexene	0.109	-0.054	25.5	707.3
DHA	0.123	-0.061	24.6	1342.1
fluorene	0.129	-0.065	24.1	1215.1
Ph_2CH_2	0.118	-0.067	23.7	1296.4

THF	0.131	-0.066	25.4	559.5
toluene	0.124	-0.062	25.3	745.0
2,7-di(NMe_2)fluorene	0.134	-0.067	24.3	1907.7
oxetane	0.143	-0.076	24.8	436.4
1,3-CHD	0.103	-0.051	24.1	662.7
CHD	0.118	-0.059	24.9	662.3
CF_3-Phe	0.650	-0.325	8.565	897.7
Cl-Phe	0.648	-0.324	8.583	799.6
H-Phe	0.648	-0.324	8.575	680.7
Me-Phe	0.647	-0.323	8.590	809.6
NO_2-Phe	0.653	-0.326	8.573	848.4
OMe-Phe	0.646	-0.323	8.600	869.1
TEMPOH	0.608	-0.304	9.735	1294.5
Cu(III)-OH / Cu(II)-OH	0.647	-0.385/-0.243	8.892	1462.1/1457.0
Cu(II)-OH / Cu(I)-OH	0.637	-1.320	9.157	1511.1
$[L^{\bullet 2-}Ni(II)OH]^{-}$ / $[L^{3-}Ni(II)OH_2]^{-}$	0.641	-1.320	9.087	2305.6
$[L^{-}Ni(II)OH]$ / $[L^{\bullet 2-}Ni(II)OH_2]$	0.634	-0.317	8.428	2265.7

Table S12. Change of charge, Δ*q*, and volume, Δ*V*, on the H-atom, the substrate fragment and the H-atom acceptor upon RC-to-TS transition during the HAA reactions with **Cu(III)-OH** and **Cu(II)-OH**. For Δ*q* and Δ*V*, see eqs (S43) and (S44).

	Δ*q* (*e*)			Δ*V* (a.u.^{3})		
**Cu(III)-OH**	H	substrate	H-atom acceptor	H	substrate	H-atom acceptor
cyclohexane	0.332	0.044	-0.378	-23.0	1.1	-7.5

cyclohexene	0.279	0.095	-0.376	-18.2	-3.2	9.0
DHA	0.255	0.038	-0.334	-15.2	-1.3	5.1
fluorene	0.330	-0.070	-0.261	-21.1	4.7	-3.0
Ph2CH2	0.285	0.057	-0.341	-17.3	-11.1	2.0
THF	0.240	0.162	-0.403	-16.2	-2.3	22.4
toluene	0.329	0.030	-0.360	-21.6	-2.5	-0.3
**Cu(II)-OH**	H	substrate	H-atom acceptor	H	substrate	H-atom acceptor
cyclohexane	0.546	-0.101	-0.442	-22.1	12.5	-6.1
cyclohexene	0.516	-0.272	-0.246	-20.9	19.1	-1.6
DHA	0.473	-0.590	0.115	-20.0	23.3	-25.4
fluorene	0.355	-0.473	0.138	-14.7	29.3	-11.6
THF	0.507	-0.039	-0.373	-20.8	7.5	-10.8
2,7-di(NMe_2)fluorene	0.427	-0.554	0.130	-17.7	29.9	-14.9
oxetane	0.533	-0.057	-0.474	-21.7	7.6	9.2
1,3-CHD	0.465	-0.481	0.016	-20.3	30.7	2.8
CHD	0.589	-0.559	-0.031	-26.8	34.5	-1.9

Table S13. Change of charge, Δ*q*, and volume, Δ*V*, on H atom, calculated with reference to the average of charge and volume on H-atom in two respective self-exchange reactions, for the **Cu(III)-OH**, **Cu(II)-OH** and **Ni(II)-OH** systems. For Δ*q* and Δ*V*, see eqs (8) and (9) in the main text.

**Cu(III)-OH**	Δ*q* @ H	Δ*V* @ H	**Cu(II)-OH**	Δ*q* @ H	Δ*V* @ H	**Ni(II)-OH**	Δ*q* @ H	Δ*V* @ H
cyclohexane	-0.084	2.5	Cyclohexane	0.164	-3.1	TEMPOH/ $[LNi(II)OH]^-$	0.005	-0.4
cyclohexene	-0.091	3.4	Cyclohexene	0.184	-3.5	TEMPOH/ [LNi(II)OH]	0.018	-0.07
DHA	-0.083	3.3	DHA	0.173	-2.7			
fluorene	-0.025	1.5	Fluorene	0.089	-0.9			
Ph_2CH_2	-0.061	2.7	THF	0.151	-3.1			
THF	-0.110	3.3	2,7-di(NMe_2)fluorene	0.126	-1.8			
toluene	-0.043	1.8	Oxetane	0.166	-3.1			
CF_3-Phe	0.004	-0.031	1,3-CHD	0.146	-1.5			
Cl-Phe	0.007	0.034	CHD	0.179	-2.8			
H-Phe	0.014	-0.191						
Me-Phe	0.021	-0.427						
NO_2-Phe	0.004	-0.002						
OMe-Phe	0.013	0.408						

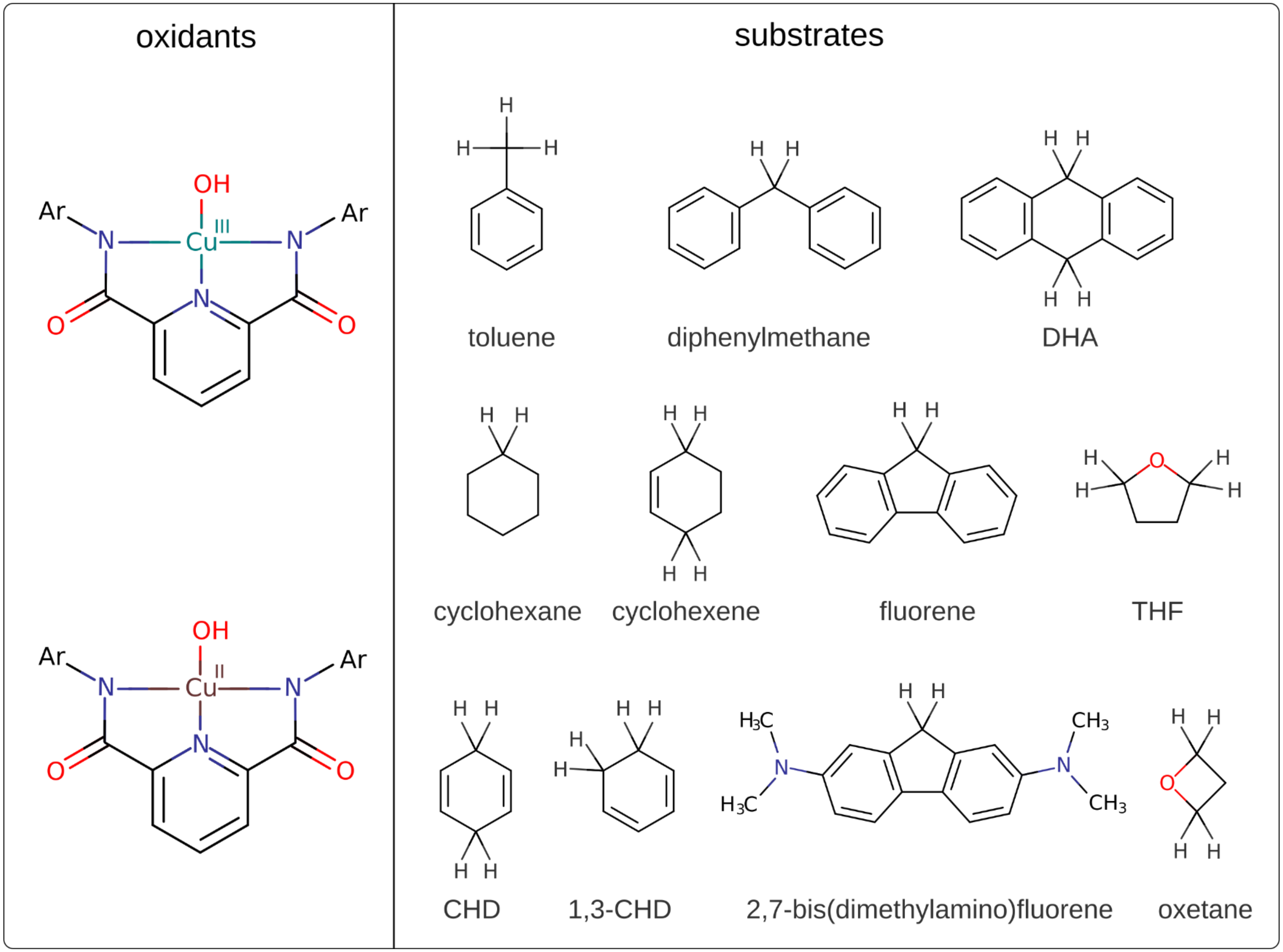

Figure S2. Oxidants and substrates used in the study.

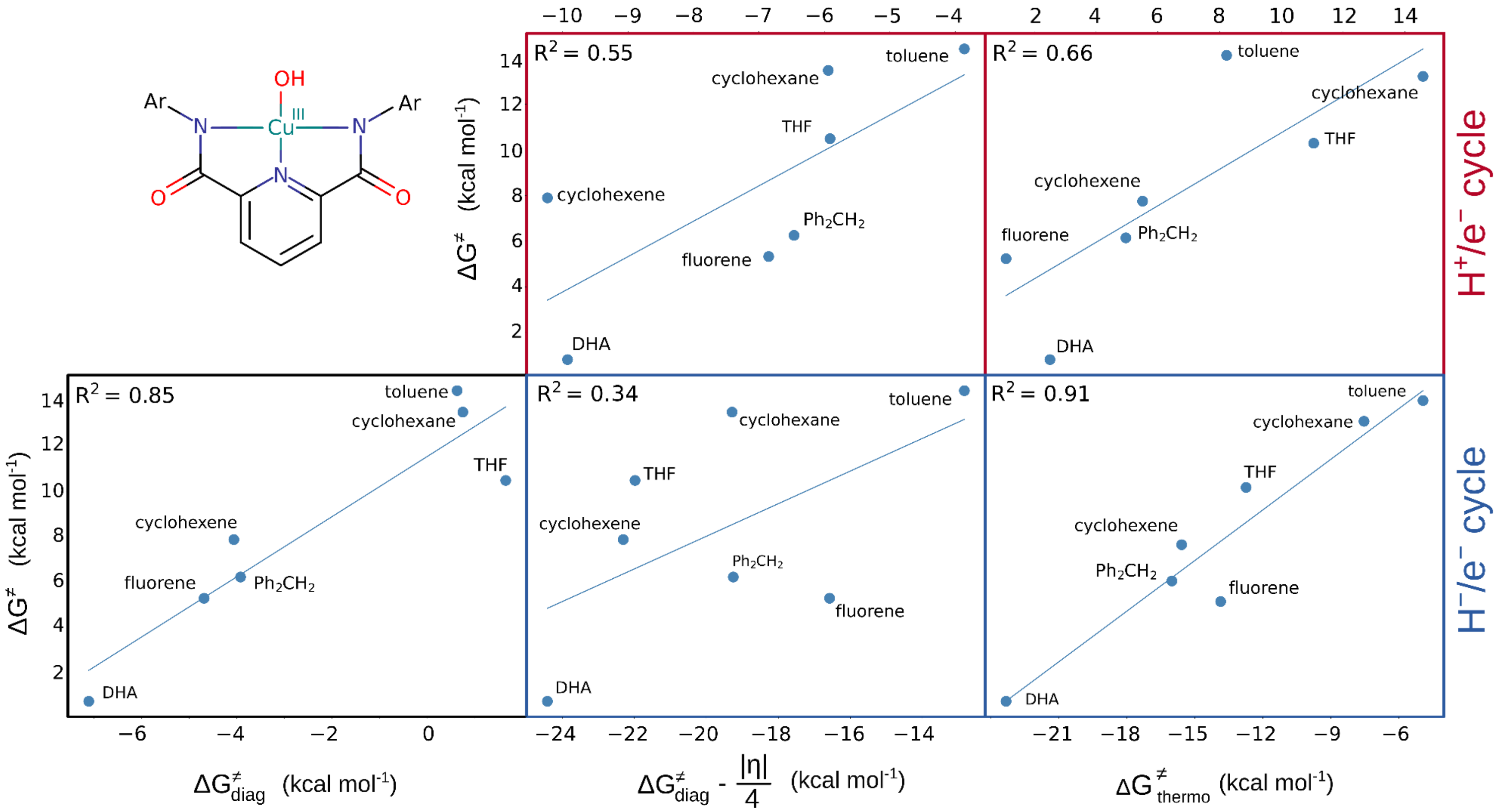

Figure S3. Three-component thermodynamics model applied to the **Cu(III)–OH**-based set of reactions, $\Delta G^{\neq}$ vs: linear free energy relationship LFER (left); LFER with the effect of asynchronicity (middle); LFER together with the complete off-diagonal term (right) using the descriptors derived based on the $H^{+}_{\rightarrow}/e^{-}_{\rightarrow}$ cycle (top, mismatched model featuring high off-diagonal contributions) and $H^{-}_{\rightarrow}/e^{-}_{\leftarrow}$ cycle (bottom, model featuring lower off-diagonal contributions). The quality of correlations is assessed by the squared Pearson's coefficient (R^2).

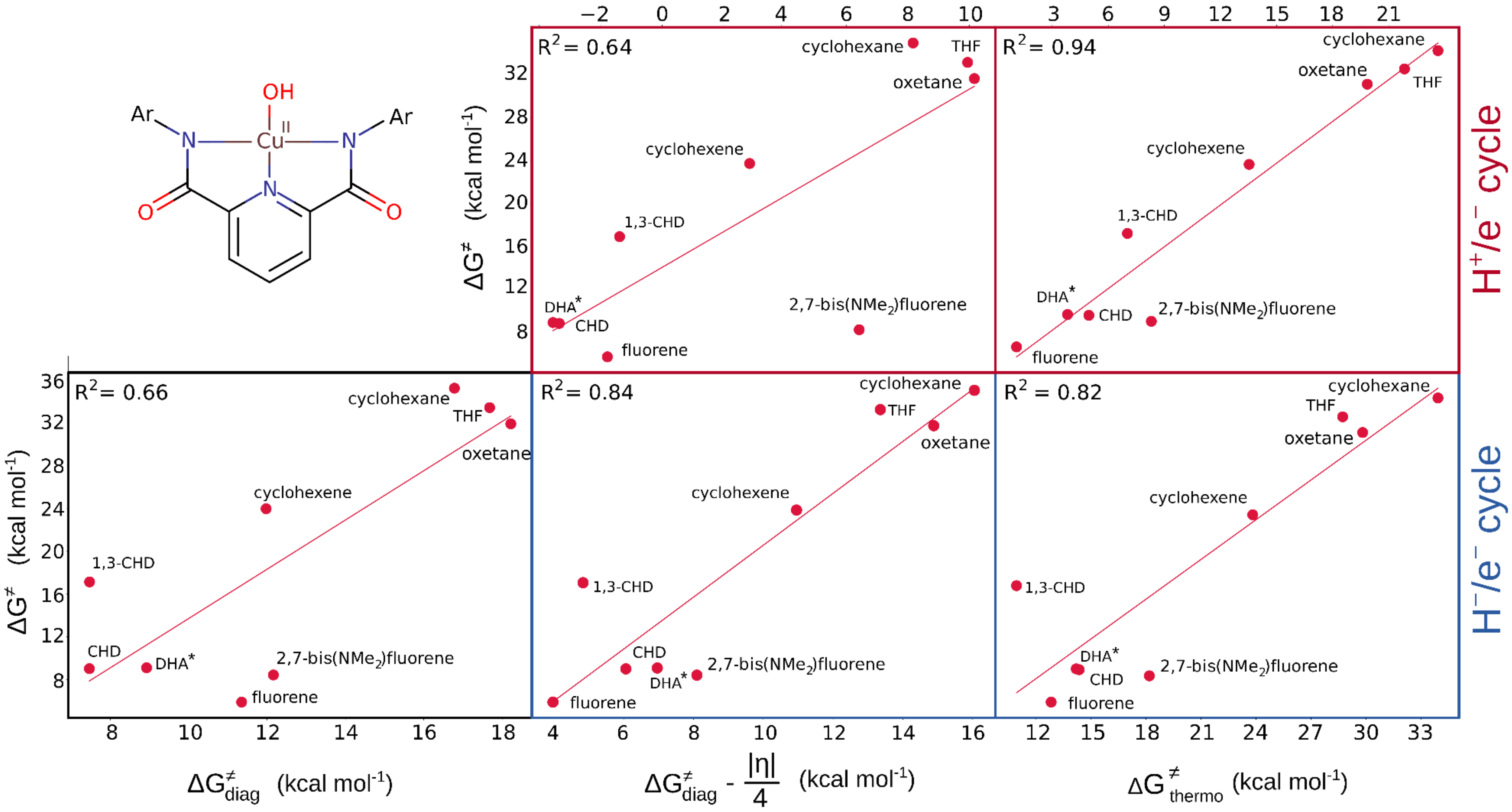

Figure S4. Three-component thermodynamics model applied to the **Cu(II)–OH**-based set of reactions, $\Delta G^{\neq}$ vs: linear free energy relationship LFER (left); LFER with the effect of asynchronicity (middle); LFER together with the complete off-diagonal term (right) using the descriptors derived based on the $H^{+}_{\rightarrow}/e^{-}_{\rightarrow}$ cycle (top, model featuring lower off-diagonal contributions) and $H^{-}_{\rightarrow}/e^{-}_{\leftarrow}$ cycle (bottom, mismatched model featuring high off-diagonal contributions). The quality of correlations is assessed by the squared Pearson's coefficient (R^2).

Analysis of the reactions based on electronic-structure descriptors

In previous work,[31] we successfully correlated the changes in AIM charge and volume of the hydroxyl group in going from the reactant complex (RC) to the transition state, providing insights into the mechanism of hydroxyl group transfer reactions. In the full analogy, we can also introduce for the presented HAA reactions the following descriptors:

$$\Delta q_{\mathrm{RC}\rightarrow\mathrm{TS}} = q_{sub/Cu}^{TS} - q_{sub/Cu}^{RC} \qquad \text{(S43)}$$

$$\Delta V_{\mathrm{RC}\rightarrow\mathrm{TS}} = V_{sub/Cu}^{TS} - V_{sub/Cu}^{RC}, \qquad \text{(S44)}$$

where $q_{sub/Cu}^{TS}(q_{sub/Cu}^{RC})$ and $V_{sub/Cu}^{TS}$ $(V_{sub/Cu}^{RC})$ stand for AIM charge and volume of the H-atom at the TS or RC, respectively.

In case of HAA reactions, these RC-to-TS descriptors offer only a slight distinction between PCET and HCET mechanisms. In both cases, these descriptors indicate an increase in the charge of the H-atom and a decrease in its volume: $\Delta q_{\mathrm{RC}\rightarrow\mathrm{TS}} > 0$ and $\Delta V_{\mathrm{RC}\rightarrow\mathrm{TS}} < 0$, which is consistent with partial H^+ formation (see **Figure S5**). However, the RC-to-TS change of charge is less pronounced for the HCET reactions, which suggests that the changes related solely to the mechanism of the reaction can be convoluted with changes common to both pathways. In both HCET and PCET reactions, the transition from RC to PC involves cleavage of the C–H bond and formation of the O–H bond. During this RC-to-PC process, the transferred hydrogen moiety exhibits a notable increase in charge (from ~0.05e^- to ~0.6e^-), accompanied by a decrease in volume (from ~34 a.u.^{3} to ~15 a.u.^{3}). Also, the charge on the hydrogen atom is influenced by polarization of the C–H bond during the RC-to-TS transition of the substrate geometry. As discussed in Ref S1, this effect can interfere with the net signature of H moiety transfer. These accompanying effects collectively influence the charge of the transferred

hydrogen at TS, potentially masking differences that are specific to the underlying reaction mechanism. We note in passing that the changes in charge and volume of the hydroxyl group during the previously studied OH rebound reactions[31] were considerably smaller ($0.08e^-$ and 9 a.u.^{3}, respectively) when going from RC to PC. This modest variation allowed for a more straightforward analysis of the reaction mechanism using the descriptors in equations (S43) and (S44).

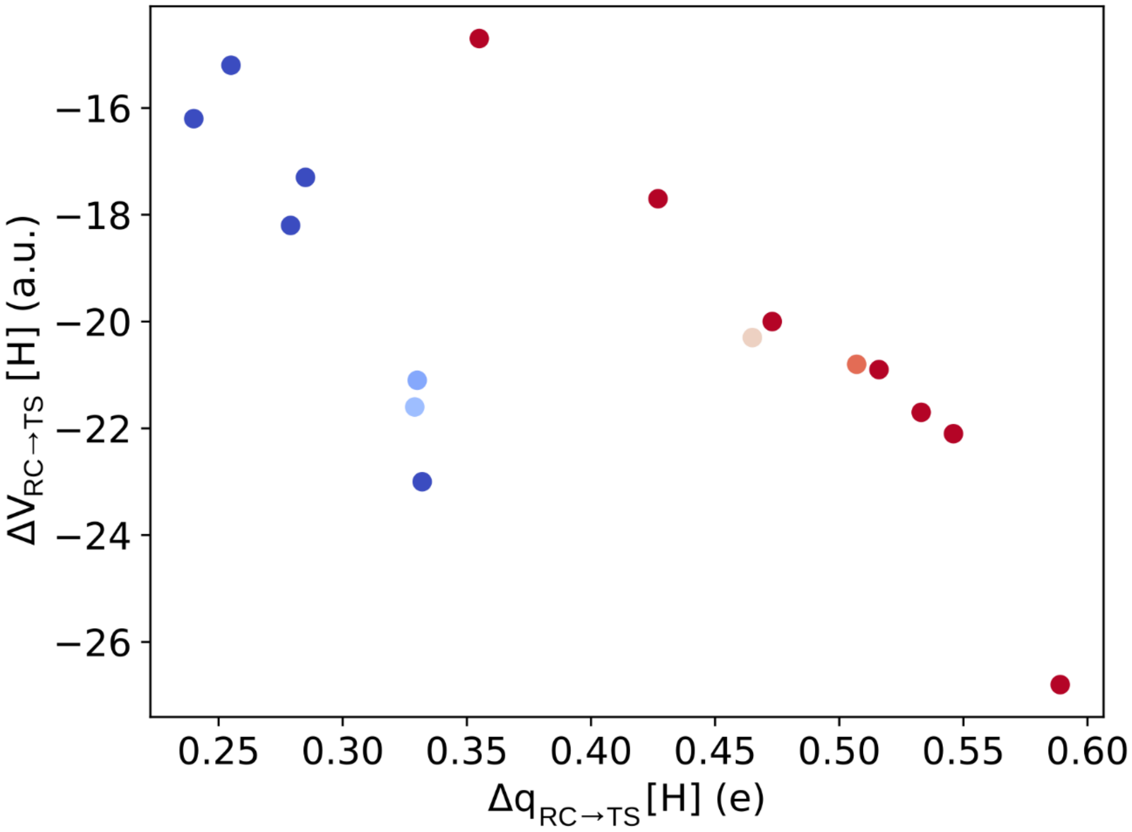

Figure S5. Volume and charge of the transferred H moiety at the TS relative to the volume and charge at RC (eqs (S43) and eqs (44)). The points are colored and shaded from dark blue (favored $H^{-}_{\rightarrow}/e^{-}_{\leftarrow}$) to dark red (favored $H^{+}_{\rightarrow}/e^{-}_{\rightarrow}$) to reflect the difference in off-diagonal thermodynamic contributions to the barrier, which originates from the two different $H^{-}_{\rightarrow}/e^{-}_{\leftarrow}$ and $H^{+}_{\rightarrow}/e^{-}_{\rightarrow}$ cycles presented in **Figure 3** in the main text.

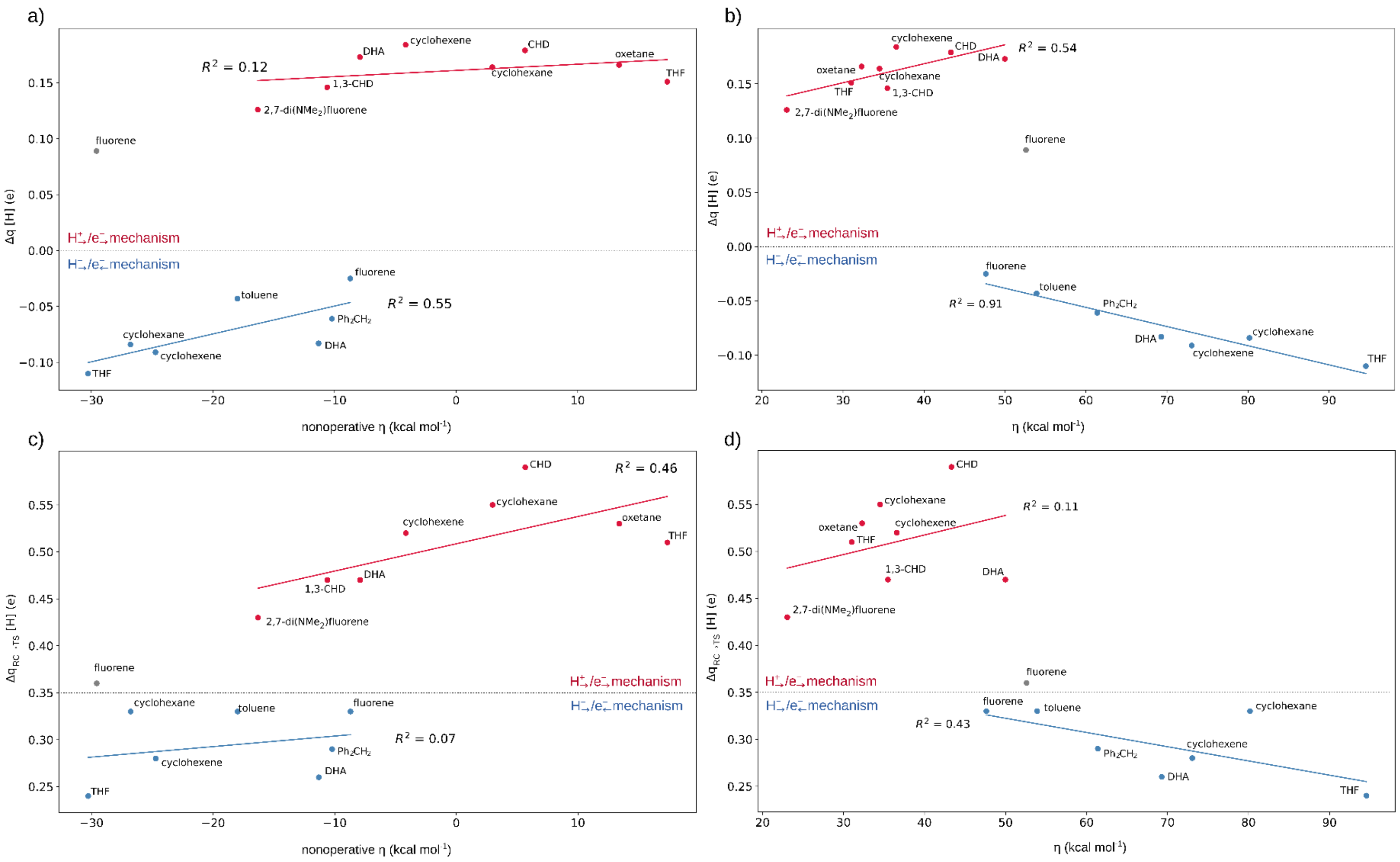

Figure S6. Correlations between η obtained from the nonoperative (*left*) and the operative thermodynamic cycle (*right*) and the deviation of charge on the transferred H atom at the TS calculated with respect to: the average of self-exchange reactions (*top*) or the RC (*bottom*). The points are colored to reflect the preference towards one of the thermodynamic cycles (as measured by the $\Delta G^{\neq}_{offdiag}$ originating from the $H^-_{\rightarrow}/e^-_{\leftarrow}$ and $H^+_{\rightarrow}/e^-_{\rightarrow}$ cycles) - in blue (favored $H^-_{\rightarrow}/e^-_{\leftarrow}$, the Cu(III)–OH set) and red (favored $H^+_{\rightarrow}/e^-_{\rightarrow}$, the Cu(II)–OH set).

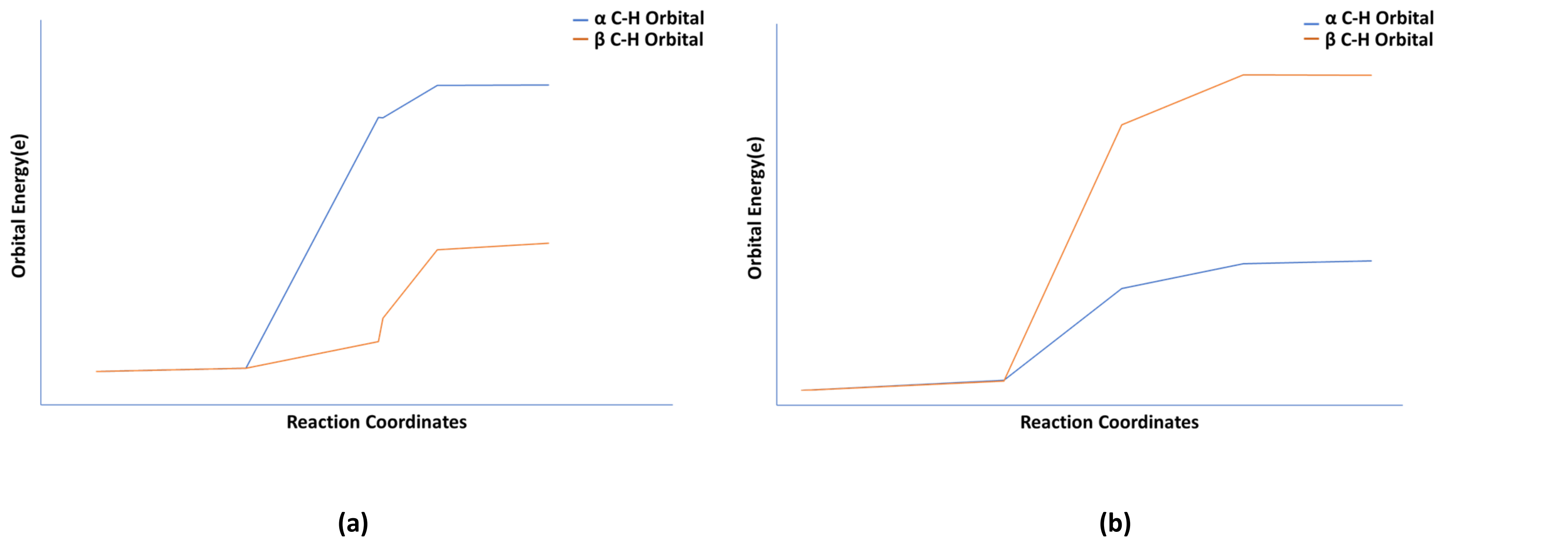

Figure S7. The evolution of IBOs initially describing the σ-orbital electron pair of the C−H bond along the IRC for (a) **Cu(III)–OH** with cyclohexane and (b) **Cu(II)–OH** with cyclohexane.

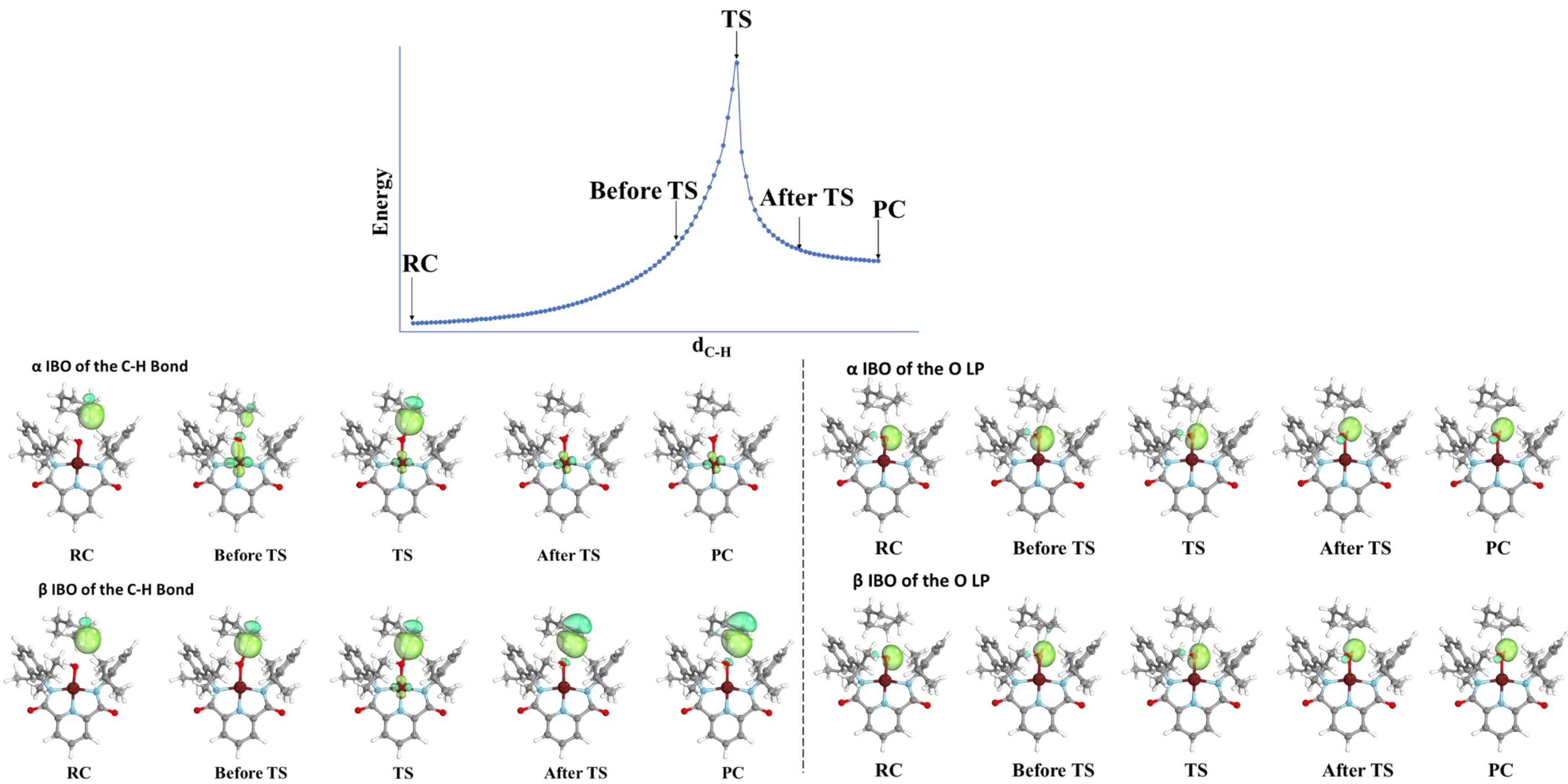
TS
Energy
Before TS
After TS
PC
RC
d_{C-H}
α IBO of the C-H Bond
RC
Before TS
TS
After TS
PC
β IBO of the C-H Bond
RC
Before TS
TS
After TS
PC
α IBO of the O LP
RC
Before TS
TS
After TS
PC
β IBO of the O LP
RC
Before TS
TS
After TS
PC

(a)

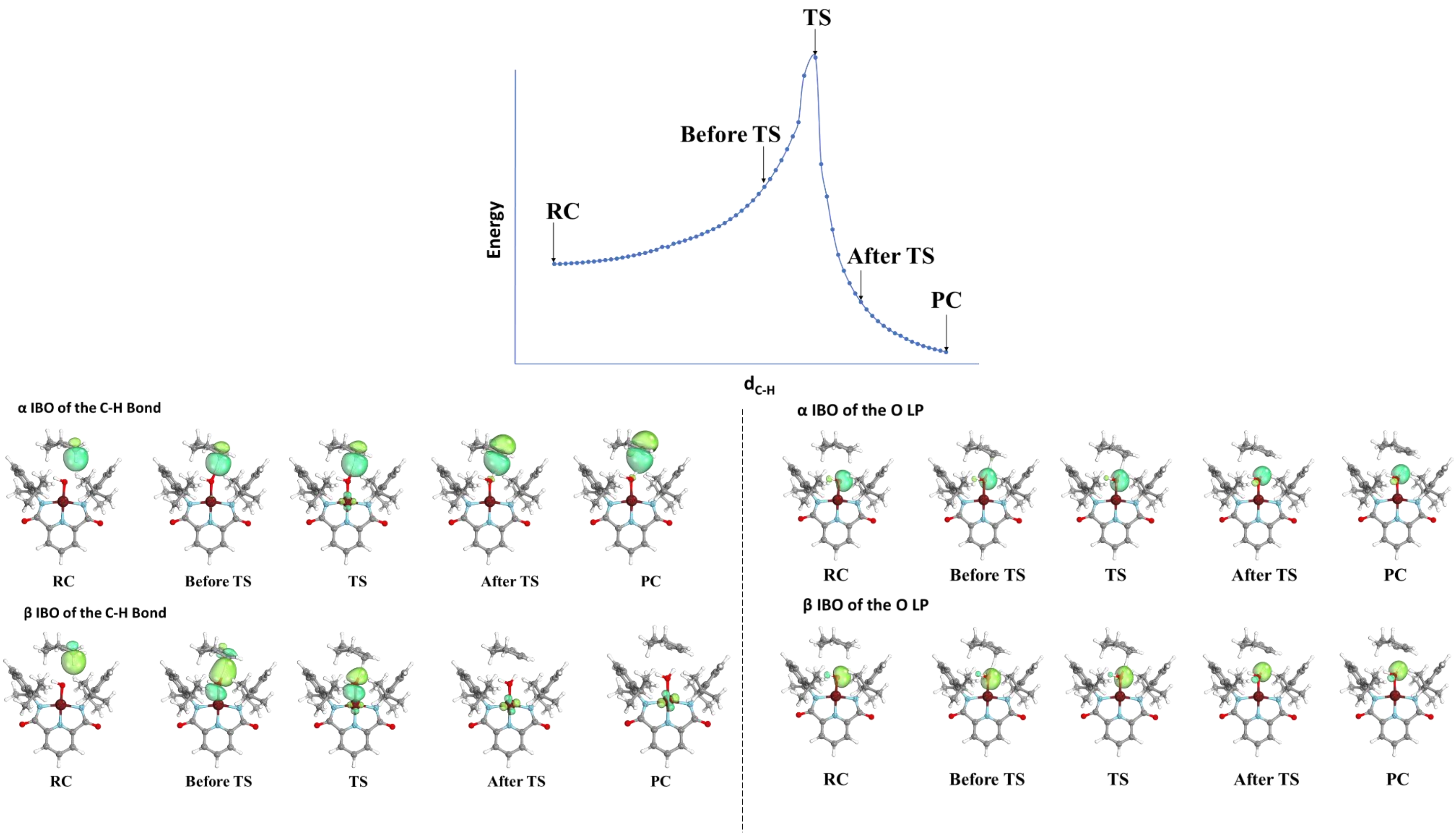
TS
Before TS
RC
After TS
PC
Energy
d_{C-H}
α IBO of the C-H Bond
RC
Before TS
TS
After TS
PC
β IBO of the C-H Bond
RC
Before TS
TS
After TS
PC
α IBO of the O LP
RC
Before TS
TS
After TS
PC
β IBO of the O LP
RC
Before TS
TS
After TS
PC

(b)

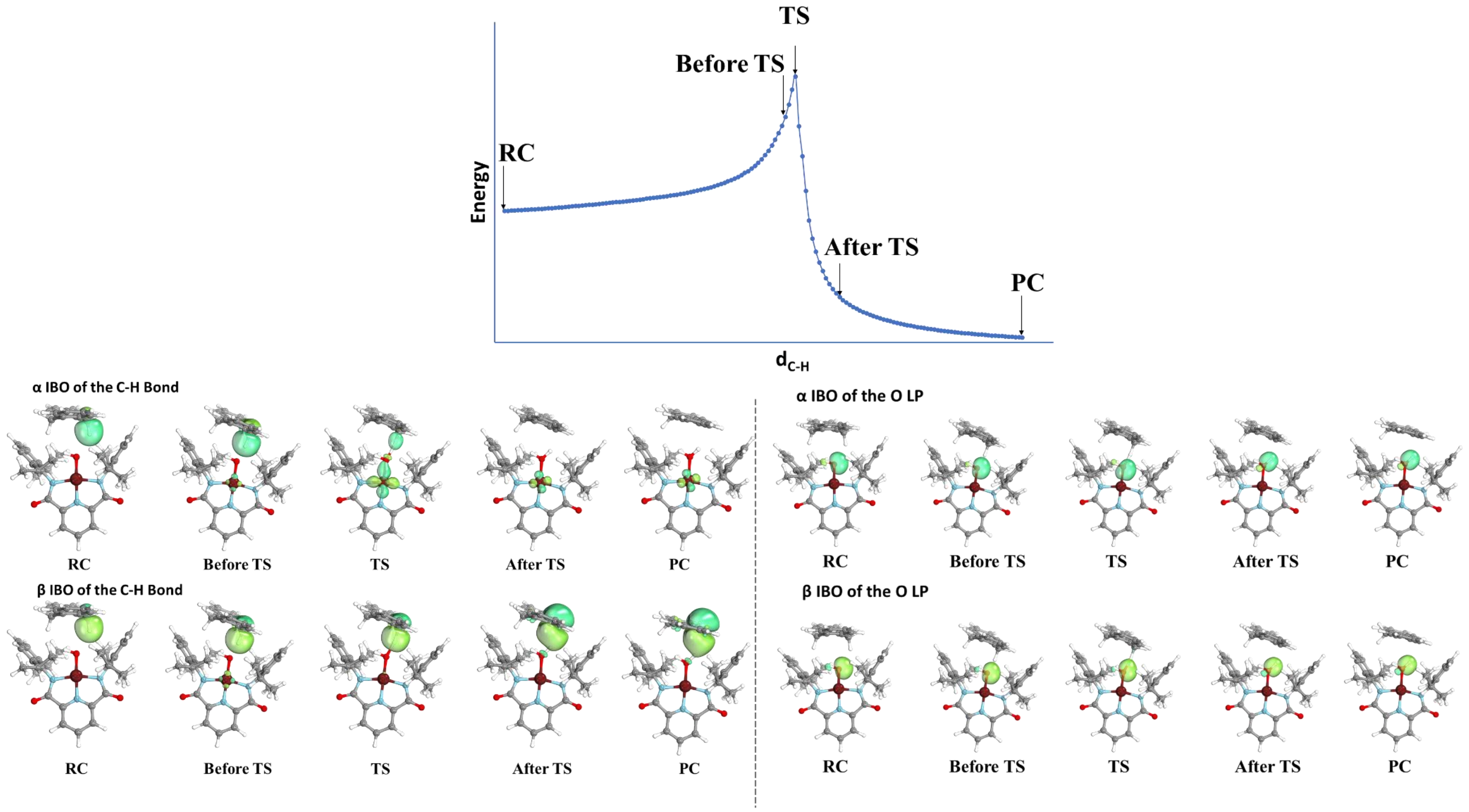
TS
Before TS
RC
Energy
After TS
PC
d_{C-H}
α IBO of the C-H Bond
RC
Before TS
TS
After TS
PC
β IBO of the C-H Bond
RC
Before TS
TS
After TS
PC
α IBO of the O LP
RC
Before TS
TS
After TS
PC
β IBO of the O LP
RC
Before TS
TS
After TS
PC

(c)

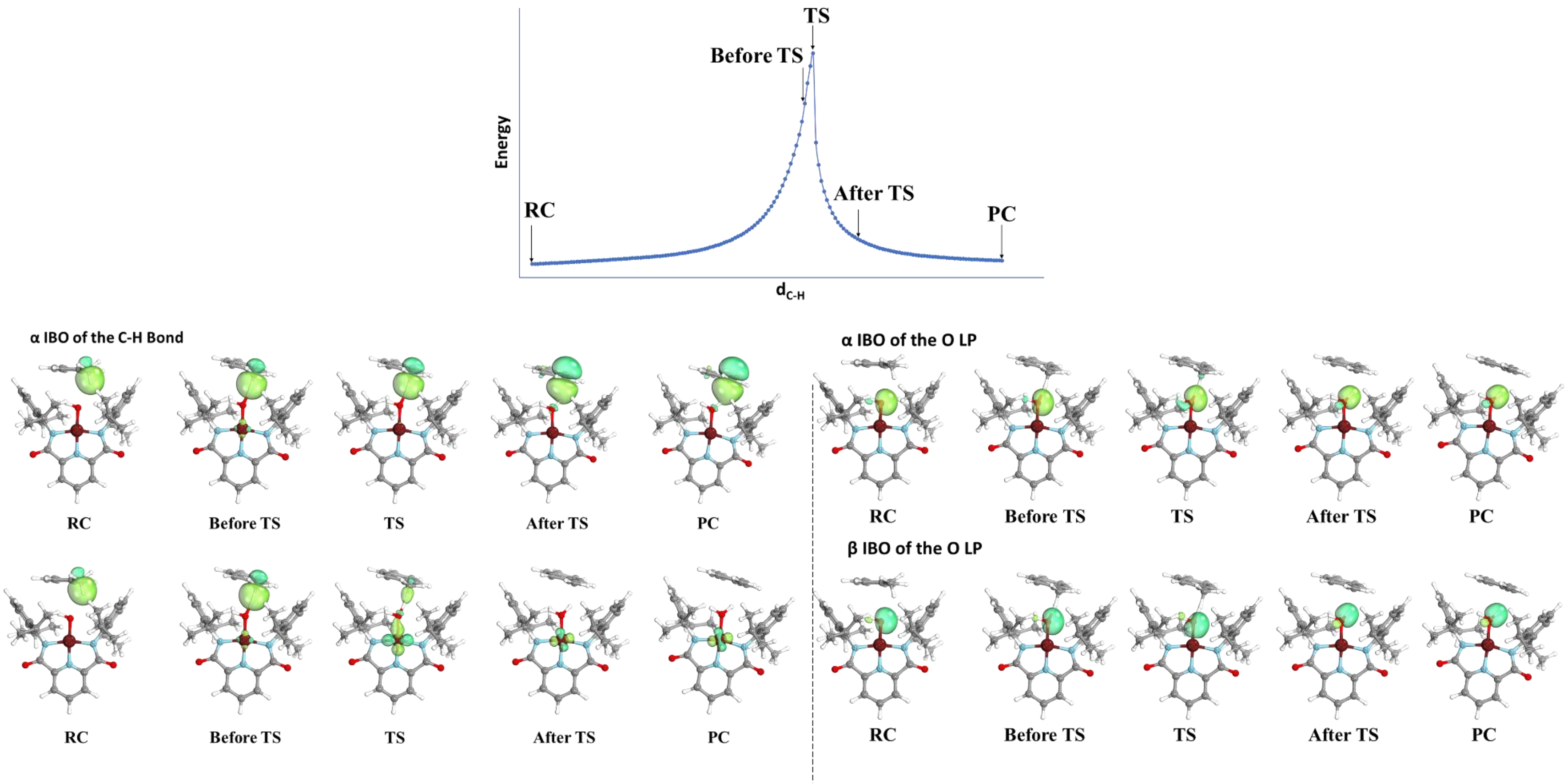

(d)

Figure S8. The intrinsic reaction coordinates (IRCs) and the important IBOs and their evolutions along the respective IRCs in the **Cu(III)–OH**-based reaction set. (a) Cyclohexane (b) Cyclohexene (c) DHA and (d) Toluene. For Toluene and DHA the *transient* ("*bouncing*") electron transfer occurs before the TS.

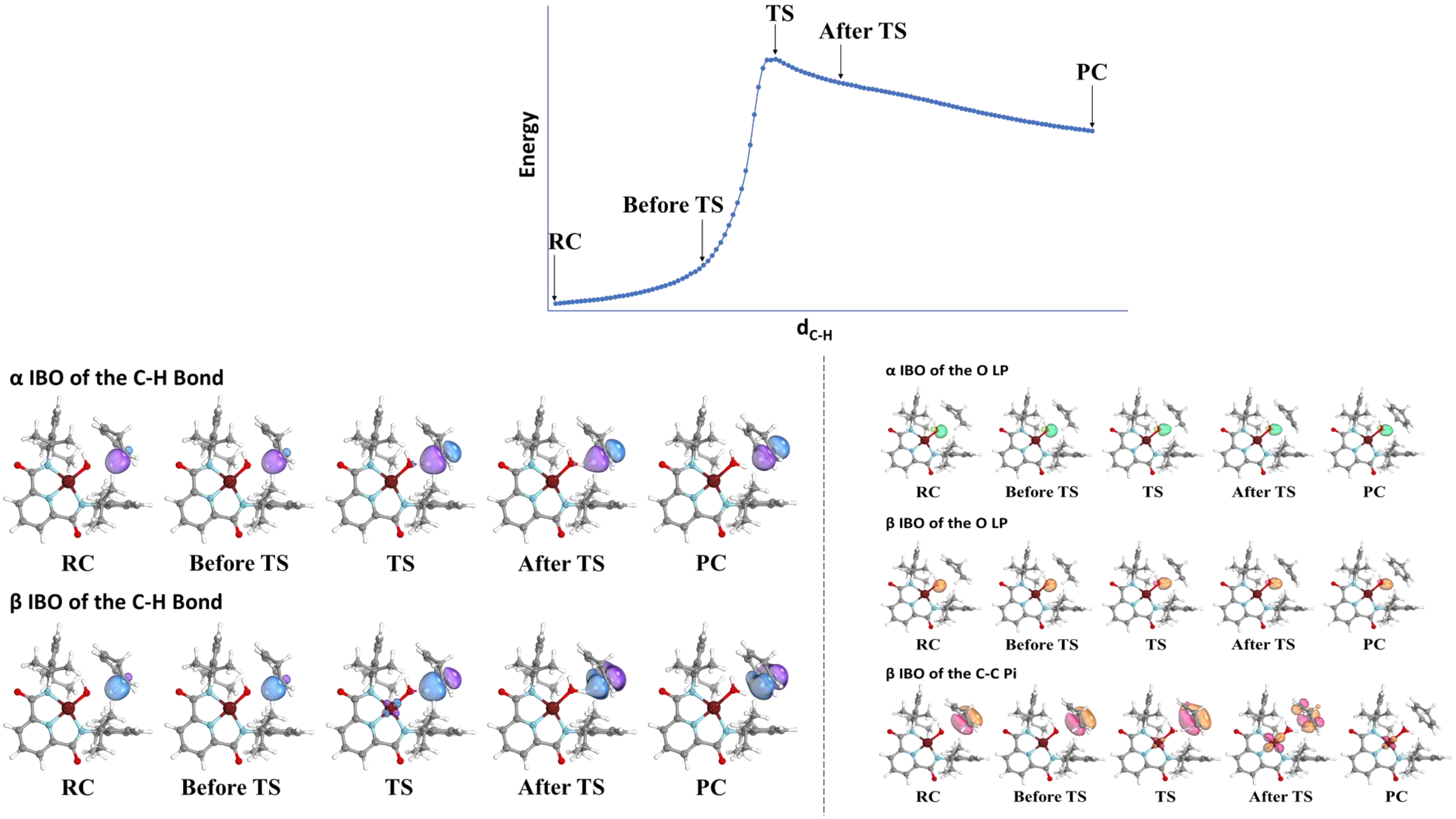

Energy
d$_{C-H}$
RC
Before TS
TS
After TS
PC
α IBO of the C-H Bond
RC
Before TS
TS
After TS
PC
β IBO of the C-H Bond
RC
Before TS
TS
After TS
PC
α IBO of the O LP
RC
Before TS
TS
After TS
PC
β IBO of the O LP
RC
Before TS
TS
After TS
PC
β IBO of the C-C Pi
RC
Before TS
TS
After TS
PC

(a)

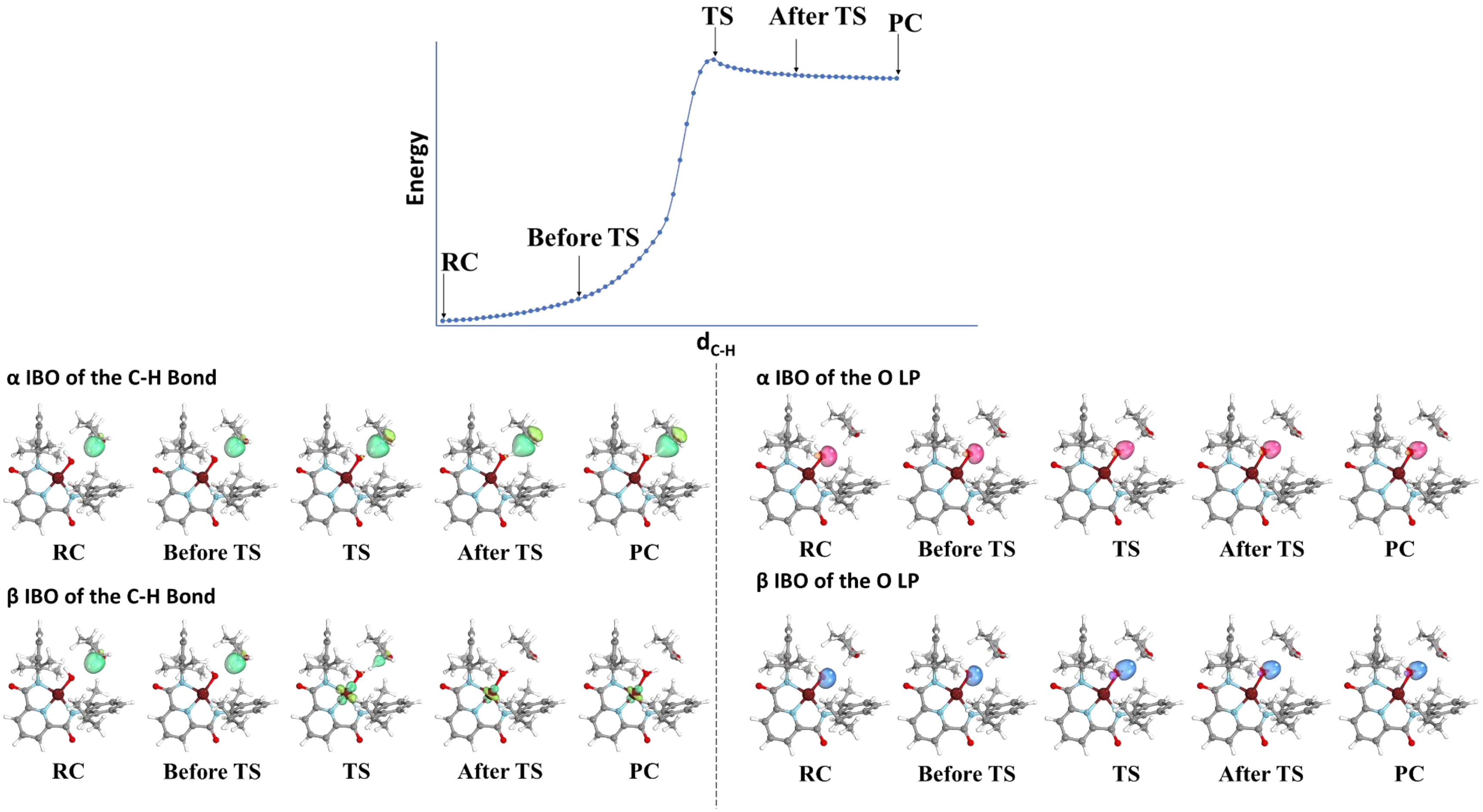

(b)

Figure S9. The intrinsic reaction coordinates (IRCs) and the important IBOs and their evolutions along the respective IRCs in the **Cu(II)–OH**-based reaction set. (a) 1,3-CHD and (b) THF. Exhibiting a normal PCET behaviour.

HAA involving para substituted phenols

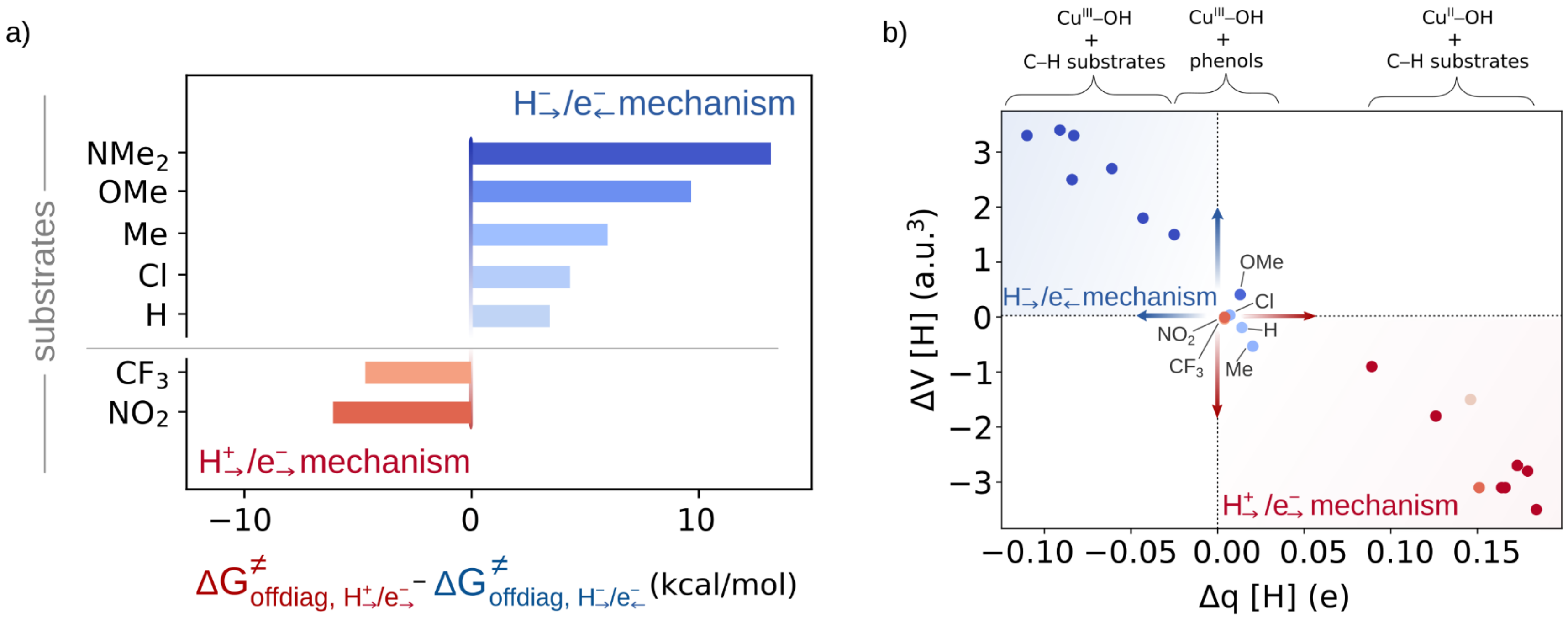

Figure S10. a) The difference between the off-diagonal contributions ($\Delta G^{\neq}_{offidiag}$ from eq (3)) associated with PCET and HCET thermodynamic cycles for the para-substituted phenols undergoing HAA with Cu^{III}–OH: negative values indicate preference for the PCET cycle (red), positive – for HCET (blue). b) Charge and volume of the transferred H moiety at the TS relative to the referential self-exchange values, as given by eqs (8) and (9) in the main text for all investigated systems: the C–H substrates and para-substituted phenols in HAA reactions with Cu^{III}–OH as well as the C–H substrates in HAA reactions with Cu^{II}–OH; the reactions with para-substituted phenols are labeled according to the substituent. The points are colored and shaded from dark blue (favored $H^{-}_{\rightarrow}/e^{-}_{\leftarrow}$) to dark red (favored $H^{+}_{\rightarrow}/e^{-}_{\rightarrow}$) to reflect the difference in off-diagonal thermodynamic contributions to the barrier, which originates from the two different $H^{-}_{\rightarrow}/e^{-}_{\leftarrow}$ and $H^{+}_{\rightarrow}/e^{-}_{\rightarrow}$ cycles presented in **Figure 3**.

In the CF_3-substituted phenol/Cu^{III}–OH system the reaction follows a PCET mechanism, though with slightly greater complexity than observed for the C-H substrates. Specifically, the β-electron from the delocalized π-bond in the substrate is transferred to the ligand arm of the oxidant, while simultaneously another β-electron is moving from that arm to the Cu center. While the reaction is formally described as a two-electron transfer, it effectively proceeds through a single-electron transfer from the substrate to the copper center. This transfer is facilitated by the orbitals of a coordinated ligand that engages in π-stacking interactions with the substrate's phenyl ring. Therefore, this can be classified as a ligand-mediated electron transfer; and a concise representation of the electron flow is given in **Figure 7** in the main text, while the complete electron evolution is provided in **Figure S11(b)**. The oxygen lone pair of the Cu complex participates in accepting the proton, forming an O–H bond.

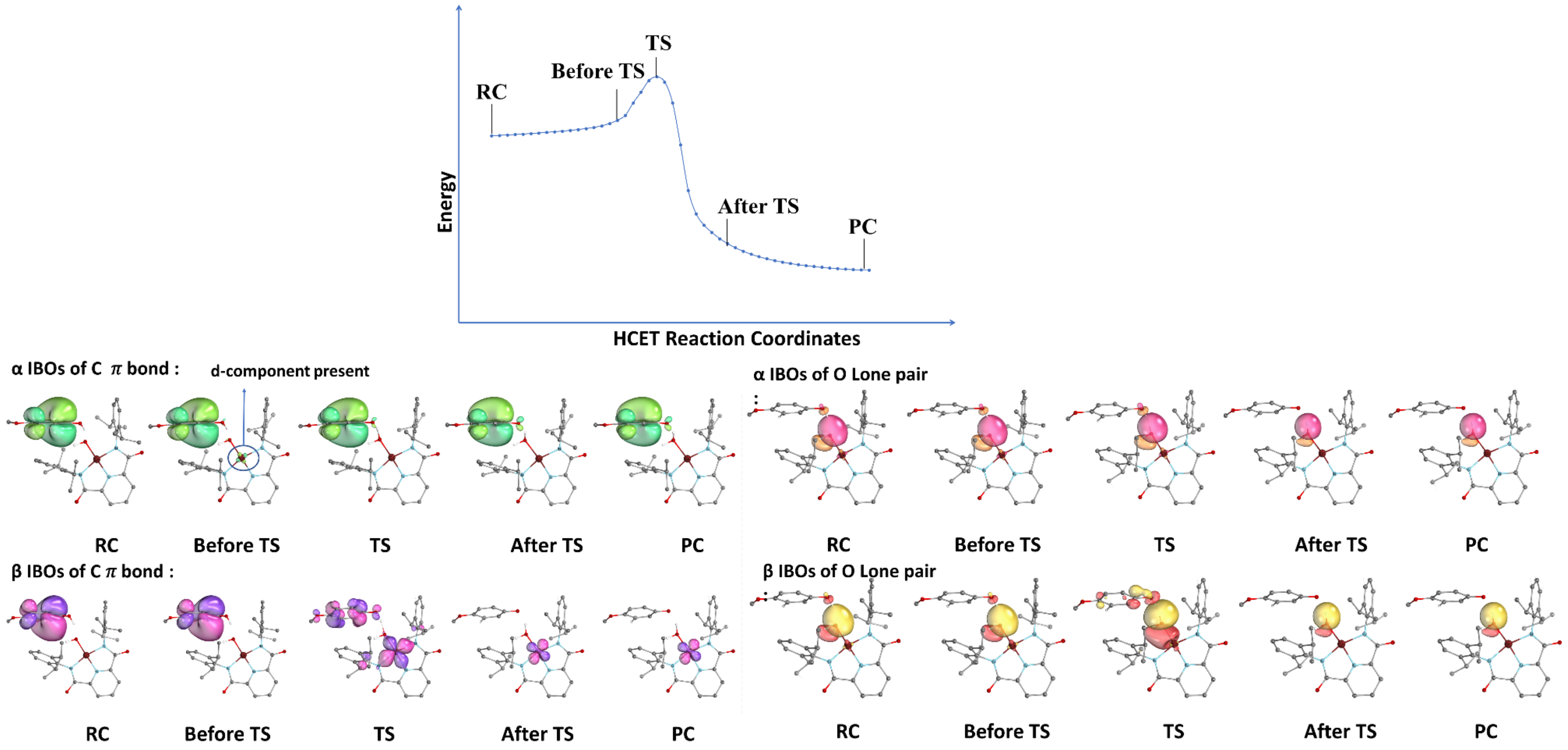
TS
Before TS
RC
After TS
PC
Energy
HCET Reaction Coordinates
α IBOs of C π bond :
d-component present
RC
Before TS
TS
After TS
PC
β IBOs of C π bond :
RC
Before TS
TS
After TS
PC
α IBOs of O Lone pair
RC
Before TS
TS
After TS
PC
β IBOs of O Lone pair
RC
Before TS
TS
After TS
PC

(a)

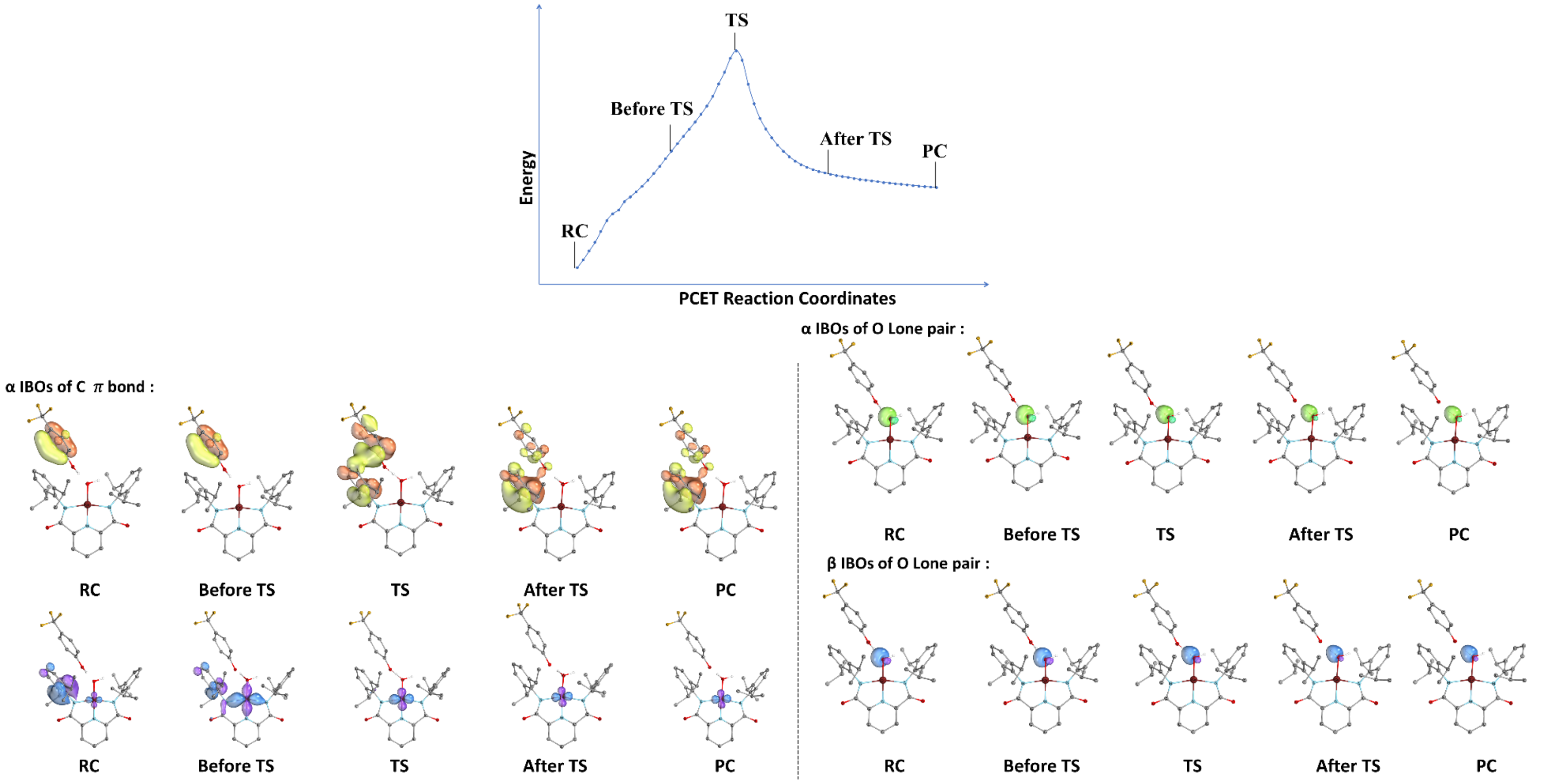

(b)

Figure S11. The intrinsic reaction coordinates (IRCs) and the important IBOs and their evolutions along the respective IRCs in the **Cu(III)–OH**-based reaction set. (a) *p*-methoxyphenol and (b) *p*-triflouromethylphenol. Exhibiting HCET and PCET behavior respectively. Note that in PCET, a formal two-electron transfer is described, but the process effectively involves a single-electron transfer from the substrate to the Cu center, mediated by the orbitals of the coordinated ligand. Also, the transient transfer in HCET is taking place before the TS, and the d-component in this step is highlighted for better clarity.

HAA involving the $[LNi^{II}OH]$ complexes

As shown in the main text, HCET involving the anionic Ni complex features two β-electrons moving concertedly and partially from the TEMPOH to the oxidant. This mechanism inherently reflects the potency of TEMPOH to lose two β-electrons and the relevant off-diagonal oxidized state of TEMPOH ($TEMPO^+$) must be in the triplet state. The ground state for the $TEMPO^+$ cation is $^1A'$ and the higher triplet state is separated vertically by 0.25eV, as reported in the photoelectron spectroscopy studies (Ref S2). However, taking into account the Gibbs free energy of the triplet state of TEMPOH allowed to correctly match the thermodynamic prediction with the mechanism observed in IBO analysis (as shown in **Table S6**) - for all $[LNi^{II}OH]$ complexes, including the S=1 $[LNi^{II}OH]^0$.

While our model predicts HCET to be electron-transfer-driven, this contrasts with the IBO analyses which indicate HCET to be hydride-driven. However, this inconsistency can be attributed to a computational artifact in which the one-electron-reduced TEMPOH decomposed into OH and an organic fragment. Importantly, this computational artifact does not impact the off-diagonal terms, because energetics of one of the off-diagonal states generally cancels in the calculation as we investigated previously (Ref S1).

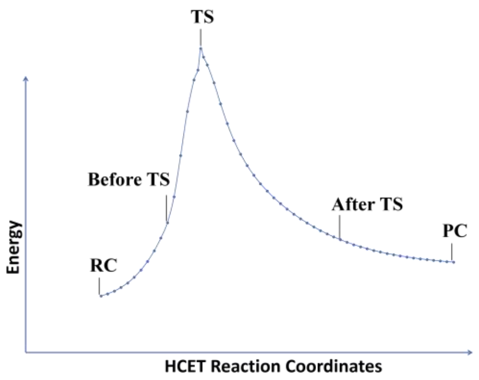
TS
Before TS
After TS
PC
RC
Energy
HCET Reaction Coordinates

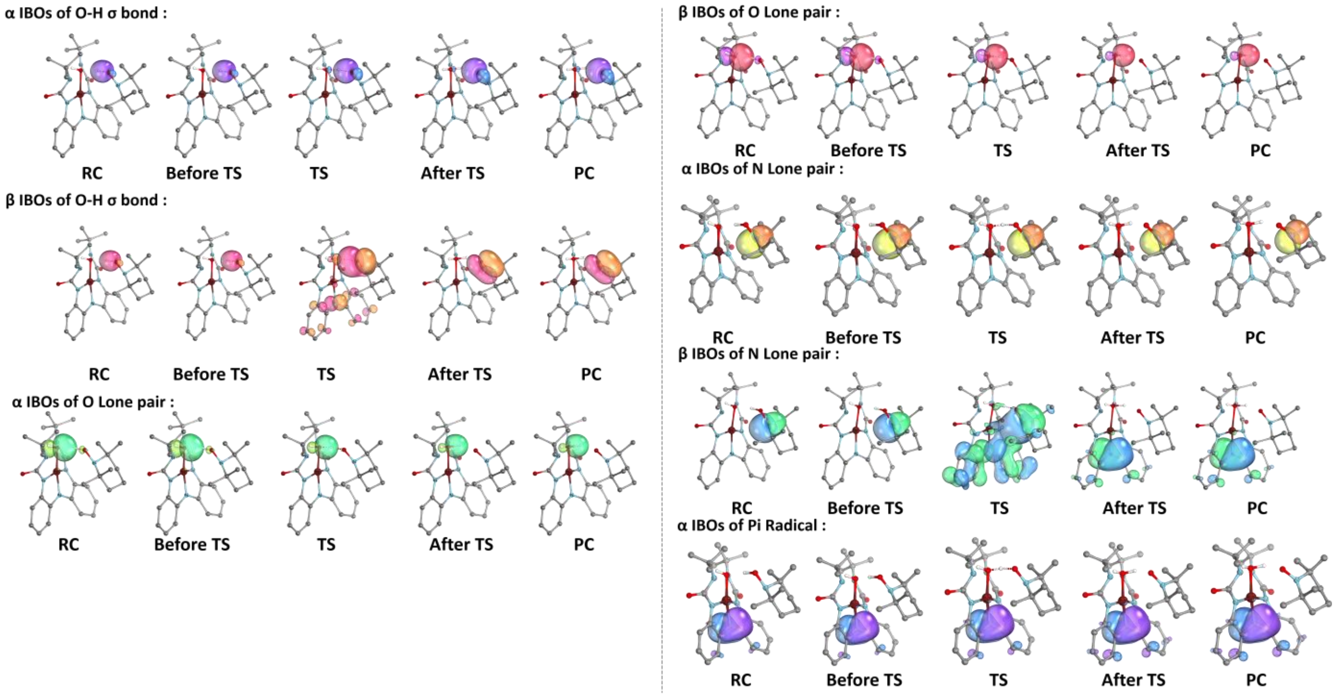
α IBOs of O-H σ bond :
RC
Before TS
TS
After TS
PC
β IBOs of O-H σ bond :
RC
Before TS
TS
After TS
PC
α IBOs of O Lone pair :
RC
Before TS
TS
After TS
PC
β IBOs of O Lone pair :
RC
Before TS
TS
After TS
PC
α IBOs of N Lone pair :
RC
Before TS
TS
After TS
PC
β IBOs of N Lone pair :
RC
Before TS
TS
After TS
PC
α IBOs of Pi Radical :
RC
Before TS
TS
After TS
PC

(a)

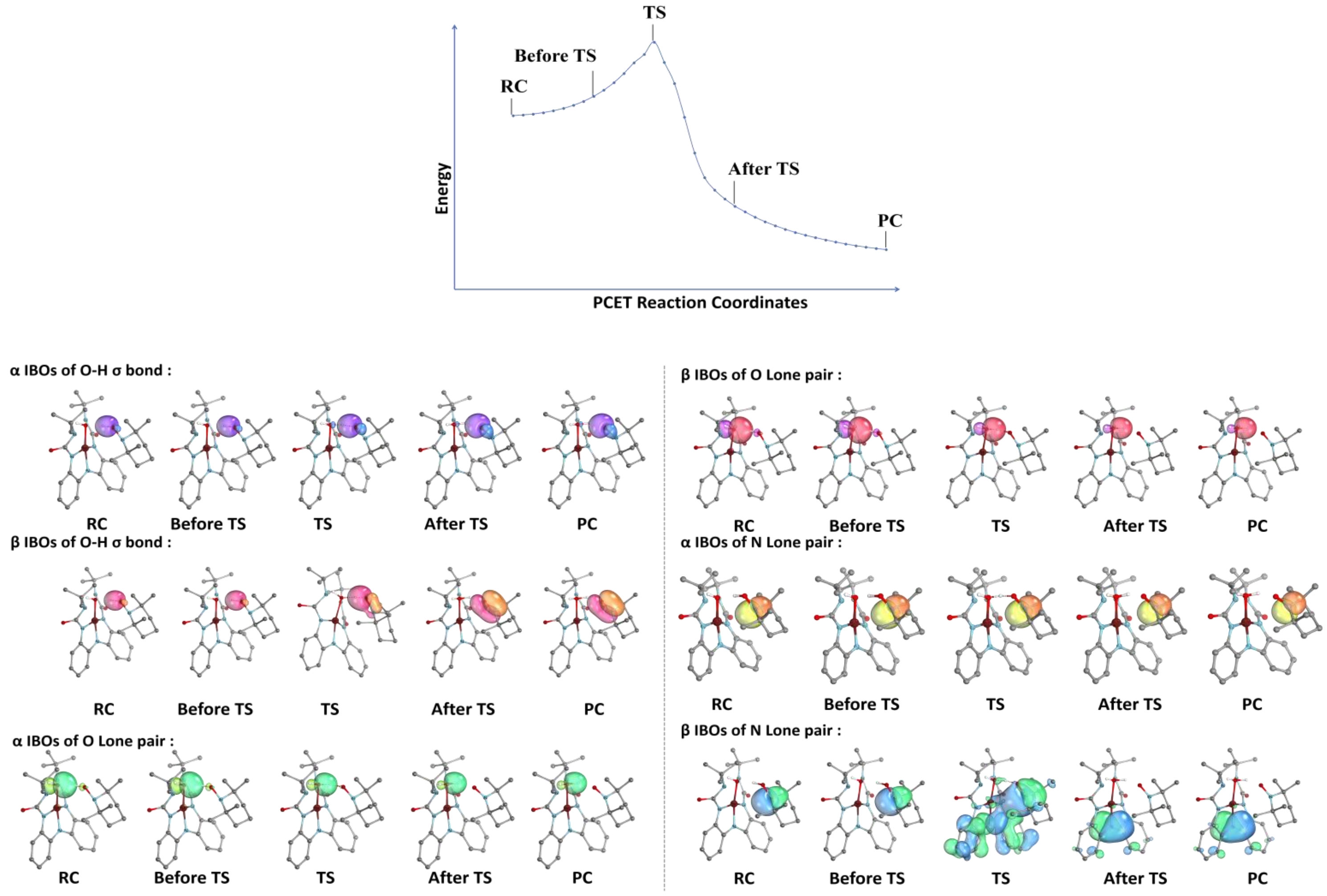

(b)

Figure S12. The intrinsic reaction coordinates (IRCs) and the important IBOs and their evolutions along the respective IRCs in the reactions of the **Ni(II)–anionic** (a) and **Ni(II)–neutral** (b) systems with TEMPOH. Exhibiting HCET and PCET behavior respectively.

IBO analysis of the OH rebound reaction

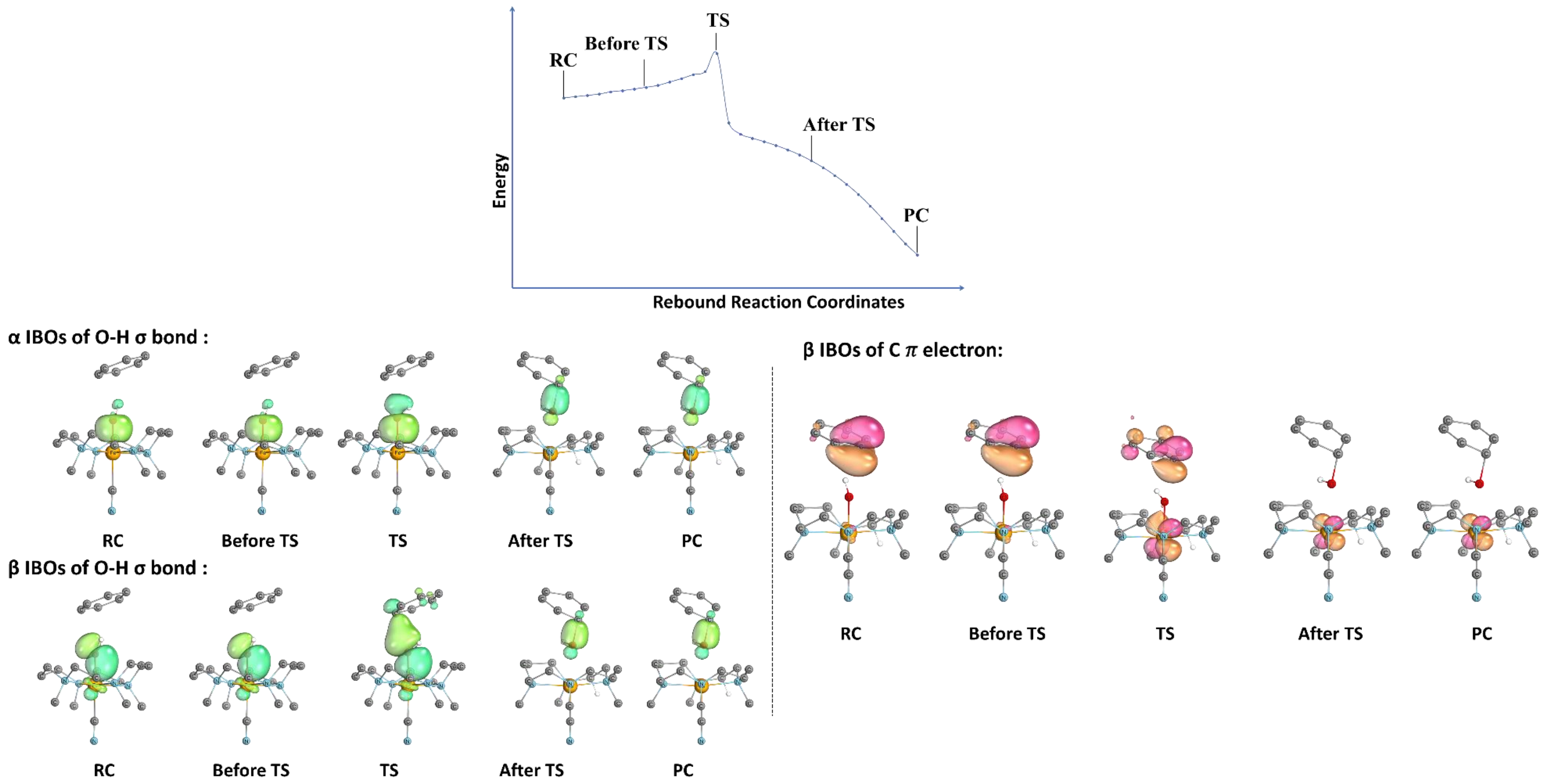

Figure S13. The IRC and the important IBOs and their evolutions along the IRC in the **Fe(III)–OH**-based rebound reaction with cyclohexadiene radical. Showing a hydroxide coupled electron transfer.

Structural features of the transition states and *adiabaticity* of the reaction. Comparison of geometries of the transition states for Cu^{III}–OH and Cu^{II}–OH systems reveals that, as it can be expected for Cu^{II}–OH, the strongly hindered, endoergic reactions feature late TSs, with an elongated C–H bond and a short O–H bond (nearly formed). From a mechanistic perspective, the geometry of the transition state is somewhat reflective of the asynchronicity of the reaction, as reactions more asynchronous towards H^+ transfer feature more elongated C–H bond and a shorter H–O distance.

On the other hand, in the Cu^{III}–OH set, the H atom is located at the midpoint between the donor (C atom) and the acceptor (O of Cu^{III}–OH). This characteristic is in line with lower ΔG_0 and $\Delta G^{\neq}$ values for the reactions but also indicates that H^-/e^- transfer features stronger interactions between the substrates and the H-atom acceptor, possibly reflecting a larger adiabatic coupling. Stronger interactions are evidenced by the shorter sum of C–H and H–O bond lengths at the respective TSs as shown in **Figure S14**.

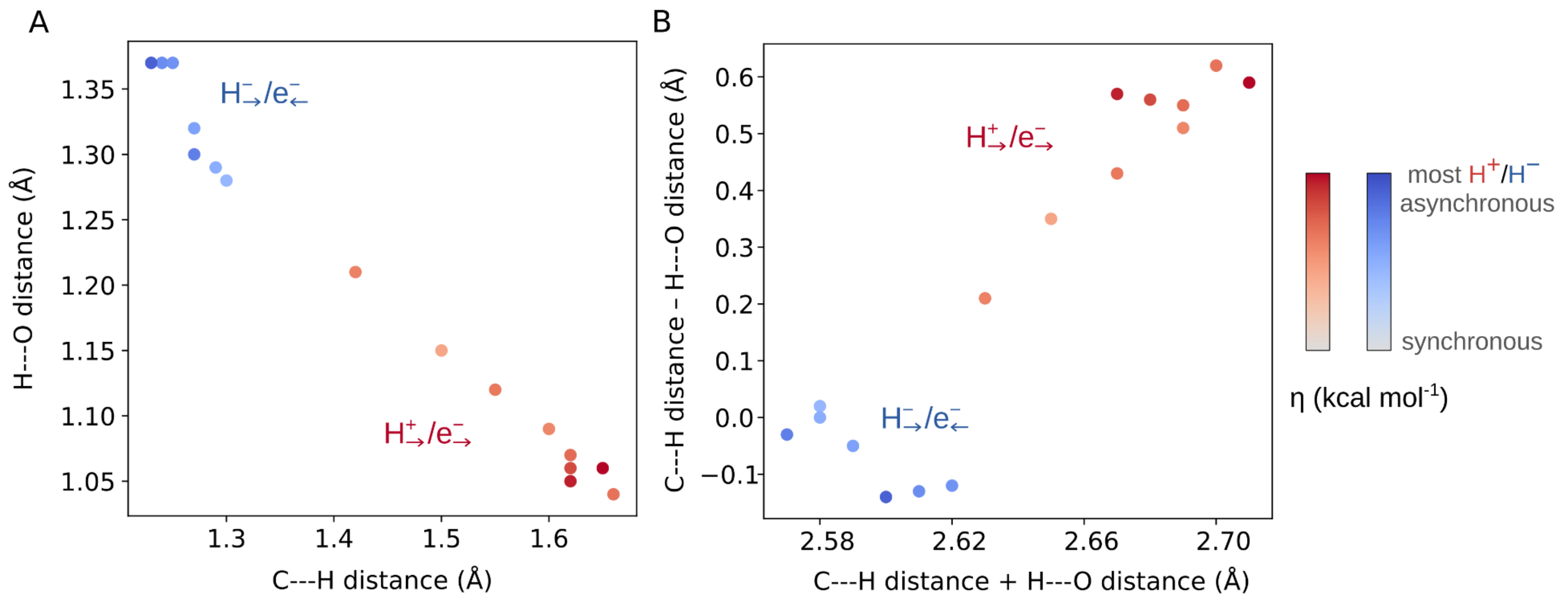

Figure S14. The crucial C–H and H–O distances at the TS, the points are colored and shaded from grey to dark blue to reflect the asynchronicity of the $H^-_{\rightarrow}/e^-_{\leftarrow}$ reaction the and from grey to red to reflect asynchronicity of the $H^+_{\rightarrow}/e^-_{\rightarrow}$ reaction.

References in SI:

(S1) Wojdyla, Z.; Maldonado-Domínguez, M.; Bharadwaz, P.; Culka, M.; Srnec, M. Elucidation of Factors Shaping Reactivity of 5′-Deoxyadenosyl – a Prominent Organic Radical in Biology. *Phys. Chem. Chem. Phys.* **2024**, *26* (30), 20280–20295.

(S2) Novak, I.; Harrison, L. J.; Kovač, B.; Pratt, L. M. Electronic Structure of Persistent Radicals: Nitroxides. *J. Org. Chem.* **2004**, *69* (22), 7628–7634.
